# Chemodiversity of Cyanobacteria from Brazil Investigated by Metabolomics and Bioassays

**DOI:** 10.1021/acsomega.5c07322

**Published:** 2025-10-24

**Authors:** Francisco H. S da Silva, Leonardo S de Jesus, Michael J. J. Recchia, Kleyton J. G. de Morais, Camila M. C. Gonçalves, Sandra R. C. Soares, Helori V. Domingos, Hannah Cavanagh, Frederico J. Gueiros-Filho, José A. L. Lindoso, Leticia V. Costa-Lotufo, Roger G. Linington, Roberto G. S. Berlinck, Camila M. Crnkovic

**Affiliations:** † Faculdade de Ciências Farmacêuticas, Universidade de São Paulo, São Paulo 05508-000, São Paulo, Brazil; ‡ Department of Chemistry, 1763 Simon Fraser University, Burnaby V5A 1S6, British Columbia, Canada; § Instituto de Química de São Carlos, Universidade de São Paulo, São Carlos 13560-970, São Paulo, Brazil; ∥ Laboratório de Prozoologia, Instituto de Medicina Tropical, Faculdade de Medicina, Universidade de São Paulo, São Paulo 05403-000, São Paulo, Brazil; ⊥ Departamento de Farmacologia, Instituto de Ciências Biomédicas, 28133 Universidade de São Paulo, São Paulo 05508-000, São Paulo, Brazil; ▲ Instituto de Infectologia Emílio Ribas, São Paulo 01246-900, São Paulo, Brazil

## Abstract

Investigations on cyanobacterial secondary metabolites in Brazil have been scarce, despite the country’s significant biodiversity. Herein, we report the results of a screening of cyanobacterial cultures using both bioassays and untargeted metabolomics. Nineteen cyanobacterial strains collected at various locations in Brazil were cultured. Cultures were extracted and prefractionated. Fractions were evaluated for antibacterial, cytotoxic, and antileishmanial activities. The same fractions were analyzed by UHPLC–HRMS–MS/MS. Results from bioassays and LC–MS were integrated using metabolomics tools such as NP Analyst and GNPS Molecular Networking, allowing for feature prioritization. Cyanobacteria belonging to genera *Calothrix* and *Phormidium* presented high-priority molecular features associated with observed biological activities, indicating that such strains are producers of potentially novel and bioactive metabolites.

## Graphical abstract

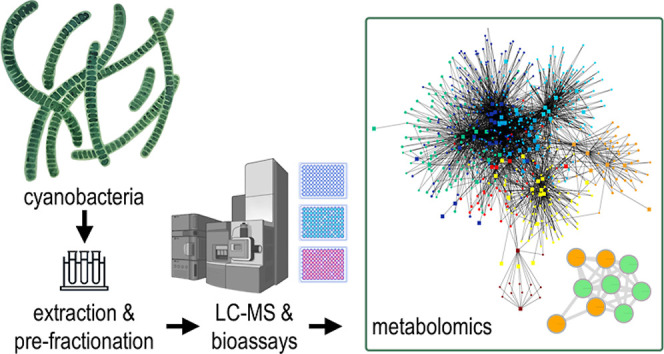

## Introduction

Cyanobacteria are a diverse phylum that remains relatively understudied from a biotechnological perspective.^1^ Although often associated with the production and accumulation of toxins, cyanobacteria also represent a rich source of bioactive metabolites with applications in food, cosmetics, and pharmaceuticals.^2,3^ Bioactive secondary metabolites isolated from cyanobacteria have shown antimicrobial, antitumor, antiparasitic, and others therapeutically relevant activities.^4,5^ Dolastatin 10, for example, inspired the development of several antibody–drug conjugates, such as brentuximab vedotin, and new functional analogs.^6^

Covering an area of 8,510,000 km^2^, Brazil boasts approximately 20% of the world’s biodiversity.^7,8^ However, only a few natural products have been reported from cyanobacteria collected in Brazil. These include namalides B and C, spumigins K–N, and some aeruginosins.^9–11^ In the search for new natural products, metabolomics emerged as a powerful tool to aid bioassay-guided fractionation, biological sample prioritizations, bioactivity prediction for secondary metabolites, and early dereplication during the isolation of new natural products.^12,13^ Recently, the use of metabolomic tools in screening the toxicological and bioactive potential of Brazilian cyanobacteria has been increasingly explored, highlighting how platforms such as GNPS,^14^ NP Analyst,^15^ and DAFdiscovery^16^ enable high-throughput, in-depth metabolomic analyses, particularly in light of the diverse Brazilian ecosystems.^10,17–19^

Here, we report the use of bioassays and untargeted metabolomics to explore the chemical potential of Brazilian cyanobacteria. We screened fractions of Brazilian cyanobacteria cultures for antibacterial, cytotoxic, and antileishmanial activities, employing LC–MS to prioritize biologically relevant features and facilitate dereplication of secondary metabolites.

## Materials and Methods

### Biological Material and Culture Conditions

Nineteen strains of cyanobacteria were obtained from the Collection of Cyanobacteria Cultures of the Institute of Botany (CCIBt)—São Paulo state, curated by Prof. Célia Leite Sant’Anna (Sisgen registration numbers A531C68 and A8EDA53) (Supporting Information Table S1). Each strain was transferred to passage cultures (150 mL), using BG-0 or BG-11 media (Supporting Information Table S1),^20,21^ that served as inoculum for larger-scale cultures. Larger-scale cultures were grown in a total of 4.5 L of media and divided equally into three 2 L Erlenmeyer flasks. Cultures were kept at 24 °C, under sterile aeration and irradiance of 30 μmol photons m^–2^ s^–1^ in a 12–12 h light–dark cycle.

### Extraction and Prefractionation

After 8 weeks of culture, cyanobacterial cells were separated from the culture medium by centrifugation and lyophilized. The lyophilized cell mass was extracted by three cycles of maceration with CH_2_Cl_2_/MeOH (1:1). Extracts were concentrated in a rotatory evaporator under 37 °C. The dried extracts were then prefractionated by solid-phase extraction using Diaion HP-20SS as the stationary phase and a step-gradient of isopropanol (IPA) in water (H_2_O): F1 (0:100), F2 (20:80), F3 (40:60), F4 (70:30), F5 (90:10), and F6 (100:0).^22^ Supporting Information Table S2 displays the obtained mass of extracts and fractions. All samples (extracts and fractions) were initially dissolved in MeOH to obtain a stock solution at 10 mg/mL. Subsequently, 10 μL aliquots (corresponding to 100 μg of the sample) were transferred and dried for biological assays.

### Cytotoxic Cell Assay

Extracts and fractions were evaluated against the cancer cell lines HCT-116 (colon carcinoma) and MCF-7 (breast cancer), using the MTT assay.^23^ Briefly, 2 × 10^3^ cells per well, in 96-well plates (1 × 10^4^ cells/mL in 200 μL medium) for the HCT-116 strain and 8 × 10^3^ cells per well, in 96-well plates (4 × 10^4^ cells/mL in 200 μL medium) for the MCF-7 strain, were plated. Dry samples were resuspended in DMSO to prepare a stock solution, which was then diluted in the cell culture medium to yield the final test concentrations of 50 and 5 μg/mL, with 0.5% DMSO in a total volume of 200 μL. After 24 h of growth, cells were exposed to extracts and fractions in duplicate. Subsequently, exposed cells were incubated without oxygen for 72 h in 5% CO_2_. After the incubation period, the supernatant was replaced with a culture containing MTT (0.5 mg/mL). 3 h later, the supernatant was removed, and after drying the plate, the precipitate containing formazan blue was dissolved in 150 μL of DMSO, and absorbance was measured at 570 nm. Doxorubicin and DMSO were used as positive and negative controls, respectively. Single-well data were transformed into cell viability percentages after normalization with negative control (100% growth) and blanks (no cell wells, 0%). Samples were considered cytotoxic when they inhibited cell growth of ≥70% at 50 μg/mL or ≥50% at 5 μg/mL.

### Antibacterial Activity Assay

Antibacterial activity assays were conducted in triplicate compliant with the Clinical and Laboratory Standards Institute^24^ against *Escherichia coli* (ATCC 25922), *Klebsiella quasipneumoniae* (ATCC 700603), *Pseudomonas aeruginosa* (ATCC 27853), *Staphylococcus aureus* (ATCC 29213), and *Serratia marcescens* (ATCC 14764). Isolated colonies were inoculated in cation-adjusted Mueller–Hinton broth. Bacterial suspensions were prepared from isolated colonies grown on Mueller–Hinton agar (18–24 h, 37 °C), adjusted to the 0.5 McFarland standard (∼1.5 × 10^8^ cfu/mL), and subsequently diluted in cation-adjusted Mueller–Hinton broth to obtain a final concentration of 5 × 10^5^ cfu/mL per well. The dried samples were resuspended in DMSO so that 3.3 μL of sample solution added to each well produced a final concentration of 50 μg/mL (3.3% DMSO). In the assays, 96.7 μL of the standardized inoculum was distributed into each well, followed by the addition of 3.3 μL of the prepared samples. Negative controls contained only DMSO (3.3%), while meropenem (100 μg/mL) was used as a positive control. Growth controls contained only the inoculum in the Mueller–Hinton broth. Plates were incubated at 37 °C for 18–24 h. Bacterial growth was monitored by measuring the optical density at 600 nm using an Epoch microplate reader. Growth inhibition was expressed relative to the negative control, and samples were considered bioactive when they inhibited ≥70% of bacterial growth at 50 μg/mL.

### Antileishmania Activity Assay

*Leishmania* *amazonensis* promastigotes in the stationary phase were centrifuged at 3600*g* for 10 min at 20 °C, resuspended in phenol-free RPMI 1640 medium with 10% fetal bovine serum, and plated in 96-well plates at 10^7^ parasites/μL (100 μL per well). A total of 50 μg of the sample were resuspended in 10 μL of DMSO and diluted in 990 μL of RPMI medium. Then, 100 μL of this solution was added to 100 μL of the *L. amazonensis* suspension, yielding a final concentration of 25 μg/mL and 0.5% DMSO. Cyanobacterial extracts and fractions were tested in quintuplicate in the *L. amazonensis* growth medium, followed by 48 h of incubation at 26 °C.^25^ Promastigotes in medium served as the negative control, while DMSO at 100 μM (diluted in RPMI 1640) was the positive control. After 24 and 48 h of incubation, viability was assessed with Alamar blue.^26^ Plates were centrifuged, the supernatant was removed, and resazurin solution (10% of medium volume) was added. After an additional 24 h of incubation at 26 °C, absorbance was read at 690 and 600 nm using an ELISA spectrophotometer, with viability expressed as a percentage relative to the negative control. Samples were considered bioactive when they inhibited parasite growth of ≥70% at 25 μg/mL.

### LC–MS Analyses

In parallel to bioassay testing, all fractions were analyzed by ultrahigh performance liquid chromatography hyphenated to high-resolution mass spectrometry and tandem mass spectrometry (MS/MS). Liquid chromatography–mass spectrometry (LC–MS) data were acquired using Waters Acquity UPLC H-class equipment, coupled to a Waters Xevo G2-XS QToF mass spectrometer with electrospray ionization (ESI) in the total ion scan mode, operating in data-dependent analysis. Chromatographic separation was performed using a 2.1 × 50 mm Kinetex C18 column (Phenomenex) with 1.7 μm particles. The mobile phase consisted of a mixture of LC–MS-grade H_2_O (A) and MeCN (B), both acidified with 0.1% formic acid. The gradient program employed a linear increase from 10% to 100% B over 7 min, followed by 1 min at 100% B, a rapid return to 10% B within 0.1 min, and a final hold at 10% B for 1.9 min. The operating parameters of the equipment were as follows: capillary voltage of 1200 V, cone voltage of 30 eV, ion source temperature at 100 °C, desolvation temperature at 450 °C, the nitrogen (N_2_) gas flow rate in the cone at 50 L/H, the desolvation gas flow rate at 750 L per hour, the mass detection range from 100 to 2000 Daltons, 0.2 s sweep time, and collision energy ranging from 15 to 30 eV and 60 to 80 eV on ramp. To obtain the fragmentation spectra (MS/MS), the three most intense ions in each MS1 spectrum were selected for fragmentation.

### HPLC–UV–MS Analyses

Anabaenopeptins were analyzed using a Waters HPLC–UV–MS system (2695 Alliance module, 2696 PDA detector, and Micromass ZQ2000 MS). Separation was performed on an XTerra RP18 column (250 × 4.6 mm, 5 μm) with an RP18 guard column (4 × 3 mm), using a linear gradient (10–100%) of the organic phase (1:1 MeOH/MeCN, both with 0.1% formic acid) over 30 min. The aqueous phase also contained 0.1% formic acid. The flow rate was 1.0 mL/min. UV detection was carried out at 200–700 nm. Mass spectrometry was operated in ESI positive and negative modes (*m*/*z* 400–1600), with the following settings: 3 kV capillary voltage, 100 °C source temperature, 350 °C desolvation temperature, cone gas flow 50 L/h, and desolvation gas flow 350 L/h. Authentic standards and fractions were analyzed individually. Spiking experiments with standards and fractions were performed to confirm the retention times and MS profiles.

### Metabolomics and Dereplication NP Analyst

LC–MS data were imported into Progenesis QI software (Waters, Milford, MA, USA) for alignment, peak selection, normalization, and deconvolution, using the parameters of minimum intensity of 100,000 and a minimum width of 0.1 min. Processed data files and the corresponding .csv metadata files (containing compound intensities and annotations) were exported and reformatted for upload to NP Analyst using an in-house Python script.^15^ Bioactivity results were combined in a single csv file. Data were binned and normalized (0 to 1 score) using the following parameters: bioactivity values between 0 and 0.25 = 0; values between 0.25 and 0.7 = 0.5; values between 0.7 and 1 = 1. Metabolomics and binned bioactivity data sets were analyzed by the NP Analyst pipeline for compound activity mapping, using an activity score of 2 and a cluster scores of 0.5 (for definitions of activity and cluster score see ref 15). Results allowed for the detection of features potentially associated with the observed activity.

### GNPS Molecular Networking

Raw LC–MS data were converted to the .mzXML format using MSConvert (ProteoWizard, https://www.proteowizard.sourceforge.net/) and preprocessed using MZMine version 2.53.^27,28^ Parameters used for preprocessing included mass detection: MS1; noise level: 10^5^; MS/MS noise level 10^1^; min time span: 0.01 min; chromatogram builder: min height 2 × 10^5^ *m*/*z*; tolerance 20 ppm; chromatogram deconvolution; *m*/*z* range for MS/MS scan pairing: 1 Da; RT range for MS/MS scan pairing: 0.15 min; isotopic peak grouper *m*/*z* tolerance: 20 ppm; RT tolerance: 0.1; maximum charge: 3; and representative isotope: most intense. Files were exported as .mgf files and were utilized for molecular networking analysis. A molecular network was created using the classic workflow on the GNPS website (http://gnps.ucsd.edu).^29^ The data were filtered by removing all MS/MS fragment ions within ±17 Da of the precursor *m*/*z*. MS/MS spectra were window filtered by choosing only the top 6 fragment ions in the ±50 Da window throughout the spectrum. The precursor ion mass tolerance was set to 0.02 Da and an MS/MS fragment ion tolerance of 0.02 Da. A network was then created where edges were filtered to have a cosine score above 0.55 and more than 5 matched peaks. Further, edges between two nodes were kept in the network if and only if each of the nodes appeared in each other’s respective top 10 most similar nodes. Finally, the maximum size of a molecular family was set to 100, and the lowest scoring edges were removed from molecular families until the molecular family size was below this threshold. The spectra in the network were then searched against the GNPS spectral libraries. The network (https://gnps.ucsd.edu/ProteoSAFe/status.jsp?task=e7f28fb1024c479b88ae7eb17ff6e482) was visualized using Cytoscape 3.6.1 software.^30^ LCMS data were deposited in MassIVE (MSV000099122).

### SIRIUS Annotation

For annotation and dereplication, putative molecular formulas and chemical classes of the prioritized features were determined using SIRIUS 5.8.^31^ SIRIUS was operated with the following parameters: general instrument (Q-ToF), MS/MS mass accuracy (10 ppm), MS/MS isotope scorer (score), candidates stored (20), use DBs in formula only (none), elements allowed in the molecular formula (H, C, N, and O). CSI:FingerID Fingerprint prediction: Fallback adducts (All), Search DBs (All); Tag lipids (off); and CANOPUS (on). In addition to dereplication using CANOPUS, manual searches on Chemspider, SciFinder, Metlin, Dictionary of Natural Products, NPAtlas, CyanoMetDB, and in our *in house* database were performed.^32–36^

## Results and Discussion

### Creating and Screening a Cyanobacterial Fraction Library

In the present study, 19 strains from 5 genera of cyanobacteria previously identified in the Culture Collection of Algae, Cyanobacteria, and Fungi of the Institute of Botany (CCIBt), State of São Paulo were investigated: *Calothrix* (*n* = 5); *Geitlerinema* (*n* = 2); *Leptolyngbya* (*n* = 3); *Nostoc* (*n* = 4); and *Phormidium* (*n* = 5) (Supporting Information Table S1). Figure 1 shows the geographical location of the source collection for each strain. Only six of these strains have been investigated in previous studies. Strains CCIBt3241, CCIBt3248, and CCIBt3280 were investigated for their antioxidant potential and their content of proteins, carbohydrates, and lipids with varied responses.^37^ Strain CCIBt3247 was shown to produce porphyra-334 after exposure to UV light for three days.^38,39^ Strains CCIBt3278 and CCIBt3280 were shown to be active as acetylcholinesterase inhibitors.^40^ CCIBt3324 was investigated for the production of microginins, with negative results.^41^ Strains CCIBt3324 and CCIBt3247 were investigated for their activity against *Artemia salina*, where only CCIBt3324 showed moderate cytotoxicity.^17^

**Figure 1:**
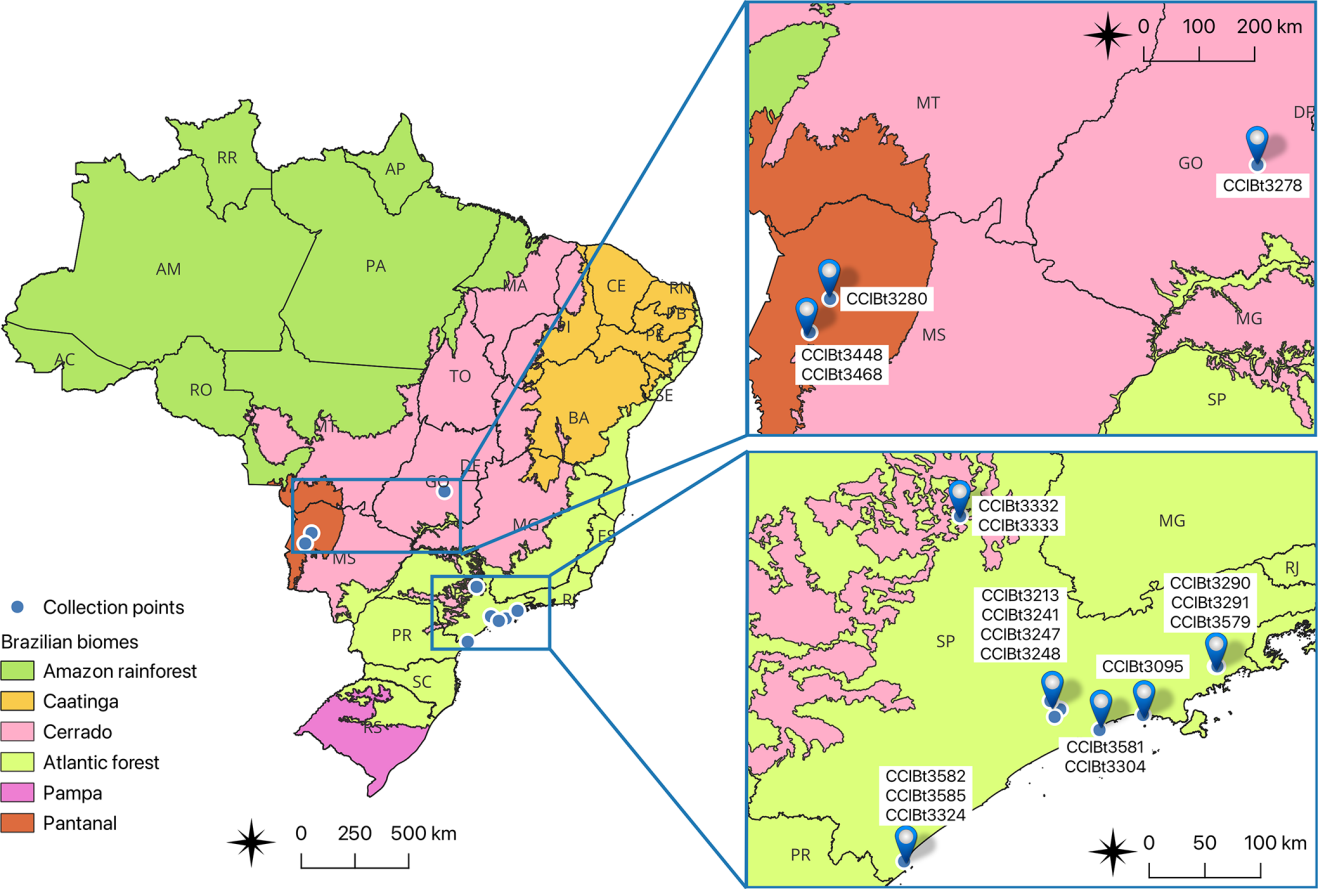
Geographic location of the source collection of the 19 analyzed cyanobacterial strains.

Cyanobacterial strains under investigation were cultured, cells were extracted, and the extracts were subjected to a prefractionation using solid-phase extraction. The creation of fraction libraries standardizes chemoprospecting and allows for an efficient high-throughput screening process for the discovery of new natural products.^42^ Martínez-Fructuoso et al. reported that in approximately 75% of cases, antimicrobial activity was observed in fractions despite the corresponding crude extracts exhibiting no such activity.^43^ This demonstrates the advantages of fractionating crude extracts prior to screening potential bioactive molecules. The reduced chemical complexity in the fractions allows for the identification of compounds that were previously masked by the complex matrix of the crude sample.^42^ The obtained masses of extracts and fractions are shown in Supporting Information Table S2.

Bioassay results are shown in Figure 2 and Supporting Information Table S3. In the cytotoxicity assay, samples shown to be bioactive at 50 μg/mL were retested at 5 μg/mL. Fractions from strains *Phormidium* sp. CCIBt3280 and *Calothrix* sp. CCIBt3582 were bioactive against both HCT-116 and MCF-7 cells at 5 μg/mL, F3 and F4 for CCIBt3582, and F4 and F5 for CCIBt3280. Fractions from strain *Phormidium* sp. CCIBt3278 showed biological activity solely against HCT-116 at 5 μg/mL. The MCF-7 strain proved to be more resistant to treatment with cyanobacteria extracts and fractions.

**Figure 2:**
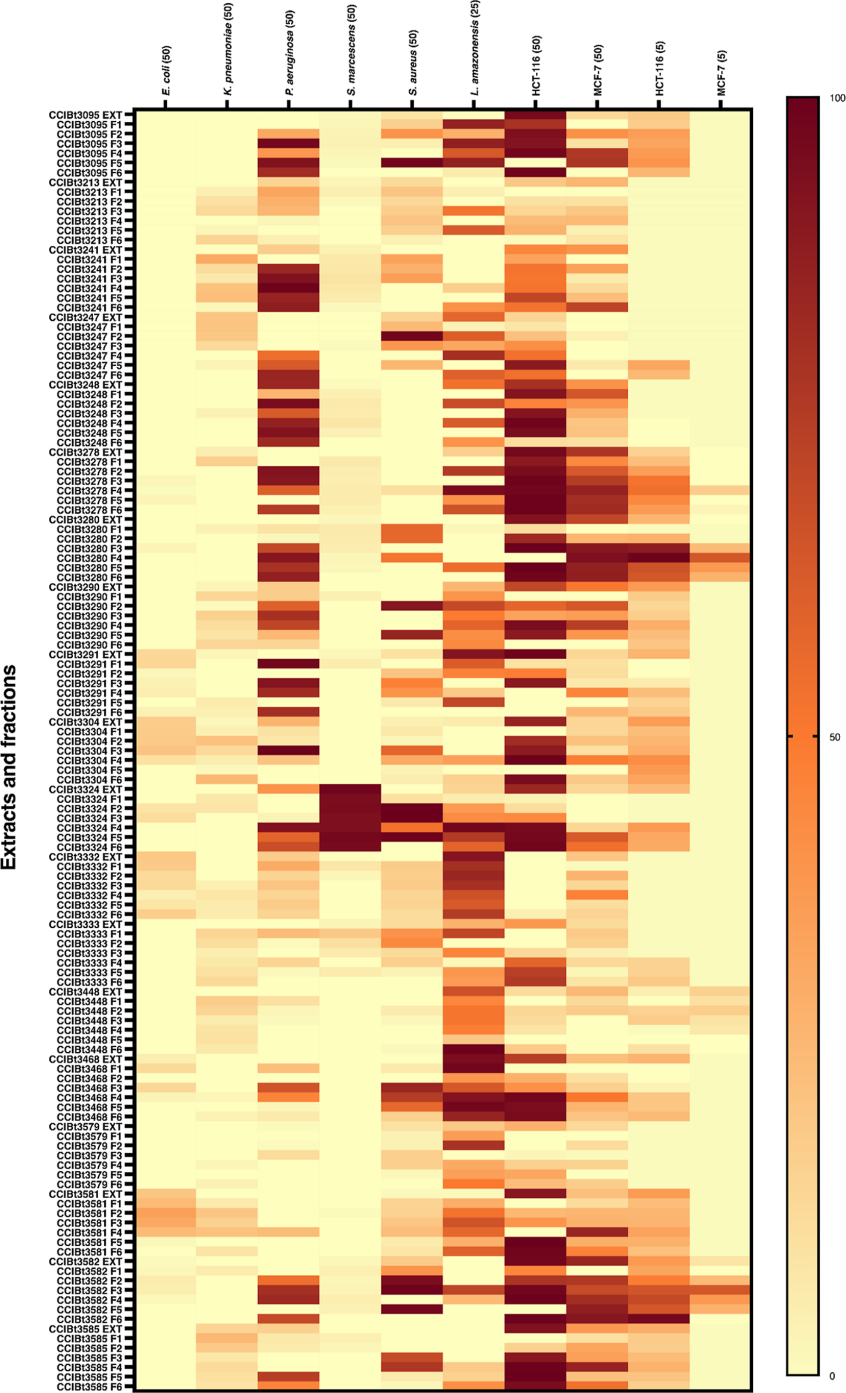
Heatmap showing the percentage of inhibition observed in the bioassays. Values in parentheses indicate the tested concentrations (μg/mL). E. c. = *E. coli*; K.p. = *K. pneumoniae*; P.a. = *P. aeruginosa*; S.m. = *S. marcescens*; S.a. = *S. aureus; L.a.* = *L. amazonensis.* 50 = 50 μg/mL; 25 = 25 μg/mL; 5 = 5 μg/mL.

Antibacterial assays against strains representing bacteria of clinical relevance demonstrated that samples from 12 cyanobacterial strains exhibited inhibitory activity against *P. aeruginosa* (ATCC 27853) at a concentration of 50 μg/mL. Fractions from seven strains displayed antibacterial results against *S. aureus*. Only fractions from strain CCIBt3324 were significantly bioactive against *S. marcescens* (Figure 2, Supporting Information Table S3).

In the antileishmanial assay, strains with fractions that showed inhibitory results against *L. amazonensis* included CCIBt3095, CCIBt3247, CCIBt3278, CCIBt3280, CCIBt3304, and CCIBt3582 (Figure 2, Supporting Information Table S3). None of the crude extracts exhibited activity greater than 70%, reinforcing the importance of fractionation in improving hit rates during bioassay screening.

### Metabolomics for Sample Prioritization and Dereplication for Determining Chemical Novelty

#### NP Analyst Analyses

Normalized bioassay results and LC–MS data from the fractionated extracts were provided to the NP Analyst for compound activity mapping. By integrating metabolomic profiles with bioactivity readouts, NP Analyst predicts which compounds are likely responsible for the observed bioactivities. It calculates two primary metrics: activity score, reflecting phenotypic strength, and cluster score, indicating the compound prevalence in bioactive fractions. Generally, the higher scoring molecules, or features in the MS1 metabolomic data set, are prioritized for isolation. NP Analyst also provides a suite of visualization, comparison, and exploration tools to support the discovery or potential new compounds and aid in the dereplication of known molecules. Notably, potential bioactive features and their corresponding activity and cluster scores can be visualized in a bioactivity network, illustrating both the bioactivity potential and the distribution of the feature in a sample set. MS data related to the crude extracts were not included in this analysis.

The bioactivity network is composed of nodes, squares nodes representing samples while circular nodes representing mass spectral features, connected by edges that denote the feature presence within a sample. Each circle conveys two key indicators: a deeper red color signifies higher predicted bioactivity (activity score), while a larger diameter reflects a higher consistency of the MS feature activity profile (cluster score). Therefore, circles that are both larger and more intensely colored indicate a stronger likelihood that the corresponding *m*/*z* ratio is associated with a specific biological activity based on the calculated cluster and activity scores. The number of bioactive features identified by NP Analyst for fractions obtained from each cyanobacteria strain is summarized in Supporting Information Table S4. For this analysis, activity and cluster score thresholds were set at 0.5 and 2, respectively.

The NP Analyst bioactivity network revealed eight distinct metabolite communities, each representing groups of compounds with similar biological activity profiles (Figure 3). These communities were grouped using the Louvain method, which subdivides large networks into smaller communities based on node interconnectivities.^44^ This graphical visualization proved to be valuable for prioritizing potentially bioactive features. Communities 1 and 2 stood out as the most prolific in the bioactivity results, even when using a concentration of 5 μg/mL and all communities were dereplicated, as detailed in Figures S1–S6 and Table S5.

**Figure 3:**
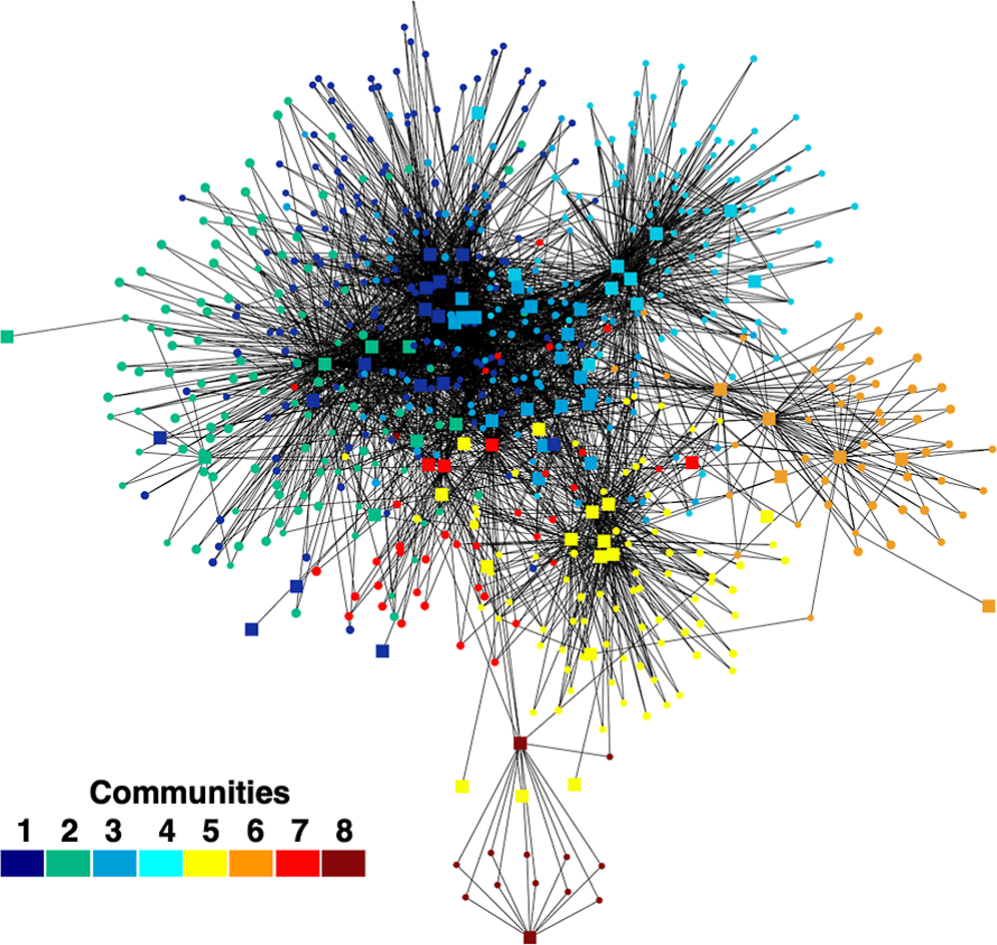
NP Analyst bioactivity network comprising eight communities, derived from LC–MS data of fractions from 19 cyanobacterial strains screened for biological activity. Data were filtered using a minimum activity score of 2 and a cluster score of 0.5.

Community 1 of the bioactivity network includes MS features detected in fractions F1, F2, F3, and F4 from the strain *Calothrix* sp. CCIBt3582 (Figure 4a­(1)). Two features highlighted by NP Analyst, *m*/*z* 1400.7075 [M + H]^+^ at 3.36 min and *m*/*z* 1414.7234 [M + H]^+^ at 3.43 min, were suggested to be linked to antibacterial activity against *S. aureus* and *P. aeruginosa*, as well as cytotoxicity against HCT-116 and MCF7 cell lines, as indicated by high activity (2.26 and 3.31, respectively) and cluster (0.53 and 0.54, respectively) scores (Figure 4a). Using SIRIUS, the molecular formulas calculated for these protonated features are C_63_H_98_N_15_O_21_^+^ and C_64_H_100_N_15_O_21_^+^, respectively. CANOPUS classified these compounds as cyclic depsipeptides, and a thorough search of NP databases such as NP Atlas showed no recorded matches.

**Figure 4:**
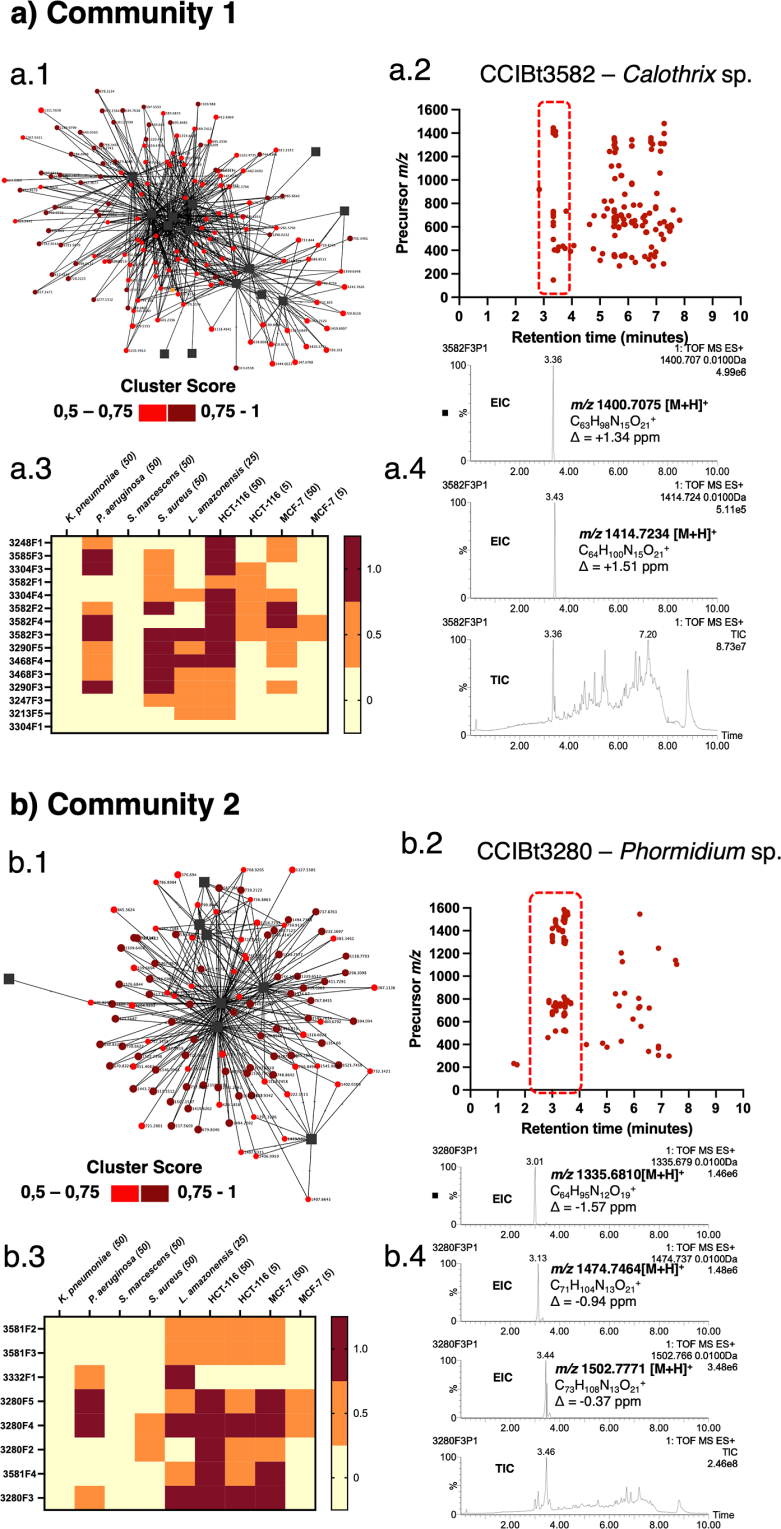
Communities 1 (a(1)) and 2 (b(1)) identified in the NP Analyst bioactivity network derived from fractions of 19 cyanobacterial strains. Panels a(2) and b(2): retention time (*x*-axis) vs *m*/*z* ratio (*y*-axis) plots highlighting the distribution of bioactive features. Panels a(3) and b(3): bioassay readouts showing activity scores associated with each feature. Panels a(4) and b(4): extracted ion chromatograms and corresponding predicted molecular formulas for prioritized features.

Community 2 consists of fractions F2, F3, F4, and F5 from strain *Phormidium* sp. CCIBt3280 (Figure 4(1)). High activity and cluster scores of three features in the NP Analyst network, *m*/*z* 1335.6810 [M + H]^+^, 1474.7450 [M + H]^+^, and 1502.7771 [M + H]^+^, are linked to antibacterial activities against *P. aeruginosa*, *Leishmania*, and HCT-116 and MCF7 cells, as indicated by high activity (4.22, 4.22, and 2.53, respectively) and cluster (0.83, 0.83, and 0.65, respectively) scores (Figure 4). The SIRIUS-calculated molecular formulas for these MS features are C_64_H_95_N_12_O_19_^+^, C_71_H_104_N_13_O_21_^+^, and C_73_H_108_N_13_O_21_^+^, respectively. CANOPUS classified these compounds as potential peptides and revealed a similarity of 63.67% between feature *m*/*z* 1502.7771 [M + H]^+^ and lyngbyazothrin D/portoamide B (calc. *m*/*z* 1502.7777 [M + H]^+^).^45,46^ This structural similarity was further investigated by GNPS molecular networking.

Unlike the NP Analyst bioactivity network, which scores MS1 features based on intensity, mass, and bioactivity patterns, the GNPS molecular network graphically represents spectral similarity from MS/MS spectra.^47^ In the molecular network, nodes represent consensus MS/MS spectra and are connected based on the degree of similarity between compound fragmentation profiles, measured by the cosine score.^47^ While NP Analyst bioactivity networking establishes correlations between MS1 data and bioactivity, GNPS molecular networking assesses the spectral similarity of features and compares them to the GNPS globally curated spectral library to enable the annotation of compounds.

### GNPS Classical Molecular Networking Analyses

The GNPS molecular network contained a total of 11,558 features, with 1137 being exclusive to *Geitlerinema* strains, 1953 to *Nostoc*, 1280 to *Phormidium*, 1670 to group *Calothrix*, and 1095 to *Leptolyngbya*. The other combinations of shared nodes are described in the Venn diagram in Figure 5. The molecular network is shown in Figure S7.

**Figure 5:**
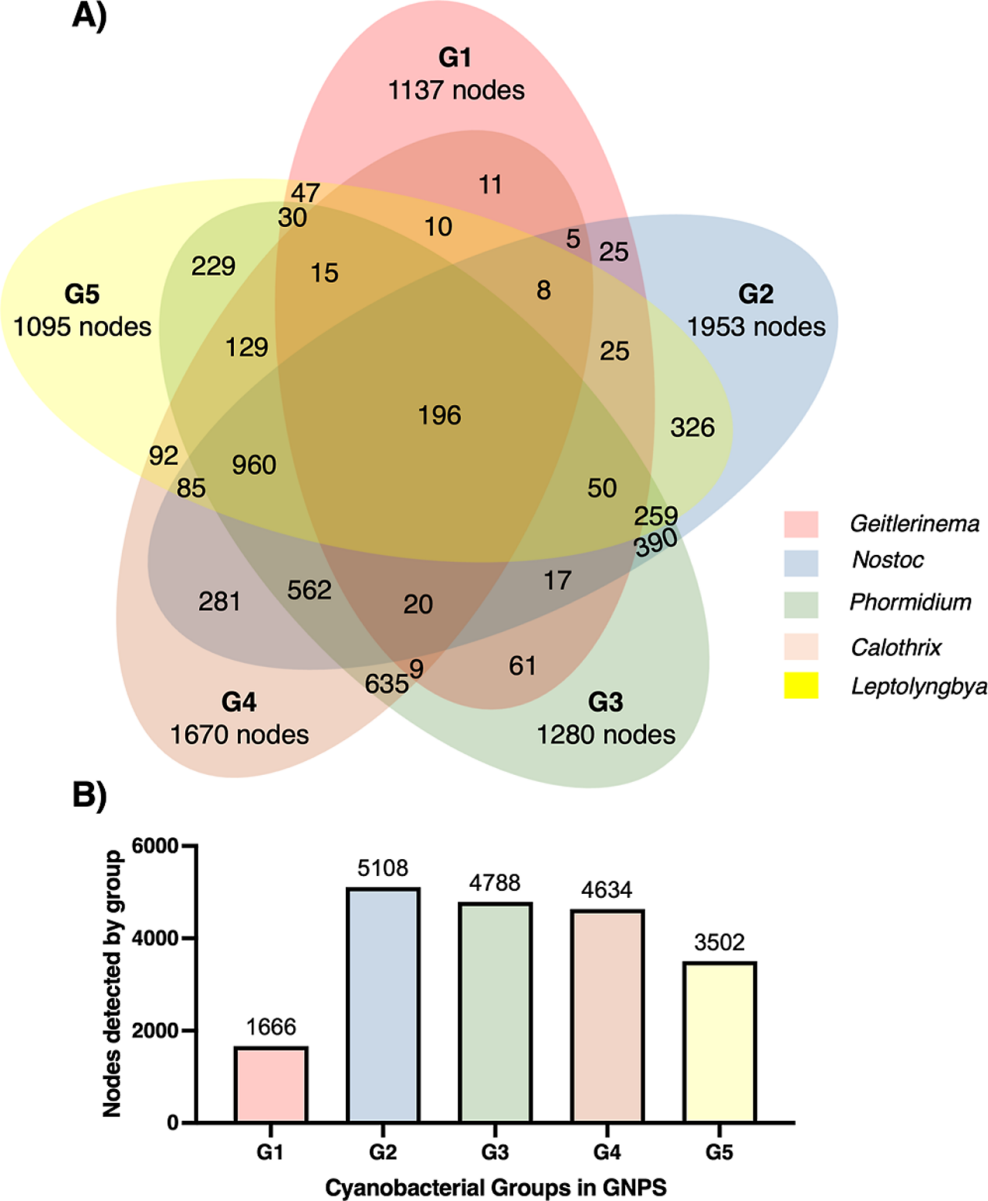
Distribution of the nodes (features) found in the GNPS molecular network by genus. (A) Venn diagram. (B) Total nodes detected in samples by genus.

### Integration of NP Analyst and GNPS Analyses

MS features from all eight NP Analyst communities were detected in the GNPS molecular network analysis (Figures S11–S18). Features from communities 1 and 2 were unique to genera *Calothrix* and *Phormidium*, respectively. Features of communities 3, 7, and 8 were detected across different cyanobacterial genera. Community 4 features showed spectral similarity to those of aeruginosins and anabaenopeptins. Features from communities 5 and 6 were exclusive to genera *Phormidium* and *Leptolyngbya*, respectively. Identifying bioactive compounds within this diverse range of metabolites can be challenging. However, by combining the NP Analyst and GNPS analyses, we refined this search, highlighting the most promising communities and features (Figures S8–S15).

Community 1 was composed of features detected in *Calothrix* sp. CCIBt3582, features with *m*/*z* = 1400.7075 [M + H]^+^ and *m*/*z* = 1414.7234 [M + H]^+^, here described as compounds **1** and **2**, were shown to be in the same cluster, indicating spectral similarity. This cluster also presents features derived from the isotopic pattern ([^13^C_1_M + H]^+^ at *m*/*z* 1401) and the adduct [M + 2H]^2+^ at *m*/*z* 707. None of the dereplication tools recognized these compounds as known.

Community 2 was composed of features detected in *Phormidium* sp. CCIBt3280, the three features (1335.6810 [M + H]^+^, 1474.7450 [M + H]^+^, and 1502.7771 [M + H]^+^), suggested to be linked to the biological activity were grouped in the same cluster and described as compounds **3**, **4**, and **5**. The Dereplicator^48^ tool from the GNPS platform further supported the results previously obtained by SIRIUS. The ion at *m*/*z* 1502.7771 [M + H]^+^ was suggested to have a spectral similarity to lyngbyazothrin, a cyclic undecapeptide from *Lyngbya* sp. with antimicrobial activity. Lyngbyazothrins from *Lyngbya* sp. and portoamides from *Phormidium* sp. are different names given to the same cyclic peptides containing polyketide extensions.^45,46^ Portoamides A–D have the following formulas: C_74_H_109_N_13_O_22_ (A), C_73_H_107_N_13_O_21_ (B), C_62_H_96_N_12_O_19_ (C), and C_61_H_94_N_12_O_18_ (D). A detailed, manual comparison of the fragmentation pattern of the compound at *m*/*z* 1502.7771 [M + H]^+^ with the MS/MS spectrum of portoamide B in GNPS showed insufficient similarity to confirm the annotation. While SIRIUS and Dereplicator suggested a similarity to portoamides/lyngbyazothrins, the differences in fragmentation profiles indicate structural differences. The observed differences in fragmentation patterns suggest underlying structural variations despite an identical molecular formula. These discrepancies may reflect distinct amino acid compositions or alterations in the sequences of the residues.

Community 3 was composed of features detected in multiple strains with the majority of features detected in samples from *Calothrix* sp. CCIBt3585. According to NP Analyst results, features associated with bioactivity eluted at 5.52 and 5.83 min and displayed ions at *m*/*z* 639.2822 [M + Na]^+^, which forms a sodium adduct dimer at *m*/*z* 1299.5262 [2M + Na]^+^, and at *m*/*z* 653.2988 [M + Na]^+^, which forms a sodium adduct dimer at *m*/*z* 1327.5590 [2M + Na]^+^, described as compounds **6** and **7**. The presence of potential isomers in community 3 is evidenced in Figure S1. Analysis using SIRIUS and CANOPUS suggested the molecular formulas C_23_H_40_N_10_O_10_ [M + Na]^+^ and C_40_H_42_N_2_O_5_ [M + Na]^+^ for the compounds **6** and **7**, classifying them as an aminocyclitol glycoside and a tetrapyrrole derivative, with mass errors of Δ +0.15 and Δ +0.45 ppm, respectively.

For community 4, the automatic annotation by GNPS detected the presence of a cluster containing known and two potentially new aeruginosins that were dereplicated and will be discussed in the next section, described here as compounds **8** and **9**. The known aeruginosins are described in Table S5.

Community 5 was composed of features detected in strain *Phormidium* sp. CCIBt3095, NP Analyst highlighted the features *m*/*z* 936.6158 [M + H]^+^, 966.6272 [M + H]^+^, and 950.6320 [M + H]^+^, described as compounds **10**, **11**, and **12**, as the most correlated with bioactivity, eluting between 3.29, 3.62, and 3.53 min, respectively. CANOPUS classified them as cyclic depsipeptides, with molecular formula C_50_H_85_N_3_O_13_^+^ (Δ +0.29 ppm) for compound **10**, C_52_H_83_N_7_O_10_^+^ (Δ −0.20 ppm) for compound **11**, and C_47_H_83_N_9_O_11_^+^ (Δ +3.78 ppm) for compound **12**, respectively. In the GNPS molecular network, these features formed strain-exclusive clusters. No dereplication tool identified these compounds, suggesting a potential chemical novelty.

In community 6, containing features detected in samples from *Leptolyngbya* sp. CCIBt3324, NP Analyst highlighted three features of interest with *m*/*z* values of 886.5874 [M + H]^+^, 1032.6455 [M + H]^+^, and 870.5933 [M + H]^+^, described as compounds **13**, **14**, and **15**, eluting at 4.97, 5.09, and 5.23 min, respectively. In GNPS molecular networking, these features were grouped into distinct clusters. SIRIUS predicted the molecular formulas C_49_H_75_N_9_O_6_^+^ for compound **13** (Δ −4.39 ppm), C_54_H_89_N_5_O_14_^+^ for compound **14** (Δ −2.22 ppm), and C_43_H_79_N_7_O_11_^+^ (Δ +2.64 ppm) for compound **15**. CANOPUS classified compounds **14** and **15** as cyclic depsipeptides, while compound **13** was categorized as a derivative of α-amino acid. Dereplication of these bioactive molecules also failed to find matches with the previously described compounds.

Community 7 was composed of features detected in samples from different strains of cyanobacteria. The feature with *m*/*z* 511.2919 [M + Na]^+^ was shared between *Calothrix* sp. (CCIBt3581 and CCIBt3582) and *Phormidium* sp. (CCIBt3095 and CCIBt3278). The feature *m*/*z* 539.3193 [M + Na]^+^ was shared between *Phormidium* sp. (CCIBt3095, CCIBt3278, and CCIBt3280), *Calothrix* sp. (CCIBt3304, CCIBt3581, and CCIBt3582), *Nostoc* sp. (CCIBt3248), and *Leptolyngbya* sp. (CCIBt3324). The feature with *m*/*z* 595.2485 [M + Na]^+^ was shared between *Calothrix* sp. (CCIBt3304, CCIBt3581, CCIBt3582, and CCIBt3585), *Leptolyngbya* sp. (CCIBt3324), *Nostoc* sp. (CCIBt3248), and *Phormidium* sp. (CCIBt3095, CCIBt3278, and CCIBt3280), according to the GNPS cluster. Features with *m*/*z* values of 511.2919 [M + Na]^+^, 539.3193 [M + Na]^+^, and 595.2485 [M + Na]+ are here described as compounds **16**, **17**, and **18**. The following molecular formulas were calculated: C_31_H_40_N_2_O_3_^+^ (Δ −2.34 ppm) for compound **16**, C_24_H_40_N_10_O_3_^+^ (Δ +2.96 ppm) for compound **17**, and C_20_H_44_N_8_O_5_S_3_ (Δ −0.67 ppm) for compound **18**. CANOPUS classified compound **17** as an amino acid and derivatives. There were no matches for compounds **16** and **18**. Since these features were detected in distinct cyanobacteria, we hypothesize that they represent primary metabolites.

Community 8 includes features detected in samples from various genera, with three features of interest related to observed bioactivities: *m*/*z* 791.5274 [M + Na]^+^, detected in CCIBt3248, CCIBt3247, CCIBt3291, CCIBt3333, CCIBt3468, and CCIBt3579, *m*/*z* 805.5052 [M + Na]^+^, detected in CCIBt3579 and CCIBt3248, and *m*/*z* 809.5359 [M + Na]^+^, only in CCIBt3248, eluting at 7.41, 6.46, and 7.05 min, respectively. Here, features are described as compounds **19**, **20**, and **21**. Analysis using SIRIUS resulted in the following molecular formulas: C_43_H_76_O_11_^+^ (Δ −0.63 ppm) for compound **19**, C_39_H_70_N_6_O_10_^+^ (Δ +0.86 ppm) for compound **20**, and C_38_H_78_N_2_O_14_^+^ (Δ of +1.72 ppm) for compound **21**, classified as fatty acid, peptide, and an alkyl glycoside, respectively. Our data suggested the existence of isomers for these compounds. For the compound with *m*/*z* 805.5052 [M + Na]^+^, an isomer eluting at 7.13 min was identified. For *m*/*z* 791.5274 [M + Na]^+^, four isomers were identified, and for *m*/*z* 809.5359 [M + Na]^+^, two isomers were also observed (Figure S6). None of the features was previously reported by any of the dereplication tools used. Figure 6 illustrates the selection of features using the NP Analyst bioactivity network and the GNPS molecular network. Table S6 and Figure 6 summarize the annotations of compounds predicted as bioactive by the NP Analyst and dereplicated using SIRIUS and GNPS.

**Figure 6:**
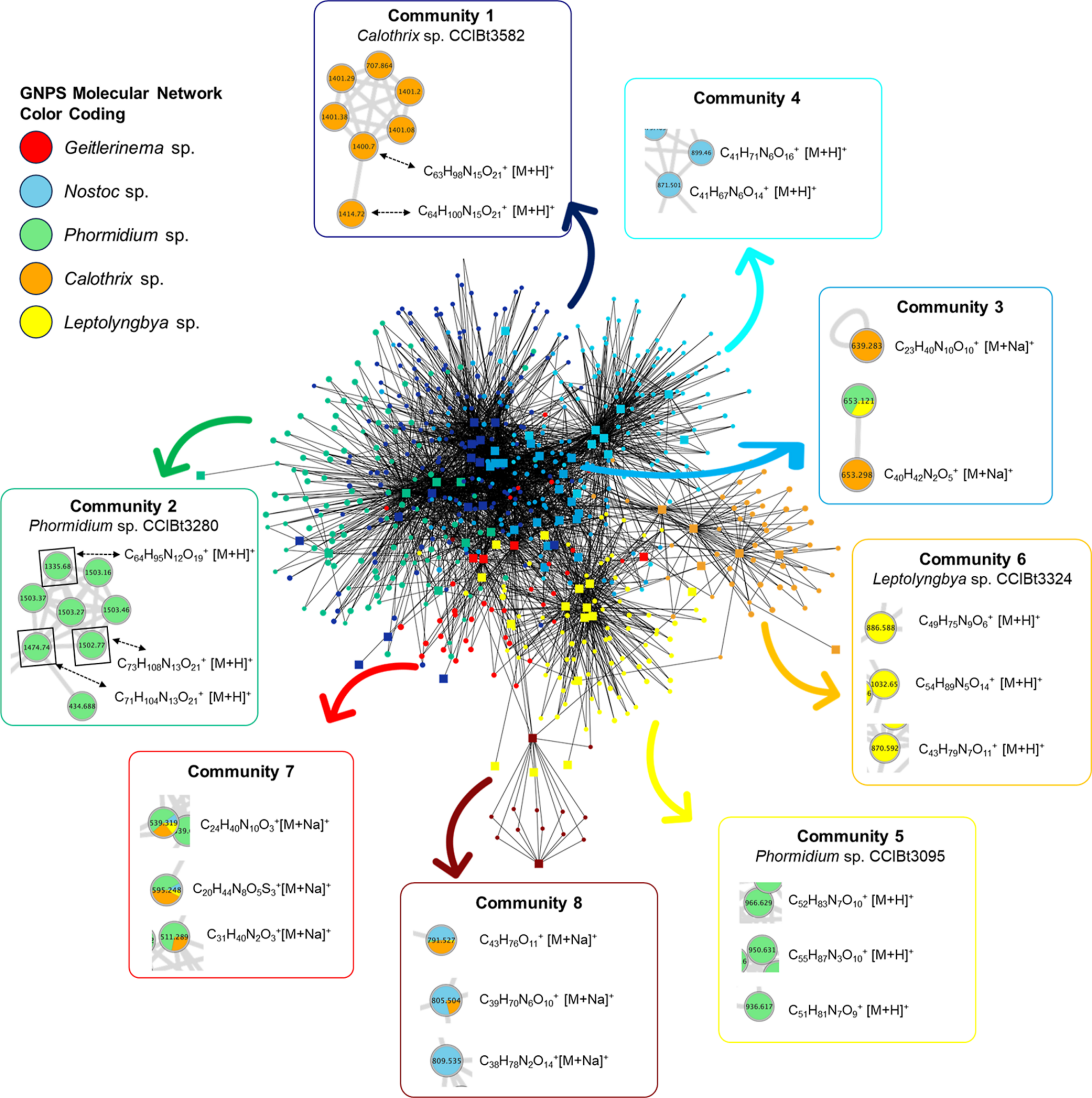
“Finding the needle in the haystack”—prioritization of bioactive features through the integration of the bioactivity-guided fractionation and metabolomic tools NP Analyst, GNPS, and SIRIUS.

Interestingly, the GNPS platform also identified 672 features in our molecular network that exhibited spectral similarity to entries in its spectral library. The annotation and dereplication of selected secondary metabolites are discussed below.

### Annotation of Compounds in Analyzed Extracts and Fractions

Anabaenopeptins are cyclic peptides typically comprising six amino acids that have been identified in cyanobacteria from the genera *Anabaena*, *Nostoc*, *Microcystis*, *Planktothrix*, *Lyngbya*, and *Brasilonema*.^49^ These peptides inhibit carboxypeptidases, phosphatases, and serine proteases.^50^ While Zervou et al.^51^ did not find anabaenopeptins in the genus *Calothrix*, their presence can now be reported through GNPS annotation and comparison with authentic standards. Detailed analysis of spectral data for compound with *m*/*z* 837.4617 [M + H]^+^ supported its dereplication as anabaenopeptin B. The compound with *m*/*z* 851.4770 [M + H]^+^ was dereplicated as anabaenopeptin E. Anabaenopeptins E and F share the same formula but differ in their substituent groups. Dereplication was complicated by the isomeric nature of anabaenopeptin E, which has four structures anabaenopeptins B1, F, MM850, and DA850.^52^ Fragments at *m*/*z* 202 and *m*/*z* 175 aided in identifying anabaenopeptins E and F, while the fragment at *m*/*z* 201.0963, corresponding to arginine, was crucial in excluding other isomers.^53^ The analysis followed the fragmentation route proposed by Sieber et al.,^54^ confirming the putative presence of anabaenopeptin F/DA850 in the cyanobacterium *Calothrix* sp. CCIBt3581. The absence of a peak corresponding to a methyl substituent group ruled out the possibility of it being anabaenopeptin E. This finding underscores the potential for further studies with *Calothrix*. Although *Calothrix* sp. CCIBt3581 did not show biological activity, the presence of the compound suggests potential protease inhibitory effects. The prefraction from strain CCIBt3581, which contained anabaenopeptins B and F/DA850, was reinjected into a HPLC–UV–MS system along with authentic standards of anabaenopeptins B and F, previously isolated by Harms,^55^ to confirm their presence based on retention time and MS data comparison. Individual runs were performed for both the standards and the fraction, followed by spiking the standards into fraction 2 (which contained the anabaenopeptins). The coelution of compounds at identical retention times confirmed their identity, with anabaenopeptin B eluting at 11.9 min and anabaenopeptin F at 12.3 min, the latter showing higher abundance compared to the former. The MS1 spectra from the analyses with the standards are shown in Figure S16.

Thus, the presence of these compounds was confirmed, enabling the establishment of a chemotaxonomic novelty regarding the production of these compounds within the genus *Calothrix*. The MS and MS/MS spectra of the anabaenopeptins are shown in Figures S17 and S18.

Also detected were samples from *Calothrix* sp. CCIBt3581, featuring *m*/*z* values of 794.4448 [M + H]^+^ and 808.4609 [M + H]^+^, which composed a separate cluster in the molecular network consistent with anabaenopeptins (MS spectra in Figures S19 and S20). Dereplication using the SIRIUS indicated a 66% probability that the compound with *m*/*z* 794.4448 [M + H]^+^ corresponds to anabaenopeptin J^56^ (molecular formula C_41_H_59_N_7_O_9_^+^, Δ +0.12 ppm) and the molecular formula of the compound with *m*/*z* 808.4609 [M + H]^+^ was suggested to be the same as anabaenopeptin 807 ^57^ (molecular formula C_42_H_61_N_7_O_9_^+^, Δ +0.74 ppm). Of note, SIRIUS was unable to detect the spectral similarity of compound *m*/*z* 808.4609 [M + H]^+^ with anabaenopeptins, even though GNPS grouped its ion within the same cluster as other anabaenopeptins. This demonstrates that dereplication using multiple tools can be essential in the compound annotation process.

In the molecular network, the compounds with *m*/*z* 794.4448 [M + H]^+^ and 808.4609 [M + H]^+^, detected in strain CCIBt3581, clustered together with the ions at *m*/*z* 842.4460 [M + H]^+^ and 858.4380 [M + H]^+^, detected in samples from strain *Phormidium* sp. CCIBt3280. The *m*/*z* value of 842.4460 [M + H]^+^ corresponds to the protonated ion of six previously described anabaenopeptins: 841A, 842B, 842, NZ841, anabaenopeptin KVJ841, and lyngbyaureidamide B.^58–63^ SIRIUS calculated a 51.7% probability that this compound is lyngbyaureidamide B (molecular formula C_45_H_59_N_7_O_9_^+^, Δ +1.54 ppm). The ion with an *m*/*z* value of 858.4380 [M + H]^+^ corresponds to the mass of anabaenopeptins 857, NZ857, and oscillamide Y. SIRIUS indicated a 72.3% probability that this compound is oscillamide Y (molecular formula C_45_H_59_N_7_O_10_^+^ [M + H]^+^, Δ −1.86 ppm) (MS spectra in Figures S21 and S22).^59,61,64^ Previous studies have already demonstrated the presence of the hphABCD gene, part of the gene cluster responsible for anabaenopeptin biosynthesis in cyanobacteria of the genus *Phormidium*, supporting the findings observed in this study.^65^

For *Nostoc* sp. CCIBt3291, SIRIUS identified the compound with an *m*/*z* of 842.4448 [M + H]^+^ as anabaenopeptin NZ841 ^61^ with 57.5% of similarity (molecular formula C_45_H_59_N_7_O_9_^+^, Δ +0.11 ppm). Conversely, the compound with an *m*/*z* 858.4387 [M + H]^+^ was classified as also structurally closer to oscillamide Y,^64^ exhibiting 65.6% similarity (C_45_H_59_N_7_O_10_^+^, Δ −1.04 ppm). For the compound with *m*/*z* 856.4575 [M + H]^+^, the significant error in ppm (+35.1) suggests that it does not correspond to any of the anabaenopeptin with *m*/*z* 856 [M + H]^+^ listed in the CyanoMetDB database, which includes nodulapeptides 855 A and B. SIRIUS analysis indicated the molecular formula C_46_H_62_N_7_O_9_^+^ (Δ −3.38 ppm) as the most probable for this compound and 51.8% of correlation with lyngbyaureidamide A.^63^ Furthermore, the presence of this compound in the same cluster as the putative anabaenopeptins points to a potential structural similarity, suggesting that it may represent this anabaenopeptin (MS spectra in Figures S23–S25).

Aeruginosins are nonribosomal linear tetrapeptides characterized by the presence of the 2-carboxy-6-hydroxy-octahydroindole (Choi) structure at the central position, whose characteristic fragment displays an *m*/*z* of 140.1070. Over 130 aeruginosin variants have been described in the literature, bioactive peptides that inhibit trypsin. These compounds have been reported in cyanobacteria from the genera *Microcystis*, *Planktothrix*, *Nostoc*, and *Nodularia*, as well as in some marine sponges.^66^

All nodes in the aeruginosin cluster were dereplicated using data from CyanoMetDB, NP Atlas, and SIRIUS software (Figure 7). By comparing spectra data for known aeruginosins described in the literature, we can compare mass to spectrometry results for samples from *Nostoc* sp. CCIBt3291, we tentatively identified ten known aeruginosins: aeruginosin 736, 752, 766, 822, 836, 848, 850, 865, 878a, and 892. All aeruginosins observed in the present study, except for aeruginosin 865, were initially described by Sanz et al.,^59^ and their MS/MS fragmentation profiles are consistent with literature data (Figures S21–S29). Among the detected aeruginosins, the peak corresponding to aeruginosin 864 showed the highest intensity, with the fragments detected in the MS/MS spectrum consistent with those reported by Kapuścik et al.^67^ Building upon previous work, here we propose fragment ion annotations for the detected aeruginosins (Figures S26–35). Almost all dereplicated aeruginosins showed that the Choi group reduced to its immonium ion, with an *m*/*z* ratio of 138.0913, except for aeruginosin 736, which exhibited the protonated Choi group with an *m*/*z* ratio of 140.1070. Although the ion at *m*/*z* 849.4601 matched the molecular formula of aeruginosin 848, its identification could not be confirmed due to the low intensity of the fragment ions.

**Figure 7:**
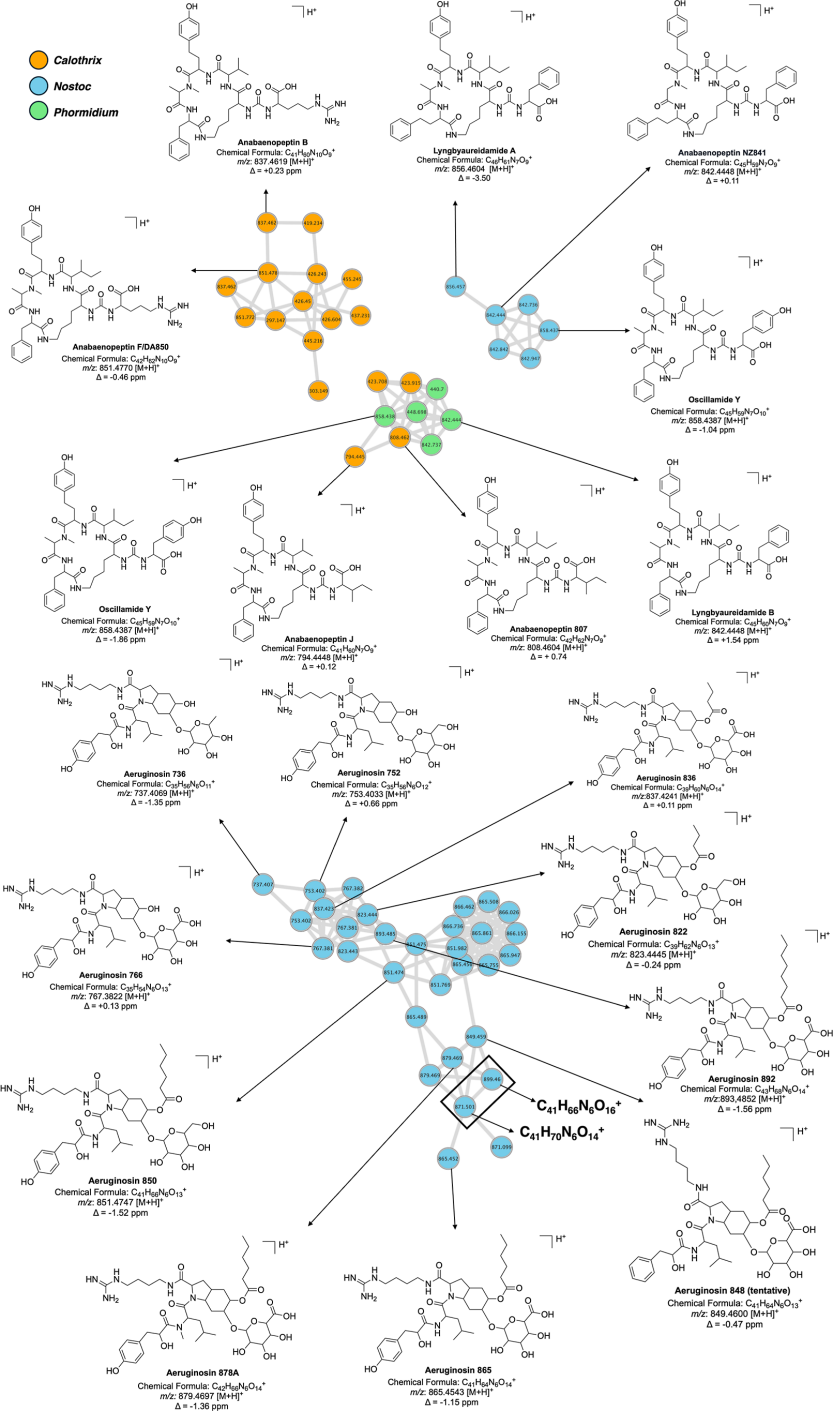
Validated, putative, and tentative annotations of anabaenopeptins and aeruginosins investigated via GNPS, followed by manual dereplication using SIRIUS, Dereplicator, Dereplicator^+^, and extensive database searches, which revealed potentially new aeruginosins with *m*/*z* 871.5018 [M + H]^+^ and 899.4602 [M + H]^+^.

Detailed dereplication and literature searches did not yield known compounds for features with *m*/*z* ratios of 871.5018­[M + H]^+^ and 899.4602 [M + H]^+^ detected in the aeruginosin cluster. Thus, these two potential new variants of aeruginosins were putative identified. Compound **8** has a predicted molecular formula of C_41_H_70_N_6_O_14_^+^ (Δ −0.45 ppm), classified as the hybrid peptide, while compound **9** is predicted to have a molecular formula of C_41_H_66_N_6_O_16_^+^ (Δ −0.66 ppm) classified as oligopeptide (MS and MS/MS spectra in Figures S36 and S37). These features will be verified through isolation and structural elucidation, aiming to confirm the new structural variants of aeruginosins.

This analysis enabled the identification of secondary metabolites from cyanobacteria using different metabolomic annotation strategies.^68,69^ Anabaenopeptins B and F from *Calothrix* sp. (CCIBt3581) were confirmed through comparison with authentic standards (level 1 annotation). In contrast, most anabaenopeptins from *Phormidium* sp. (CCIBt3280) and aeruginosins from *Nostoc* sp. (CCIBt3291) were putatively identified (level 2), except for aeruginosin 848, which reached only level 3. For putative new aeruginosins, MS/MS data allowed for structural suggestions, classifying them under a level 2 annotation. Figure 7 highlights the annotated compounds.

The analysis of community 4 in NP Analyst highlighted that anabaenopeptins and aeruginosins were the possible compounds responsible for the observed bioactivities against *P. aeruginosa*, *L. amazonensis*, and HCT-116, exhibiting varied activity and cluster score values. By integrating fractionation strategies with metabolomic tools such as NP Analyst, SIRIUS, and GNPS, we successfully identified 20 features potentially linked to the observed bioactivities. This approach enhanced the efficiency of compound selection and prioritization, streamlining the identification of bioactive metabolites. Various other prioritization strategies for natural products are reported in the literature. For instance, Olivon et al.^70^ demonstrated the correlation between bioactivity data and taxonomic information through molecular network mapping. Another promising approach involves tools that integrate bioactivity, mass spectrometry (MS), and nuclear magnetic resonance data, such as DAFdiscovery.^16^ This platform also utilizes bioactivity, spectroscopic, and spectrometric data to identify patterns in chemical and biological data sets, facilitating the discovery of novel bioactive natural products with therapeutic potential. Thus, our results present a functional and integrated strategy that combines different techniques for the prioritization of the natural products of interest. Additionally, we highlight the chemical and bioactive richness of cyanobacteria collected in Brazil, emphasizing their biotechnological potential in the search for new antibacterial, cytotoxic, and other bioactive compounds of interest by a combination of metabolomic tools that enabled the prioritization of features based on both MS1 data and bioactivity, as well as MS/MS spectral comparisons. This integrated approach enhanced the selection of features of interest in Brazilian cyanobacteria.

## Conclusion

Here, we demonstrate how integrating metabolomic tools, such as NP Analyst, GNPS, and dereplication with SIRIUS, with the exploration of understudied cyanobacteria taxonomic groups occurring in Brazil can significantly contribute to the discovery of novel bioactive compounds. Our findings highlight the metabolic potential of understudied cyanobacterial strains, highlighting key molecular features correlated with the antibacterial, cytotoxic, and antileishmanial activities observed. By applying a strategic prioritization approach, we identified key features of interest that will advance to the subsequent stages of isolation, structural elucidation, and bioactivity validation.

## Supplementary Material



## Notes

Strain information, fractionation results, bioassay results, and relevant LC–MS and metabolomics data (PDF)
