# Supplementary material for: Chemodiversity of Cyanobacteria from Brazil Investigated by Metabolomics and Bioassays
Source: ACS Omega. 2025 Oct 24;10(43):51569–83. doi: 10.1021/acsomega.5c07322 (PMC12593024; doi:10.1021/acsomega.5c07322)

Supporting information

Chemodiversity of Cyanobacteria from Brazil Investigated by Metabolomics and Bioassays

*Francisco H. S. da Silva, Leonardo S. de Jesus, Michael J. J. Recchia, Kleyton J. G. de Morais, Camila M. C. Gonçalves, Sandra R. C. Soares, Helori V. Domingos, Hannah Cavanagh, Frederico J. G. Filho, José A. L. Lindoso, Leticia V. Costa-Lotufo, Roger G. Linington, Roberto G. S. Berlinck, Camila M. Crnkovic**

Corresponding Author

***Camila M. Crnkovic** - Faculdade de Ciências Farmacêuticas, Universidade de São Paulo, 05508-000 São Paulo, Brazil; orcid.org/0000-0001-6581-9031

E-mail: camilavic@usp.br

Table of contents

Table S1. Source and cultivation parameters of the cyanobacterial strains evaluated in the present study 5
Table S2. Extract and fraction mass obtained from each cyanobacterial strain. 5
Table S3. Percentage of microbial inhibition for cyanobacterial extracts and fractions at 50 µg/mL. E.c. = *E. coli*, K.p.= *K. pneumoniae*, P.a. = *Pseudomonas aeruginosa*, S.m= *S. marcescens*, S.a.= *S. aureus*, SD= Standard deviation. SEM = Standard Error of the Mean....................6
Table S4. Bioactive features identified by NP Analyst for each cyanobacteria strain analyzed 11
Figure S1. Prioritized features of cyanobacteria *Calothrix* sp. CCIBt3585 through NP Analyst and SIRIUS analysis. TIC: Total Ion Chromatogram, EIC: Extracted Ion Chromatogram. A) Community highlighted by NP Analyst. B) Scatter plot of features of interest. C) Heatmap of biological activities of the community. D) Chromatograms, molecular formulas, and error (ppm)....................12
Figure S2. Prioritized features of cyanobacteria *Nostoc* sp. CCIBt3291 through NP Analyst and SIRIUS analysis. TIC: Total Ion Chromatogram, EIC: Extracted Ion Chromatogram. A) Community highlighted by NP Analyst. B) Scatter plot of features of interest. C) Heatmap of biological activities of the community. D) Chromatograms, molecular formulas, and error (ppm)....................13
Figure S3. Prioritized features of cyanobacteria *Phormidium* sp. CCIBt3095 through NP Analyst and SIRIUS analysis. TIC: Total Ion Chromatogram, EIC: Extracted Ion Chromatogram. A) Community highlighted by NP Analyst. B) Scatter plot of features of interest. C) Heatmap of biological activities of the community. D) Chromatograms, molecular formulas, and error (ppm)....................14
Figure S4. Prioritized features of cyanobacteria *Leptolyngbya* sp. CCIBt3324 through NP Analyst and SIRIUS analysis. TIC: Total Ion Chromatogram, EIC: Extracted Ion Chromatogram. A) Community highlighted by NP Analyst. B) Scatter plot of features of interest. C) Heatmap of biological activities of the community. D) Chromatograms, molecular formulas, and error (ppm)....................15
Figure S5. Prioritized features of cyanobacteria *Phormidium* sp. CCIBt3278 through NP Analyst and SIRIUS analysis. TIC: Total Ion Chromatogram, EIC: Extracted Ion Chromatogram. A) Community highlighted by NP Analyst. B) Scatter plot of features of interest. C) Heatmap of biological activities of the community. D) Chromatograms, molecular formulas, and error (ppm)....................16
Figure S6. Prioritized features of cyanobacteria *Nostoc* sp. CCIBt3248 through NP Analyst and SIRIUS analysis. TIC: Total Ion Chromatogram, EIC: Extracted Ion Chromatogram. A) Community highlighted by NP Analyst. B) Scatter plot of features of interest. C) Heatmap of biological activities of the community. D) Chromatograms, molecular formulas, and error (ppm)....................17
Figure S7. Molecular network of all cyanobacterial fractions analyzed using GNPS, where G1 (red): *Geitlerinema* sp., G2 (blue): *Nostoc* sp., G3 (green): *Phormidium* sp., G4 (orange): *Calothrix* sp., G5 (yellow): *Leptolyngbya* sp. 18
Figure S8. A) GNPS cluster of features from the cyanobacterium *Calothrix* sp. CCIBt3582 highlighting the compounds of interest indicated by NP Analyst as associated with the bioactivity. B) Total ion chromatogram (UHPLC-MS). C) MS1 spectra of the compounds of interest for community 1 indicated by NP. 19

Figure S9. A) GNPS cluster of features from the cyanobacterium *Phormidium* sp. CCIBt3280 highlighting the compounds of interest indicated by NP Analyst as associated with the bioactivity. B) Total ion chromatogram (UHPLC-MS). C) MS1 spectra of the compounds of interest for community 2 indicated by NP Analyst. 20
Figure S10. A) GNPS cluster of features from the cyanobacterium *Calothrix* sp. CCIBt3585 highlighting the compounds of interest indicated by NP Analyst as associated with the bioactivity. B) Total ion chromatogram (UHPLC-MS). C) MS1 spectra of the compounds of interest for community 3 indicated by NP Analyst. 21
Figure S11. A) GNPS cluster of features from the cyanobacterium *Nostoc* sp. CCIBt3291 highlighting the compounds of interest indicated by NP Analyst as associated with the bioactivity. B) Total ion chromatogram (UHPLC-MS). C) MS1 spectra of one of the compounds of interest from community 4, identified by NP Analyst 22
Table S5. Annotation and dereplication of the detected aeruginosins..........................22
Figure S12. A) GNPS cluster of features from the cyanobacterium *Phormidium* sp. CCIBt3095 highlighting the compounds of interest indicated by NP Analyst as associated with the bioactivity. B) Total ion chromatogram (UHPLC-MS). C) MS1 spectra of the compounds of interest from community 5, identified by NP Analyst 23
Figure S13. A) GNPS cluster of features from the cyanobacterium *Leptolyngbya* sp. CCIBt3324 highlighting the compounds of interest indicated by NP Analyst as associated with the bioactivity. B) Total ion chromatogram (UHPLC-MS). C) MS1 spectra of the compounds of interest from community 6, identified by NP Analyst...........................24
Figure S14. A) GNPS cluster of features from the cyanobacterium *Phormidium* sp. CCIBt3278 highlighting the compounds of interest indicated by NP Analyst as associated with the bioactivity. B) Total ion chromatogram (UHPLC-MS). C) MS1 spectra of the compounds of interest from community 7, identified by NP Analyst 25
Figure S15. A) GNPS cluster of features from the cyanobacterium *Nostoc* sp. CCIBt3248 highlighting the compounds of interest indicated by NP Analyst as associated with the bioactivity. B) Total ion chromatogram (UHPLC-MS). C) MS1 spectra of the compounds of interest from community 8, identified by NP Analyst 26
Table S6. Annotation of the features highlighted by NP Analyst and dereplicated by SIRIUS...27
Figure S16. Comparison between anabaenopeptin B and F standards and fraction F2 from *Calothrix* sp. CCIBt3581. A = Analysis of anabaenopeptin standards by HPLC-UV-MS. A.1 = Anabaenopeptin B standard. A.2 = Anabaenopeptin F standard. B = Spiking of anabaenopeptin standards into fraction 2. B.1 = Anabaenopeptin B + standard. B.2 = Anabaenopeptin F + standard. C = Fraction 2 detecting anabaenopeptins B and F. C.1 = Anabaenopeptin B. C.2 = Anabaenopeptin F. D = Total ion chromatogram of fraction 2 analyzed by UHPLC-MS/MS. D.1 = Peak corresponding to anabaenopeptin B. D.2 = Peak corresponding to anabaenopeptin F 28
Figure S17. MS data of anabaenopeptin B. (A) MS1 spectrum. (B) MS/MS spectrum with annotated fragment ions 29
Figure S18. MS data of anabaenopeptin F. (A) MS1 spectrum. (B) MS/MS spectrum with annotated fragment ions 30
Figure S19. MS data of anabaenopeptin J and SIRIUS annotation. A) MS1 spectrum. B) MS/MS spectrum. C) SIRIUS annotation 31
Figure S20. MS data of anabaenopeptin 807 and SIRIUS annotation. A) MS1 spectrum. B) MS/MS spectrum. 32
Figure S21. MS data of lyngbyaureidamide B and SIRIUS annotation. A) MS1 spectrum. B) MS/MS spectrum. C) SIRIUS annotation. 33

Figure S22. MS data of oscillamide Y and SIRIUS annotation. A) MS1 spectrum. B) MS/MS spectrum. C) SIRIUS annotation. 34
Figure S23. MS data of anabaenopeptin NZ841 and SIRIUS annotation. A) MS1 spectrum. B) MS/MS spectrum. C) SIRIUS annotation. 35
FigureS24. MS data of oscillamide Y and SIRIUS annotation. A) MS1 spectrum. B) MS/MS spectrum. C) SIRIUS annotation. 36
Figure S25. MS data of lyngbyaureidamide A and SIRIUS annotation. A) MS1 spectrum. B) MS/MS spectrum. C) SIRIUS annotation....................37
Figure S26. MS/MS spectrum and fragment ion annotation of aeruginosin 736 38
Figure S27. MS/MS spectrum and fragment ion annotation of aeruginosin 752 39
Figure S28. MS/MS spectrum and fragment ion annotation of aeruginosin 766 40
Figure S29. MS/MS spectrum and fragment ion annotation of aeruginosin 822 41
Figure S30. MS/MS spectrum and fragment ion annotation of aeruginosin 836 42
Figure S31. MS/MS spectrum and fragment ion annotation of aeruginosin 848 43
Figure S32. MS/MS spectrum and fragment ion annotation of aeruginosin 850 44
Figure S33. MS/MS spectrum and fragment ion annotation of aeruginosin 865 45
Figure S34. MS/MS spectrum and fragment ion annotation of aeruginosin 878A 46
Figure S35. MS/MS spectrum and ion annotation ofthe aeruginosin 892....................47
Figure S36. MS and MS/MS data of the potential novel aeruginosin with *m/z* 871.5018 $[M+H]^+$ 48
Figure S37. MS and MS/MS data of the potential novel aeruginosin with *m/z* 899.4602 $[M+H]^+$ 49

Table S1. Source and cultivation parameters of the cyanobacterial strains evaluated in the present study

Code	Strain	Geographical Origin	Biome	Growing medium
CCIBt3095	*Phormidium* sp.	23°45'57.865"S,45°47'46.083"W	Atlantic Forest	BG-11
CCIBt3213	*G. unigranulatum*	23°43′S e 46°32′W	Atlantic Forest	BG-11
CCIBt3241	*Geitlerinema* sp.	23°47'S, 46°35'W	Atlantic Forest	BG-11
CCIBt3247	*Nostoc* sp.	23°38'30"S, 46°37'14"W	Atlantic Forest	BG-11
CCIBt3248	*Nostoc* sp.	23°38'30"S, 46°37'14"W	Atlantic Forest	BG-11
CCIBt3278	*Phormidium* sp.	16°39'15"S, 49°13'27"W	Cerrado	BG-11
CCIBt3280	*Phormidium* sp.	18°59'00"S, 56°39'35"W	Pantanal	BG-11
CCIBt3290	*Nostoc* sp.	23º20'S 45º09'W	Atlantic Forest	BG-0
CCIBt3291	*Nostoc* sp.	23º20'S 45º09'W	Atlantic Forest	BG-0
CCIBt3304	*Calothrix* sp.	23°54'S,46°11'W	Atlantic Forest	BG-0
CCIBt3324	*Leptolyngbya* sp.	25º04'12"S, 47º55'27"W	Atlantic Forest	BG-11
CCIBt3332	*Leptolyngbya* sp.	21º59'46"S 47º25'33"W	Cerrado	BG-11
CCIBt3333	*Leptolyngbya* sp.	21º59'46"S 47º25'33"W	Cerrado	BG-11
CCIBt3448	*Phormidium* sp.	19°34'S, 57°01'W	Pantanal	BG-11
CCIBt3468	*Phormidium* sp.	19°34'S, 57°01'W	Pantanal	BG-11
CCIBt3579	*Calothrix* sp.	23º20'S, 45º08'W	Atlantic Forest	BG-0
CCIBt3581	*Calothrix* sp.	23º54'S, 46º11'W	Atlantic Forest	BG-0
CCIBt3582	*Calothrix* sp.	25º04'12"S, 47º55'27"W	Atlantic Forest	BG-0
CCIBt3585	*Calothrix* sp.	25º04'12"S, 47º55'27"W	Atlantic Forest	BG-0

Table S2. Extract and fraction mass obtained from each cyanobacterial strain

Code	Strain	Ext (mg)	F 1 (mg)	F 2 (mg)	F 3 (mg)	F 4 (mg)	F 5 (mg)	F 6 (mg)	Yield (%)
CCIBt3095	*Phormidium* sp.	101.4	31.4	7.10	8.2	11.8	10.2	6.6	91.8
CCIBt3213	*G. unigranulatum*	310.0	118.2	11.5	7.3	25.4	17.3	25.2	72.0
CCIBt3241	*Geitlerinema* sp.	188.1	12.7	5.0	12.2	26.6	25.0	12.5	64.5
CCIBt3247	*Nostoc* sp.	246.4	112.2	12.1	5.5	9.9	16.6	6.9	74.8
CCIBt3248	*Nostoc* sp.	100.0	4.9	4.3	3.3	4.0	3.1	2.2	42.9
CCIBt3278	*Phormidium* sp.	96.4	16.9	2.8	4.9	7.7	8.6	5.7	64.2
CCIBt3280	*Phormidium* sp.	122.5	18.6	6.1	10.3	17.4	31.7	20.3	118.0
CCIBt3290	*Nostoc* sp.	92.0	16.0	4.5	4.1	12.5	12.0	41.1	103.4
CCIBt3291	*Nostoc* sp.	61.0	20.0	2.0	4.0	6.0	4.0	1.0	63.9
CCIBt3304	*Calothrix* sp.	98.7	23.2	5.6	6.8	10.3	12.7	7.5	93.0
CCIBt3324	*Leptolyngbya* sp.	166.4	61.3	19.1	13.7	12.6	9.4	4.6	81.0
CCIBt3332	*Leptolyngbya* sp.	271.0	100.0	16.0	17.0	30.0	25.0	11.0	80.8
CCIBt3333	*Leptolyngbya* sp.	95.0	37.0	7.0	6.0	6.0	5.0	3.0	78.9
CCIBt3448	*Phormidium* sp.	271.0	57.0	22.0	16.0	43.0	45.0	14.0	74.0
CCIBt3468	*Phormidium* sp.	412.0	160.0	12.0	14.0	18.0	35.0	16.0	74.2
CCIBt3579	*Calothrix* sp.	93.0	51.2	11.2	10.7	13.0	9.6	9.1	132.4
CCIBt3581	*Calothrix* sp.	99.8	19.5	4.0	10.0	11.6	11.0	5.7	77.8
CCIBt3582	*Calothrix* sp.	92.1	22.4	5.1	5.5	6.6	9.4	5.2	77.2
CCIBt3585	*Calothrix* sp.	58.4	13.1	2.7	3.2	5.7	7.6	3.0	82.5

Table S3. Percentage of microbial inhibition for cyanobacterial extracts and fractions at 50 µg/mL. E.c. = *E. coli*, K.p.= *K. pneumoniae*, P.a. = *Pseudomonas aeruginosa*, S.m= *S. marcescens*, S.a.= *S. aureus*, SD= Standard deviation. SEM = Standard Error of the Mean

	Bioassay Results																			
**Sample**	***E. c.i* (50)**		***K. p.* (50)**		***P. a.* (50)**		***S. m.* (50)**		***S. a.* (50)**		***L. a. (25)***		**HCT-116(50)**		**MCF-7 (50)**		**HCT-116 (5)**		**MCF-7 (5)**	
	Mean	*SD*	*Mean*	*SD*	*Mean*	*SD*	*Mean*	*SD*	*Mean*	*SD*	*Mean*	*SD*	Mean	SEM	Mean	SEM	Mean	SEM	Mean	SE
CCIBt3095 EXT	-17,1	2,9	-1,4	15,2	-22,1	15,6	1,4	5,9	8,9	1,4	-15,5	15,94	96,09	0,74	12,91	8,55	18,82	7,66	-	-
CCIBt3095 F1	-12,2	2	-10,1	25,5	-27,3	36,5	1,9	4,6	16,5	2,3	85,8	3,41	78,8	5,6	-11,1	17,1	17,9	8	-	-
CCIBt3095 F2	-10,7	9,3	-18,5	14,9	32,1	24	2,9	5,3	40	31,9	28,46	7,06	93,3	2	41,2	18	36,2	7,4	-	-
CCIBt3095 F3	-7,3	1,8	-14,1	6,8	96,7	15	3,7	8	4,5	1,9	87,17	1,7	91,8	2	9,8	30,5	33,5	7	-	-
CCIBt3095 F4	-5,3	2,3	-6,2	21,2	38,6	62,1	2,1	6,3	-0,1	3,7	62,86	5,75	97,5	1,2	76,6	9,7	37,2	6,6	-	-
CCIBt3095 F5	-10,1	10,2	-5,9	7,5	92,4	11,1	1	7,1	97,9	0,2	86,62	3,78	100,2	0,3	77,5	14,7	39,7	13,6	-	-
CCIBt3095 F6	-4,6	10,5	-21	1	81,5	3,3	-1,4	6	-130	9,7	5,79	10,65	98,2	1,8	101,3	1,5	26,5	8,2	-	-
CCIBt3213 EXT	-14	9	-2,8	3	15	4,2	2,1	1,4	12,4	12,2	-17,49	10,96	19,98	6,25	26,61	4,55	-	-	-	-
CCIBt3213 F1	-13,1	13,5	3,9	8,5	32,8	2,9	4,3	1,9	21,1	7,2	4,44	6,92	-19,4	4,4	-3,7	7,2	-	-	-	-
CCIBt3213 F2	-14,7	5,7	9,8	1,9	28,5	3,7	1,1	2,1	13,5	7,4	-62,82	15,53	9,7	3,9	9,4	1,1	-	-	-	-
CCIBt3213 F3	-26,1	6,6	13,1	4,6	26,5	7	-7	1,9	17,4	9	52,11	13,24	14,2	6,7	19,8	0,7	-	-	-	-
CCIBt3213 F4	-30,7	12,4	-6,9	22,5	2,3	16,5	-10,9	6,3	21	5,4	-25,49	7,91	24,4	4,5	25,1	3,7	-	-	-	-
CCIBt3213 F5	-12,3	9,3	2,2	5,6	-3,8	14	-8,5	3,3	16,9	1,8	62,78	12,84	28	6,5	4	2,2	-	-	-	-
CCIBt3213 F6	-28,8	6	15,1	3	4,3	14,3	-3,8	4,9	3,6	8,1	-14,29	8,81	0,4	2,1	8,6	3,5	-	-	-	-
CCIBt3241 EXT	-9,5	10,2	-11,2	5,1	17,6	10,5	3,6	2,3	-9	4,5	-109,7	26,8	45,18	2,03	39,86	6,71	-	-	-	-
CCIBt3241 F1	-4,1	7,9	31,3	3,2	-1,1	11,7	6,8	3,9	34,3	4,3	-162,31	19,98	34	2,3	-0,5	11,2	-	-	-	-
CCIBt3241 F2	-15	3,9	11,8	4,8	83,7	7,7	7,2	3,9	28,3	1,7	-29,76	20,25	52,4	2,8	34,7	9,1	-	-	-	-
CCIBt3241 F3	-10,5	10,6	6,6	4,2	92,8	5,9	9,1	5,1	35,3	0,2	-82,88	19,99	51,1	16,1	5,9	7,2	-	-	-	-
CCIBt3241 F4	-17,3	4,2	21,9	5,9	98,4	2,1	7,5	6,3	-24,7	9,3	17,26	11,84	52,4	16,1	12,7	4,4	-	-	-	-
CCIBt3241 F5	-20,7	4,5	22,9	6,3	85,3	7,2	5,7	6,4	-46,7	8	-41,23	23,7	71	9,2	23	6,2	-	-	-	-
CCIBt3241 F6	-29,6	2,8	1,5	3,1	87,9	4,4	0,8	5,4	-94,1	9,9	41,02	22,68	52,5	8,3	71,5	3,8	-	-	-	-
CCIBt3247 EXT	-37	10,7	20,9	13,5	-44,5	6,6	-1,6	3,3	15,3	0,4	57,68	4,51	14,86	1,72	-0,98	15	-	-	-	-
CCIBt3247 F1	-30,5	10,5	21,3	2,2	-28,1	4,4	-0,9	3,4	24,8	2,1	3,06	16,79	7,9	6,6	-53,7	5,9	-	-	-	-
CCIBt3247 F2	-31,2	10,5	20	4,3	-38,1	1,4	-1,5	3,1	97,3	0,4	61,22	18,97	22,5	7,6	3,8	6,1	-	-	-	-
CCIBt3247 F3	-29,9	12,6	12,8	8	-32,1	18,9	0,1	2,4	38,2	7,4	32,55	14,66	41,6	5,9	-21,2	7,6	-	-	-	-
CCIBt3247 F4	-30,5	13	-1,8	6,1	54,4	23	-0,6	5,5	-1	7,4	80,34	4,33	52,9	6,1	-9,5	5	-	-	-	-
CCIBt3247 F5	-26,1	13	2,7	5,8	62,8	20,7	-2,4	2	26,2	0,4	-12,77	7,34	89,1	3,8	6,4	4,9	32,8	10,9	-	-
CCIBt3247 F6	-25,9	7,4	-12,5	5,7	84,3	2,2	-6,6	3,7	-52,7	0	59,86	4,92	53,5	14,2	-0,8	12,3	25,2	9,1	-	-
CCIBt3248 EXT	-17,4	8	-8,3	7,9	84,2	10,5	0,6	2,6	-93,6	3,1	52,98	19,13	79,56	1,12	40,33	5,89	-	-	-	-
CCIBt3248 F1	-12,9	12,6	-10,3	20,5	26,6	26,3	4,2	4,9	-71,9	3,2	-29,76	110,68	91,6	3,3	64,4	4,4	-	-	-	-

CCIBt3248 F2	-15	6,9	-15,9	3,6	94,8	2,3	6,4	7,3	-117	22	68,05	21,32	46,1	10,3	39,7	5,7	-	-	-	-
CCIBt3248 F3	-15,4	6,7	3,5	6,8	62,3	12,3	6,2	4,5	-54,9	0,6	-65,31	60,70	91,4	3,3	28,8	3,4	-	-	-	-
CCIBt3248 F4	-11,3	6	-2,2	13,9	86,2	12,2	8,07	9,4	-50,3	4,9	61,99	33,13	98,1	1,3	20,9	1,9	-4,9	6,9	-	-
CCIBt3248 F5	-18,8	9,4	-7,4	13	92,5	2,5	3,5	4,8	-75,2	3,4	-30,58	38,50	94,9	1,3	21,8	3,4	-1	13	-	-
CCIBt3248 F6	-17,9	5,9	-25,3	6,5	82,2	2,1	-1,2	4,3	-70,6	18,2	40,2	76,40	11,3	6,3	9,6	9,4	-8,5	13,5	-	-
CCIBt3255 EXT	-17,1	9,1	-12	7,5	-68,9	10,4	-7,5	2	36,4	23	-63,56	12,2	13,02	9,99	28,41	5,08	-	-	-	-
CCIBt3255 F1	-15,8	9,3	5	5,1	-77,3	15,8	-3,3	3,7	17,6	5	0,91	22,26	-4,5	5,3	7,2	1	-	-	-	-
CCIBt3255 F2	-8	1	-15,8	10,2	-72	17,3	-14,6	3,4	27,6	6,1	38,11	12,05	8,6	12,2	18,7	12,5	-	-	-	-
CCIBt3255 F3	-17,2	4,7	-5,7	1,1	-46,8	22,1	-15,4	4,7	24,4	5,1	45,71	14,22	-5,5	15,1	23,6	2,8	-	-	-	-
CCIBt3255 F4	-8,2	8,7	-13,4	22,3	-65,3	9,4	-18,7	3,6	29,6	5,1	26,51	19,21	18,6	7	31,3	6	-	-	-	-
CCIBt3255 F5	-18	9	-11,8	2,4	-61,1	2,8	-15,2	3,7	28,9	8,6	39,04	13,07	43,4	15,4	5,2	13,5	-	-	-	-
CCIBt3255 F6	-33,7	9,6	-12,6	7,9	-60,1	23,1	-13,6	5,4	15	11,1	17,44	7,09	51	23,2	32,4	3,2	27,3	9,4	-	-
CCIBt3278 EXT	-5,2	8,6	4,7	15,9	-0,6	13,8	-0,8	4,1	-73,3	6	16,77	4,6	96,69	0,49	77,88	3,34	15,71	7,54	-0,35	7,23
CCIBt3278 F1	-2,2	10,7	16,7	5,3	-19,1	20,8	6,8	2	-73,1	5,3	-24,03	2,46	89,4	2,5	44,1	2	22,9	12,5	-41,7	7,4
CCIBt3278 F2	-10,5	14	-13,8	6,2	91,9	3,3	5,2	5,5	-85,2	6,9	75,42	6,59	94,9	1,5	63,1	13,1	35,3	3	-35,3	15,02
CCIBt3278 F3	1,8	9,6	-12	9,3	90,7	1,9	4,8	4,8	-13,7	30,8	101,64	0,95	98,6	1,1	74,6	10	52,3	9,7	-3,1	1,36
CCIBt3278 F4	0,3	10,2	-7,8	5,3	60,6	21,6	5,5	5,7	10,7	1,3	95,41	4,73	97,3	1,7	85,6	3,3	53,9	11,1	17,6	9,63
CCIBt3278 F5	2,5	15,3	-34,9	4,9	-19,2	9,7	6	4,2	-102,2	6,7	40,69	26,8	98,8	0,9	80,7	5,8	44,2	16,9	-3,5	5,07
CCIBt3278 F6	-3,5	10	-30,7	3,2	75,4	13,4	4	4,1	-50,4	36,9	66,09	7,06	98,9	0,8	82,1	8,1	37	10	2,7	3,48
CCIBt3280 EXT	-9,7	6,1	-24,1	6,2	-3,9	23,5	1,74	6,6	-58,8	23	-97,83	8,69	92,45	4,63	70,64	5,95	26,92	9,92	-48,2	20,3
CCIBt3280 F1	-1,1	2,5	3,7	18,4	9	11	6,27	6,6	57,1	15,2	1,42	15,6	11	11,4	-8,3	2,6	-	-	-	-
CCIBt3280 F2	-5,9	6,1	-16,9	6,5	1,4	5,7	5,84	8,3	57	11,9	-106,4	18,8	82,7	1,8	26,9	2,8	28,4	3,8	-	-
CCIBt3280 F3	2,6	3,5	-11	10,9	69,8	3	5,04	10	-30,8	16	104,9	1,77	99,7	1	90,4	2,2	88,4	0,7	24,7	5,82
CCIBt3280 F4	-10,9	20,3	-23,3	4,1	90,7	2,2	1,77	9,1	51,8	15,7	101,2	4,46	100,1	0,7	93,6	1,2	98,7	0,5	64	6,6
CCIBt3280 F5	-3	6	-25,5	5,4	78,9	13,3	1,54	8,9	-7,8	8,6	54,94	3,75	99,8	0,4	86,5	4,8	66,1	15,6	37,7	14,47
CCIBt3280 F6	-0,1	10,4	-22,3	5,8	86,7	2	-0,43	4,8	-58,8	22,2	-2,12	6,82	97,9	1,5	87	3,4	64	12,4	28	8,61
CCIBt3290 EXT	-31	3,9	1,9	6,2	18	8,1	-13,4	4,1	102	3,5	26,24	17,6	70,19	9,97	50,51	7,08	37,04	9,18	-	-
CCIBt3290 F1	-36	12,2	14,1	1,4	17,6	2,7	3,9	2,9	-17,3	7	39,58	5,78	-2,5	2,8	-14,1	5,5	18,5	18,4	-	-
CCIBt3290 F2	-43,3	11,4	0,7	1,4	59,8	12,3	-10,8	8,9	90,7	18,6	69,18	3,45	58	15,9	63,9	16,5	13,6	23,9	-	-
CCIBt3290 F3	-33,2	116	16	4,1	79,5	5,5	-10,1	12,6	102,3	3,1	49,18	6,21	33,8	17	36,3	22,5	17,4	24,4	-	-
CCIBt3290 F4	-37,1	12,5	11,5	2,2	71,3	9,6	-20	8,8	102,3	1,8	58,44	8,53	94,6	4,7	73,7	8,3	29,4	15,6	-	-
CCIBt3290 F5	-62,3	2,1	8,6	3,6	25,4	10,3	-21,1	9,9	85,1	15,7	42,11	10,4	88,9	9,3	39	8,7	23,3	17,9	-	-
CCIBt3290 F6	-41,2	7,3	14	9,8	14,4	5,8	-18,3	7,9	-10,1	13,5	43,58	10,2	-7,9	6,6	-13,4	5,2	20,4	16,3	-	-
CCIBt3291 EXT	13,3	4,4	1,8	16,6	101,4	17,8	1,2	3	9,4	10,2	91,5	6,82	97,95	1,99	13,86	3,48	26,81	11,9	-	-
CCIBt3291 F1	14,2	2	-8,2	4,4	97,5	11,5	5,4	4,8	-1,1	14,2	61,77	19,4	9,2	4,8	10,3	4,6	-11,1	5,3	-	-
CCIBt3291 F2	1,4	11,2	-10,7	1,7	109,7	6,4	-5,1	3	21,4	4,9	46,66	7,03	48	15,2	11,6	1,1	-7,7	10,7	-	-

CCIBt3291 F3	3,6	8	-4,9	5,8	91,4	6,5	-9,2	4,6	47,2	1,2	126,2	11,2	89,8	8,1	6,3	4	5,7	14,3	-	-
CCIBt3291 F4	4,7	1,8	-1,6	8,8	82,1	5	-11	6,2	40,1	19,1	18,71	37,4	103,2	1,4	45,5	19,2	23,5	15,7	-	-
CCIBt3291 F5	-10,3	12,2	5,6	2,1	103,8	12	-12,5	8,7	6,3	4,9	70,38	38,3	100,5	1	-7,6	14,6	15,3	10,6	-	-
CCIBt3291 F6	3,7	3,9	4,4	0,7	80,9	9,2	-8,9	7,2	-1	12,2	103,5	13,8	104,5	1,8	25,6	8,3	18,9	14,2	-	-
CCIBt3304 EXT	17,5	3,8	1,7	7,7	27,5	5,5	-23,6	2,6	4,1	6,3	5,47	10,1	85,19	9,18	14,06	0	36,15	15,3	-	-
CCIBt3304 F1	18,6	3,8	3,9	8,4	9,8	4,4	-12,3	3,4	6,8	9,6	-38,17	12,2	-11,3	7,7	13,7	1,8	23,5	19,9	-	-
CCIBt3304 F2	18,7	4,5	22,2	3,4	7,6	4,8	-14,1	3,8	2,2	3,4	-42,52	30	81,8	6,2	22,3	1,4	26,5	18,3	-	-
CCIBt3304 F3	21,4	6,4	13	13,7	99,4	0,6	-17,7	7,9	57,6	15,7	-49,08	12	88,8	4,2	10,2	5,7	29,2	18,2	-	-
CCIBt3304 F4	9,6	10,6	5,1	11,2	21,4	20,7	-23,9	5,4	31	13,4	35,49	25,2	99,1	2,5	48,1	23,9	42	16,2	-	-
CCIBt3304 F5	-12,5	5,7	0,9	8,1	-4,5	10,5	-32,1	0,4	3	16,7	-3,55	10,4	101,5	2,2	-0,3	23,4	37,6	17,6	-	-
CCIBt3304 F6	-1,8	11	25,9	17,1	-22	7,1	-27,2	3,7	5,2	25,2	16,27	14,7	95,2	1,3	19,3	2,8	33,1	18	-	-
CCIBt3324 EXT	-7,2	8,6	-23,6	4,8	40,1	12,1	97,7	3	-1,6	3,7	14,81	21,8	83,58	3,42	13,99	11,5	24,44	16,4	-	-
CCIBt3324 F1	-2,5	3,3	7,3	7,2	-46,5	7,7	94,6	1,9	10,4	0,6	5,14	13,3	3,8	10,5	-32,9	13,4	-	-	-	-
CCIBt3324 F2	8,6	8,7	8,7	9	-37,3	12,5	94,7	2,1	99,6	0,4	37,47	9,06	11,2	6,3	-9,6	16,8	-	-	-	-
CCIBt3324 F3	11,2	14,7	-13,8	9,4	3	8,4	93,3	3,9	99,6	0	42,93	0,95	43,9	10,8	-28,8	24,1	-	-	-	-
CCIBt3324 F4	-7,9	10,2	-16,5	8,8	92,6	12,4	93,4	0,98	52,8	12,2	97,27	2,5	95,7	0,8	14,1	26,8	37,3	9,7	-	-
CCIBt3324 F5	-16,2	3	-35,3	4,1	58,3	4,8	96,4	2,2	98,6	0,2	75,42	15,8	96,4	2,5	63,2	8	31,7	15,7	-	-
CCIBt3324 F6	-13,8	6,2	-35,5	2,4	67,8	3,3	95,8	8,6	-88,7	15	58,43	11,2	97,7	0,4	53,8	11,7	31,2	13,6	-	-
CCIBt3332 EXT	19,5	1,2	-8,7	5,6	18,2	7	-0,2	5,7	-4,4	11,4	91,5	22	-9,51	8,63	20,33	9,01	-	-	-	-
CCIBt3332 F1	18,7	4,3	-4,4	4,5	30,8	7,1	7,8	2,2	18,4	9,2	80,29	12,7	-5,8	7	0,2	9,5	-	-	-	-
CCIBt3332 F2	12,6	6,9	-1,8	6,3	15,4	14,9	2,3	8,5	17,4	11,3	83,9	32,6	-17,3	9,1	27,3	9,7	-	-	-	-
CCIBt3332 F3	12,7	7,9	4	3	21,1	10	-0,8	2,3	18,4	5,8	80,05	13,8	-17,9	10,2	13,8	0,3	-	-	-	-
CCIBt3332 F4	4,1	19,2	8,1	6,3	14,6	10,4	-1,8	5,3	14,5	12	66,16	14,1	-11	12,6	46,9	0,3	-	-	-	-
CCIBt3332 F5	8	15,3	5,7	6,9	18,5	9,7	-5,5	5,2	15,2	7,2	62,26	30,5	-2,5	6,1	11,6	9,6	-	-	-	-
CCIBt3332 F6	17,3	1,9	5,5	3,4	15,2	10,7	-8,8	7,3	11,7	8,9	74,93	24,1	4,2	11	15,4	1,7	-	-	-	-
CCIBt3333 EXT	-52,4	16	-6,3	7,9	-2,5	9,3	2,4	14	11,8	2,8	28,13	13,4	38,27	1,9	12,39	5,98	-	-	-	-
CCIBt3333 F1	-43,4	9,6	14,7	4,4	24,2	4,7	19,3	25,7	40,1	29,4	71,27	11,4	-6,5	6,7	18,9	9,3	-	-	-	-
CCIBt3333 F2	-42,7	12,6	11,3	1,7	0,6	14	10,6	22,8	44,2	6,1	-4,04	22,8	-4,6	3,4	16	6,5	-	-	-	-
CCIBt3333 F3	-53,4	31,6	5,4	6,1	-1,3	20	6,3	29,9	12,4	2,9	45,36	14,9	12,8	4,6	3,9	16,7	-	-	-	-
CCIBt3333 F4	-41,7	21	6,9	4,2	15	3,9	-5,7	11,2	17	0,7	1,16	27,3	57,9	12,7	12,8	11,5	15,5	8,6	-	-
CCIBt3333 F5	-43,8	10,7	9,8	3,6	0,9	3,8	6	32	2,9	6,4	38,86	9,39	73,5	9,3	1,9	19,2	15,6	13,2	-	-
CCIBt3333 F6	-63,4	21,3	13	2,8	-7	16,7	-2,3	21,4	-5,5	9,9	36,91	15,5	76	6,3	8,5	12	19,3	13	-	-
CCIBt3448 EXT	-30,6	10,9	-1	9,4	-15,8	6,1	-7,4	1,6	-36,6	12,8	66,16	2,92	11,7	2,51	25,63	5,42	4,94	6,65	16	3,56
CCIBt3448 F1	-25,1	2,1	18,1	1,5	10,2	5,3	-9,6	3,8	-13,2	13,3	45,1	13,2	-8,3	7,9	13	3,8	-5,8	5,9	10,2	2,79
CCIBt3448 F2	-30	10,5	15	8,3	2	5	-8,6	5,9	6,2	11,9	51,86	9,27	14,3	2,7	18,3	7,1	15,6	2,9	17,3	4,1
CCIBt3448 F3	-26,9	14,6	5,9	8,9	0,8	6,2	-3,4	11,5	1,4	15,5	51,92	8,23	12,3	4,2	-0,9	22,5	18,1	3,1	9,1	12,0

CCIBt3448 F4	-35,3	8,2	9,3	5,4	-13,2	13,8	-4,7	7,1	-0,5	18	47,88	15,9	8,9	7,8	-24	23,6	0,2	2,2	7,7	3,1
CCIBt3448 F5	-26,9	6,2	9,5	1,2	-3,7	6,5	-3,5	4,7	-6,7	17,1	19,85	4,68	-0,4	11,1	-15	15,4	-10,7	12,8	-4,3	2,8
CCIBt3448 F6	-36,9	6,7	6,9	7,9	-3,6	10,2	-13,9	12,7	0,9	10	98,91	10,9	18,9	1,4	-6,4	26,6	9,9	5,1	-12,6	6,1
CCIBt3468 EXT	4,6	2,9	-7,9	11,1	-1,4	6,6	-7,8	5,9	-0,3	19	94,75	3,42	74,36	12,4	22,59	4,34	26,8	3,81	-	-
CCIBt3468 F1	12,9	1	-1,6	4,5	23,1	4,8	-6,8	6,1	6,3	5,5	97,35	1,95	-22,1	3,7	-2,7	4	-12,1	8,7	-	-
CCIBt3468 F2	-0,9	5,3	-0,8	6,4	-14	11,6	-13,2	6,7	-6,4	3,3	38,86	47,2	28,9	19,6	10,2	4,6	-4,2	3,1	-	-
CCIBt3468 F3	13,7	4,8	-0,1	4,2	64,9	7,8	-13,2	1,8	83,7	7	63,23	20,5	40,8	25,7	16,3	4,2	2,9	6,8	-	-
CCIBt3468 F4	2,8	7,9	1,8	10	45,9	6,4	-24,3	3	74,5	5,5	91,99	1,87	97,2	2,5	51	2,3	20,5	10,5	-	-
CCIBt3468 F5	-20,4	22,4	-0,6	3,9	2,1	5,5	-17,6	8,8	57	4,8	96,87	14,2	94,3	4	27,1	6,4	20,9	12,4	-	-
CCIBt3468 F6	-4,3	5,5	3,2	8,9	6,4	5,3	-19,2	5,8	16	3,1	85,17	5,6	95,2	1,9	21	10	24,8	8	-	-
CCIBt3579 EXT	-10,1	6,7	-1,3	3,3	0,2	2,3	-7,3	5,1	9,8	4	19,75	21,3	28,72	0,61	12,97	0,63	-	-	-	-
CCIBt3579 F1	-30,6	10,6	-4,1	6,5	-1,6	6	-5,3	4,1	3,5	10	36,09	8,44	-14,1	4,9	-21,6	8,8	-	-	-	-
CCIBt3579 F2	-29,3	8	-3,1	5,9	0,4	6	-11,9	3,6	3,8	8,2	78,68	3,54	-1,5	8,5	11,9	7,4	-	-	-	-
CCIBt3579 F3	-40,5	26,5	-4,9	8,8	11,9	9,2	-12,8	3,5	16,9	3,7	-3,68	21,6	1,8	12,5	0,1	5,3	-	-	-	-
CCIBt3579 F4	-27,8	6,8	2,1	6,1	-6,4	3,5	-10,4	8,7	16,5	7,1	31,14	9,47	16,2	10,2	15,5	1,1	-	-	-	-
CCIBt3579 F5	-48,9	13,3	-3,5	11,7	-32,7	4,8	-13,9	5	1,5	6	35,16	2,56	32,4	12,5	-41,6	3,8	-	-	-	-
CCIBt3579 F6	-40,3	8,8	3,5	2,9	-36	15,5	-16,9	6,1	-9,7	3,7	49,89	6,22	23,4	2,6	17,7	7,1	-	-	-	-
CCIBt3581 EXT	20,8	4,2	-6,2	6,8	-21,9	3,3	-23,4	4,9	0,8	16,6	1	12,6	90,53	6,12	22,77	4,01	36,79	16,3	-	-
CCIBt3581 F1	25,1	2,8	6,4	14	-5,6	3,7	-6,3	1,8	15,3	5,4	29,46	12	-11,9	5,7	11,8	5,6	23,7	18,1	-	-
CCIBt3581 F2	35,1	6	20,2	7,4	-26,7	6,3	0,2	6,1	15,6	8,1	52,57	14	25,5	6,6	26,4	8,4	26,1	18,5	-	-
CCIBt3581 F3	31,8	3,4	16	12,8	-17,8	2,6	-20,2	8	20,6	4,3	65,29	11,7	39,3	12,5	28	12,3	26,8	19,7	-	-
CCIBt3581 F4	24,7	4,1	21,9	19	24,4	6,9	-19,8	11,8	23,7	11,7	57,25	14	100,2	2,1	84,7	0,8	34	17,5	-	-
CCIBt3581 F5	1,6	17,1	-5,6	9,2	-19,1	5,5	-23	5,3	5,8	7,2	28,68	20,3	99,4	3	28,9	20,2	28,6	20,2	-	-
CCIBt3581 F6	-0,7	15,7	8,6	17,2	-21,4	7,1	-24,6	8,9	7,8	10,7	59,93	12,1	97,8	2,2	46,4	27,2	22,7	23,1	-	-
CCIBt3582 EXT	0,7	13,6	-37,1	2,5	-20,9	44,4	1,4	5,2	18,4	0	-425,8	1,07	97,5	0,26	84,28	3,06	37,92	14,5	8,7	6,6
CCIBt3582 F1	1,2	14,7	6,1	3,6	-34,7	18,5	4	3,7	39,8	0	-425,6	0,45	44,8	11,1	-21,7	3,7	32,9	9,1	-48,2	20,35
CCIBt3582 F2	5,5	13,7	-10	12,1	54,2	24,1	2,2	3	94,4	0,2	-54,33	9,22	78	8,6	76,3	4	48,3	10,2	24,7	5,82
CCIBt3582 F3	4,6	16,6	-9,3	12,8	80,1	9	2,7	5,2	98,4	0	71,49	7,17	97,5	0,9	68,5	11,5	64,8	14	64	6,6
CCIBt3582 F4	1,1	12,3	-26,3	5	83	1,7	5	4,1	-0,5	16,1	25,45	11,8	99,2	0,2	80,8	4,8	69,1	18,3	37,7	14,47
CCIBt3582 F5	-1,5	6,6	-34,5	9,1	-1,5	8,4	3,5	4,2	96,9	0,4	-58,37	22,2	101,3	0,2	87,2	3,8	63,8	18,9	28	8,61
CCIBt3582 F6	-6	11,6	-36,7	11,8	69,2	3,9	-0,1	4,8	-113,7	8,9	-14,36	9,56	99	0,3	90,6	3,5	96,6	6,6	-0,4	7,23
CCIBt3584 EXT	-41,3	17,4	-16,7	7,1	-15,5	6,7	-10,2	2,8	32,5	22,9	74,44	5,82	19,8	3,14	13,71	4,55	-	-	-	-
CCIBt3584 F1	-38	11,5	-8,6	7,4	-13,9	23,2	-5,9	3,8	2,3	10	58,11	15	-10,2	4,7	2,8	8,5	-	-	-	-
CCIBt3584 F2	-23,4	2,5	-9,2	6,2	-3,3	26,8	-15	7	7,6	13,6	85,78	8,43	-5,1	9	-5,3	15,1	-	-	-	-
CCIBt3584 F3	-24,5	3,1	-14,4	2,5	19,6	27,6	-18,2	5,5	12,4	14,5	60,78	11,1	-0,4	6,2	-0,8	4,7	-	-	-	-
CCIBt3584 F4	-25,2	8,8	-11,9	3,4	-8,1	38,1	-21,6	2,2	-2	10,9	77	10,4	16,8	4,2	11,1	8,5	-	-	-	-

CCIBt3584 F5	-35,6	13,7	-16,9	7,7	-5,1	25,8	-18,3	3	4	16,7	60,56	8,07	41,8	12,9	-23,6	11,2	-	-	-	-
CCIBt3584 F6	-52,1	4,7	-7	7,1	-19,2	29,2	-19,6	10,3	-4,4	14,8	71,22	7,38	6,7	4,1	1,1	4,7	-	-	-	-
CCIBt3585 EXT	-14,4	9,2	14,9	4,8	16,3	11,6	-2,2	4,4	-20,2	4,4	-9,71	23,4	92,9	5,23	37,65	0,88	29,93	15,2	-	-
CCIBt3585 F1	-9	2,1	25,7	5,2	6,9	8,7	4,2	4,7	-16,3	16,5	-33,55	16,2	-10,8	4,8	10,2	2,5	18,5	22,2	-	-
CCIBt3585 F2	-7,6	4	18,8	5,2	6,1	6,4	2,3	7,6	-7,7	10,7	-28,73	26,6	16	7,1	33,2	0,7	17,6	23	-	-
CCIBt3585 F3	-7,1	4,1	7,5	7	104,4	0,9	-8,8	5,6	69	10,6	-1,94	11	89,9	5,7	35	8,8	24,2	21,3	-	-
CCIBt3585 F4	-11,4	2	12,1	7,5	112,7	3,3	-9,1	5,3	76,9	11,2	18,75	10,2	98,8	3,2	85,3	0	28,2	17	-	-
CCIBt3585 F5	-11,9	13,2	10,3	8	74,1	14	-11,3	4,1	6,4	34,7	16,18	9,69	99,9	2,5	22,5	14,9	21,6	18,7	-	-
CCIBt3585 F6	-16,4	6,5	8,7	4,6	46,1	14,3	-9,5	7,7	1,7	25,1	41,85	11,7	94,8	1,7	53,5	1,3	16,9	17,6	-	-

Table S4. Bioactive features predicted by NP Analyst for each analyzed cyanobacterial strain

Code	Strain	NP Analyst Features
CCIBt3095	*Phormidium sp.*	124
CCIBt3213	*G. unigranulatum*	10
CCIBt3241	*Geitlerinema* sp.	8
CCIBt3247	*Nostoc* sp.	28
CCIBt3248	*Nostoc* sp.	19
CCIBt3278	*Phormidium* sp.	124
CCIBt3280	*Phormidium* sp.	240
CCIBt3290	*Nostoc* sp.	181
CCIBt3291	*Nostoc* sp.	163
CCIBt3304	*Calothrix* sp.	89
CCIBt3324	*Leptolyngbya* sp.	69
CCIBt3332	*Leptolyngbya* sp.	1
CCIBt3333	*Leptolyngbya* sp.	12
CCIBt3448	*Phormidium* sp.	0
CCIBt3468	*Phormidium* sp.	112
CCIBt3579	*Calothrix* sp.	2
CCIBt3581	*Calothrix* sp.	65
CCIBt3582	*Calothrix* sp.	125
CCIBt3585	*Calothrix* sp.	149

Figure S1. Prioritized features of cyanobacteria *Calothrix* sp. CCIBt3585 through NP Analyst and SIRIUS analysis. TIC: Total Ion Chromatogram, EIC: Extracted Ion Chromatogram. A) Community highlighted by NP Analyst. B) Scatter plot of features of interest. C) Heatmap of biological activities of the community. D) Chromatograms, molecular formulas, and error (ppm).

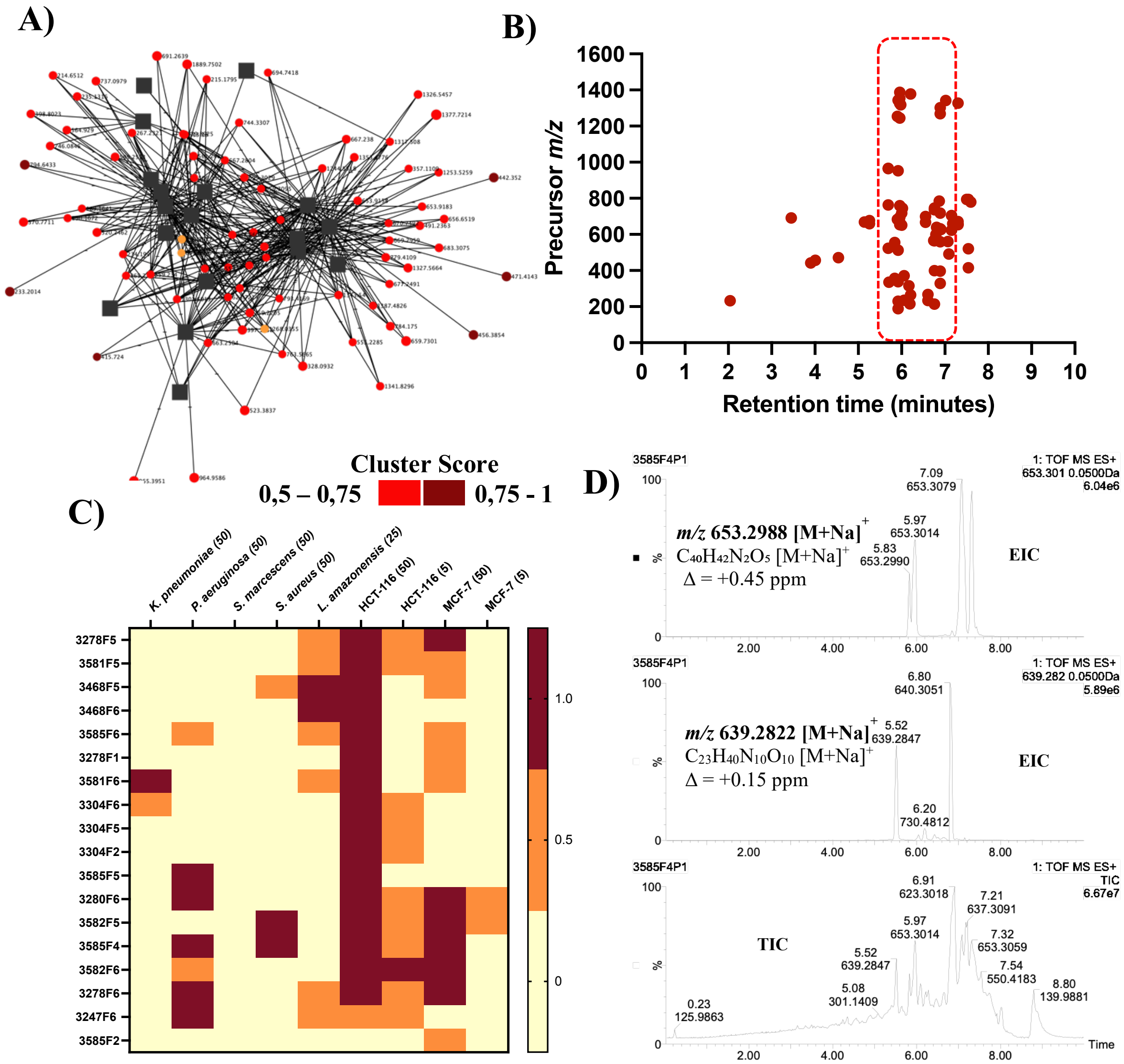

Figure S2. Prioritized features of cyanobacteria *Nostoc* sp. CCIBt3291 through NP Analyst and SIRIUS analysis. TIC: Total Ion Chromatogram, EIC: Extracted Ion Chromatogram. A) Community highlighted by NP Analyst. B) Scatter plot of features of interest. C) Heatmap of biological activities of the community. D) Chromatograms, molecular formulas, and error (ppm).

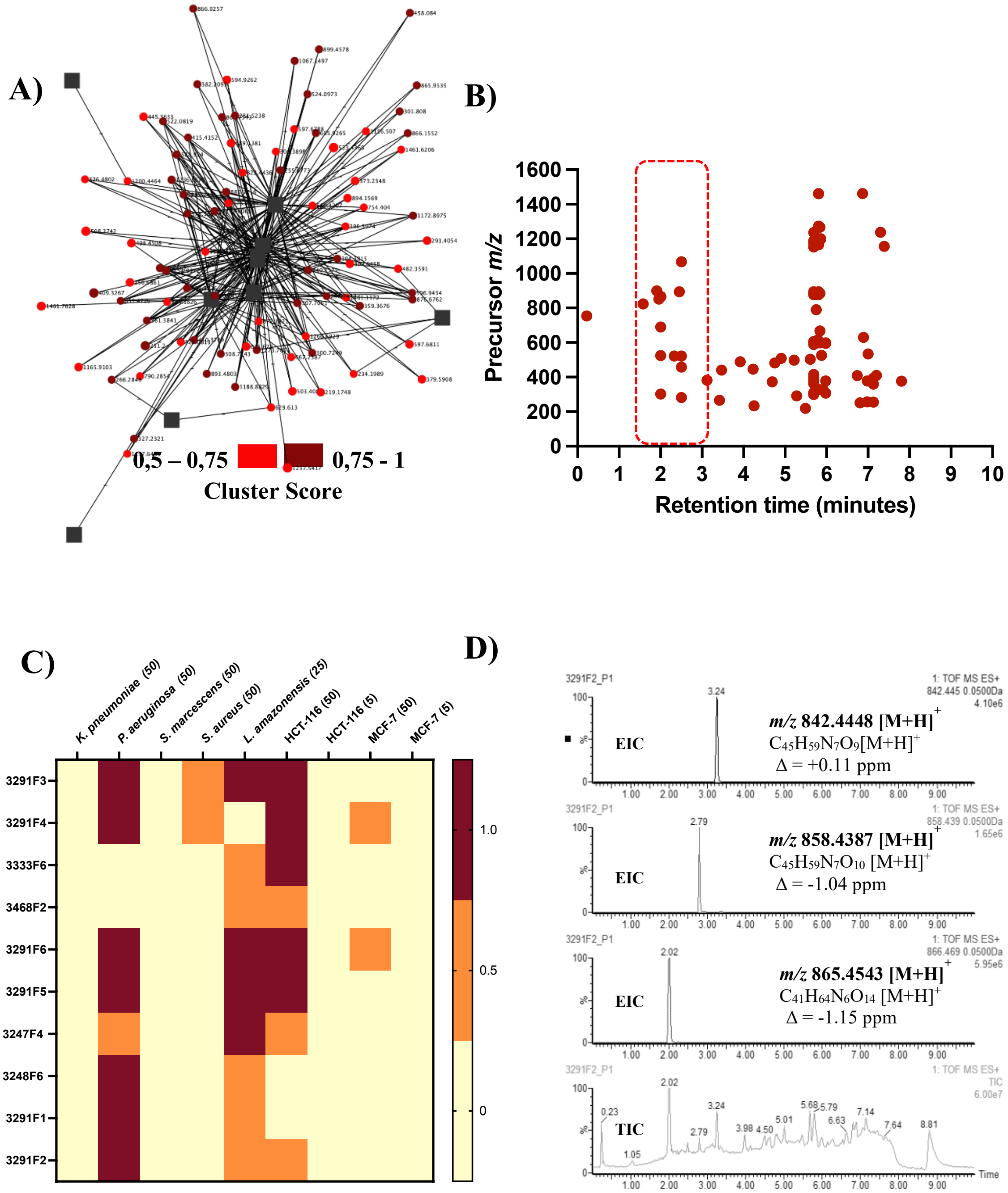

Figure S3. Prioritized features of cyanobacteria *Phormidium* sp. CCIBt3095 through NP Analyst and SIRIUS analysis. TIC: Total Ion Chromatogram, EIC: Extracted Ion Chromatogram. A) Community highlighted by NP Analyst. B) Scatter plot of features of interest. C) Heatmap of biological activities of the community. D) Chromatograms, molecular formulas, and error (ppm).

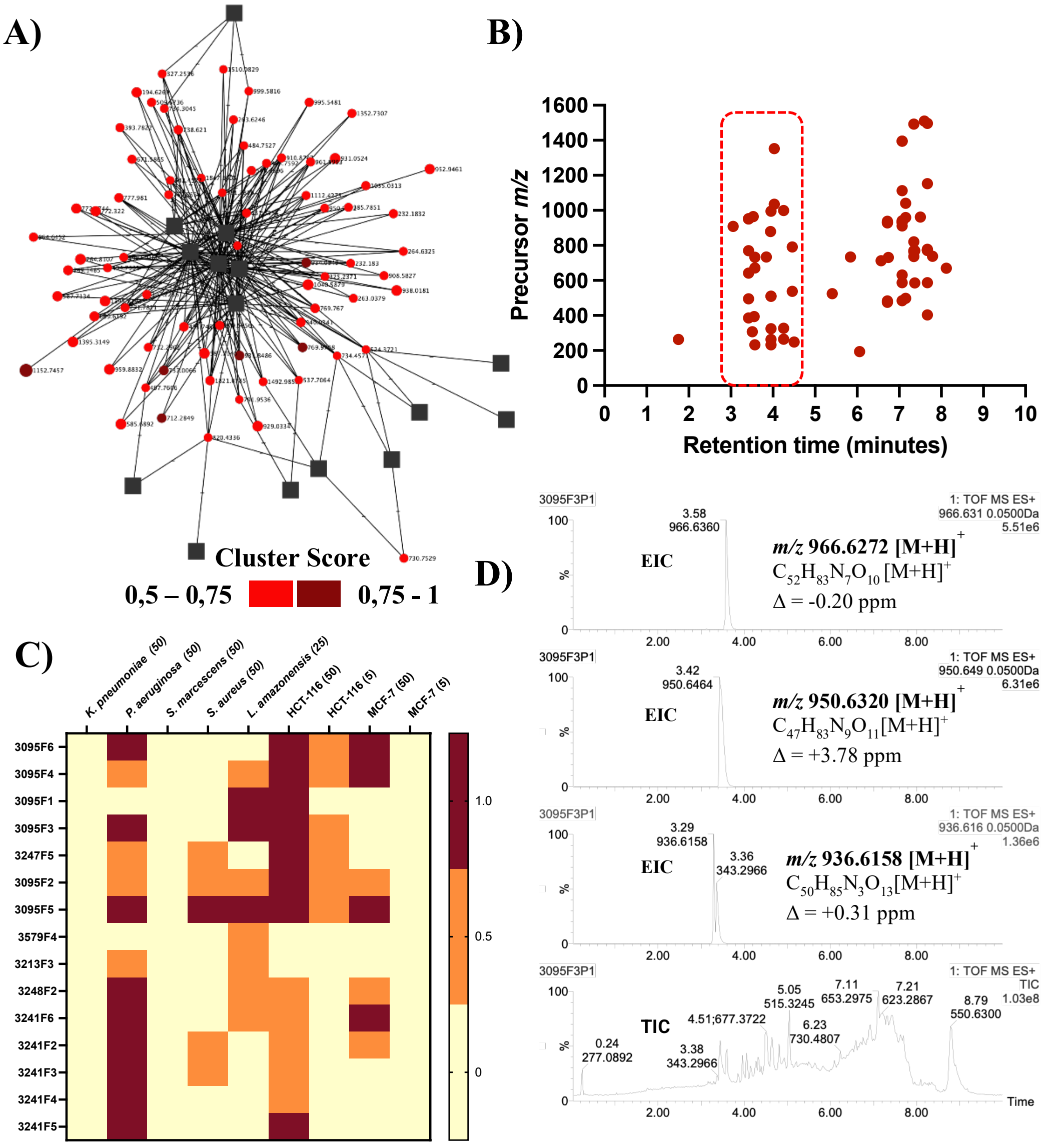

Figure S4. Prioritized features of cyanobacteria *Leptolyngbya* sp. CCIBt3324 through NP Analyst and SIRIUS analysis. TIC: Total Ion Chromatogram, EIC: Extracted Ion Chromatogram. A) Community highlighted by NP Analyst. B) Scatter plot of features of interest. C) Heatmap of biological activities of the community. D) Chromatograms, molecular formulas, and error (ppm).

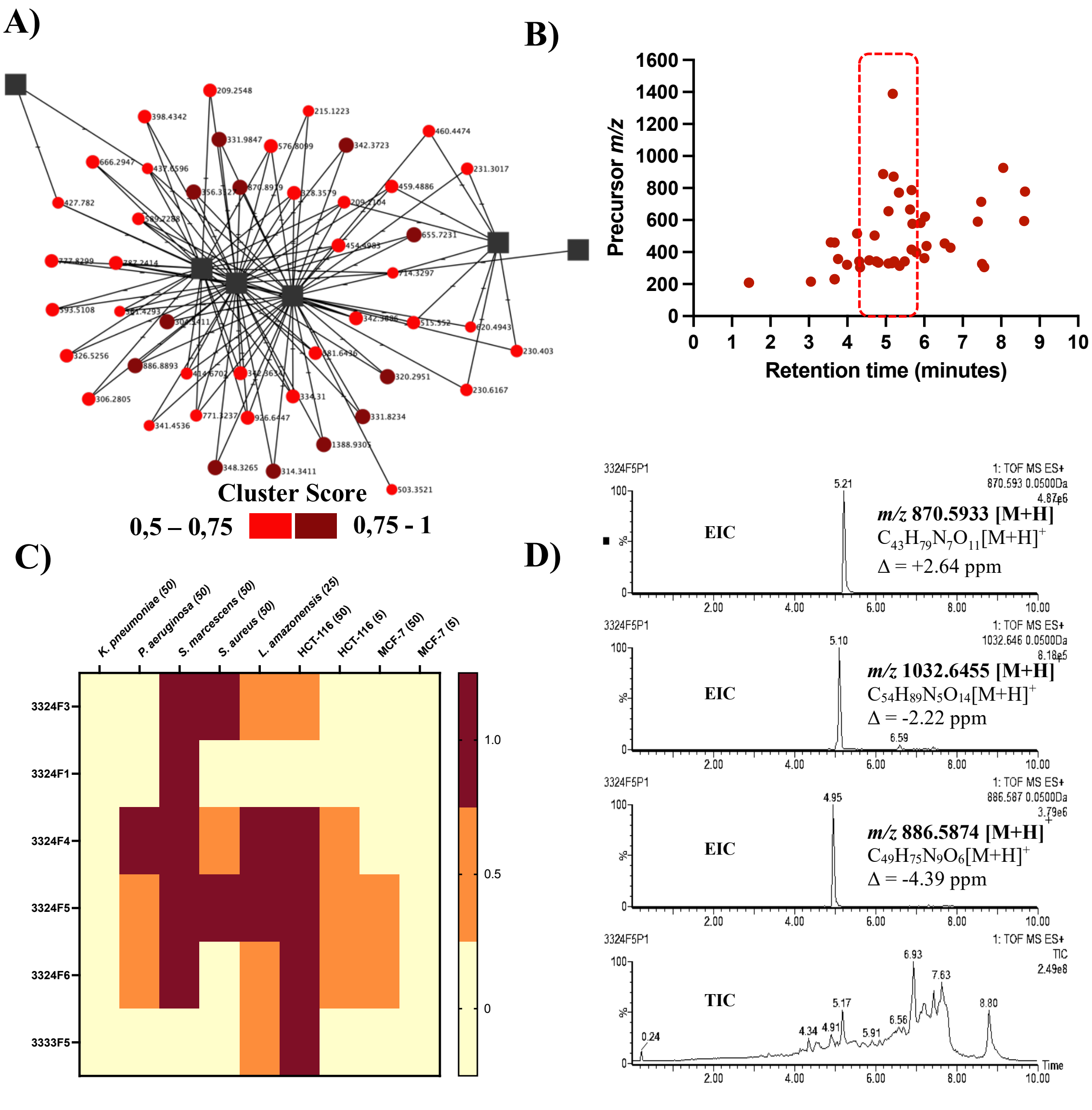

Figure S5. Prioritized features of cyanobacteria *Phormidium* sp. CCIBt3278 through NP Analyst and SIRIUS analysis. TIC: Total Ion Chromatogram, EIC: Extracted Ion Chromatogram. A) Community highlighted by NP Analyst. B) Scatter plot of features of interest. C) Heatmap of biological activities of the community. D) Chromatograms, molecular formulas, and error (ppm).

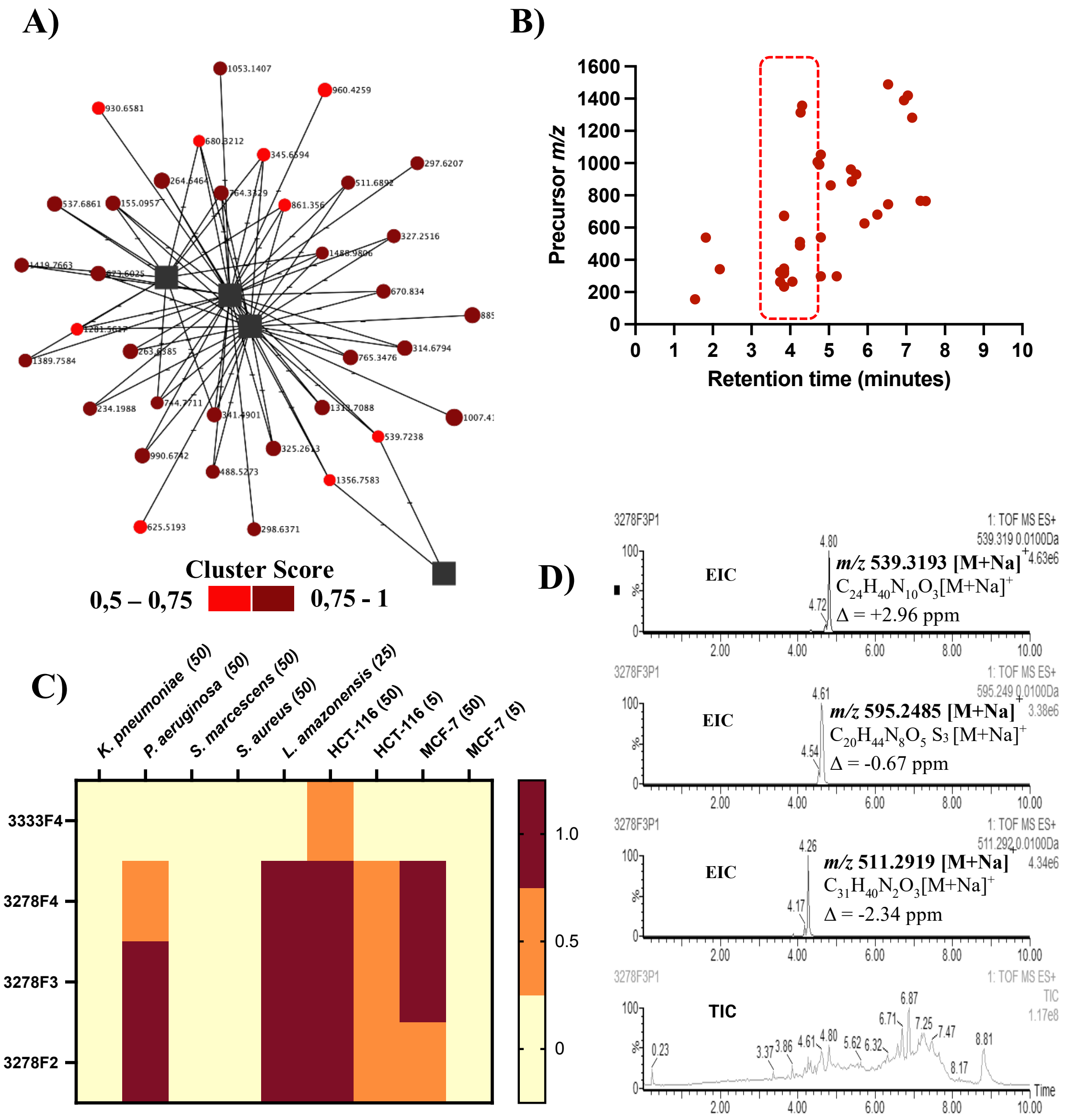

Figure S6. Prioritized features of cyanobacteria *Nostoc* sp. CCIBt3248 through NP Analyst and SIRIUS analysis. TIC: Total Ion Chromatogram, EIC: Extracted Ion Chromatogram. A) Community highlighted by NP Analyst. B) Scatter plot of features of interest. C) Heatmap of biological activities of the community. D) Chromatograms, molecular formulas, and error (ppm).

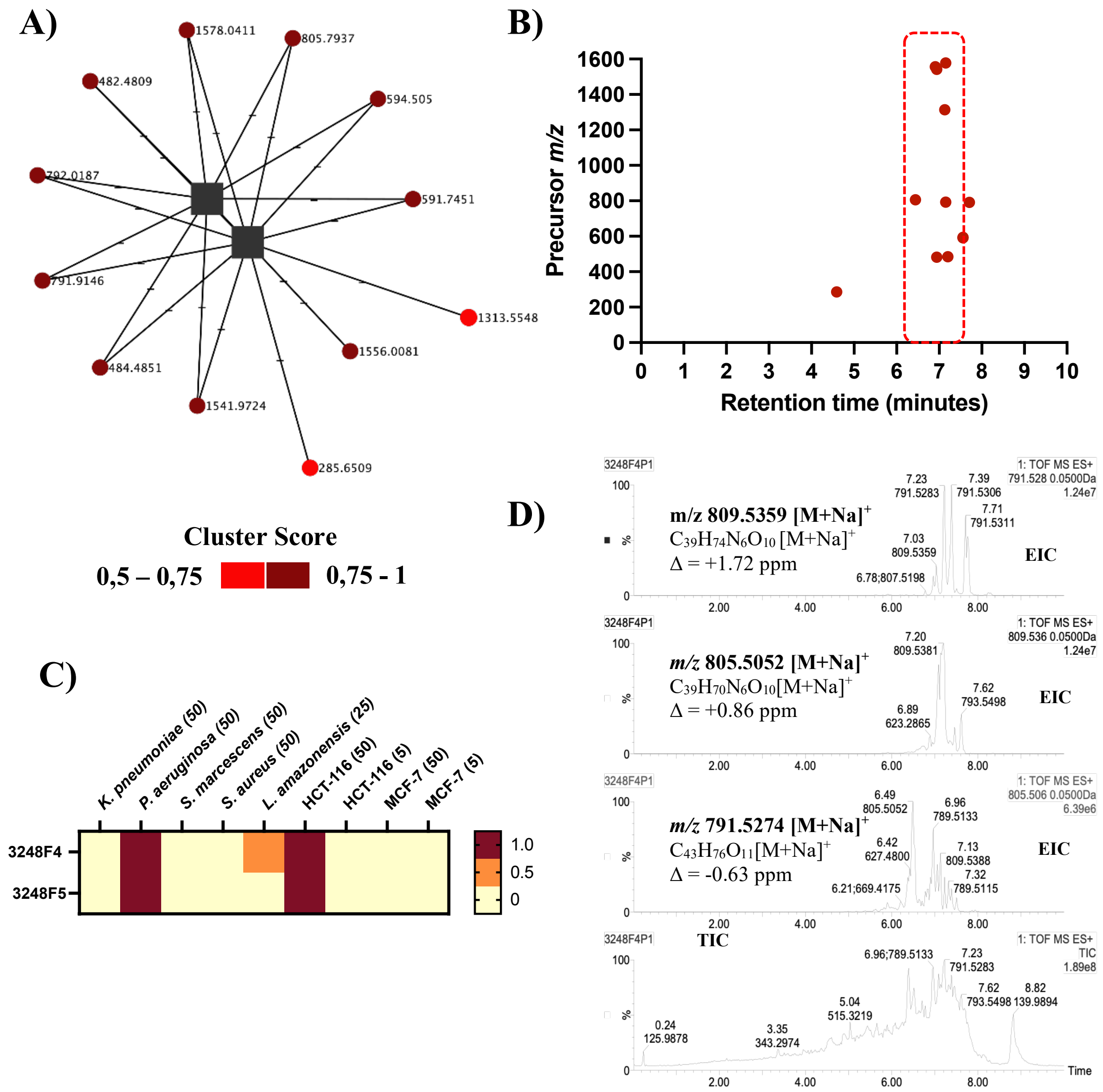

Figure S7. Molecular network of all cyanobacterial fractions analyzed using GNPS, where G1 (red): *Geitlerinema* sp., G2 (blue): *Nostoc* sp., G3 (green): *Phormidium* sp., G4 (orange): *Calothrix* sp., G5 (yellow): *Leptolyngbya* sp.

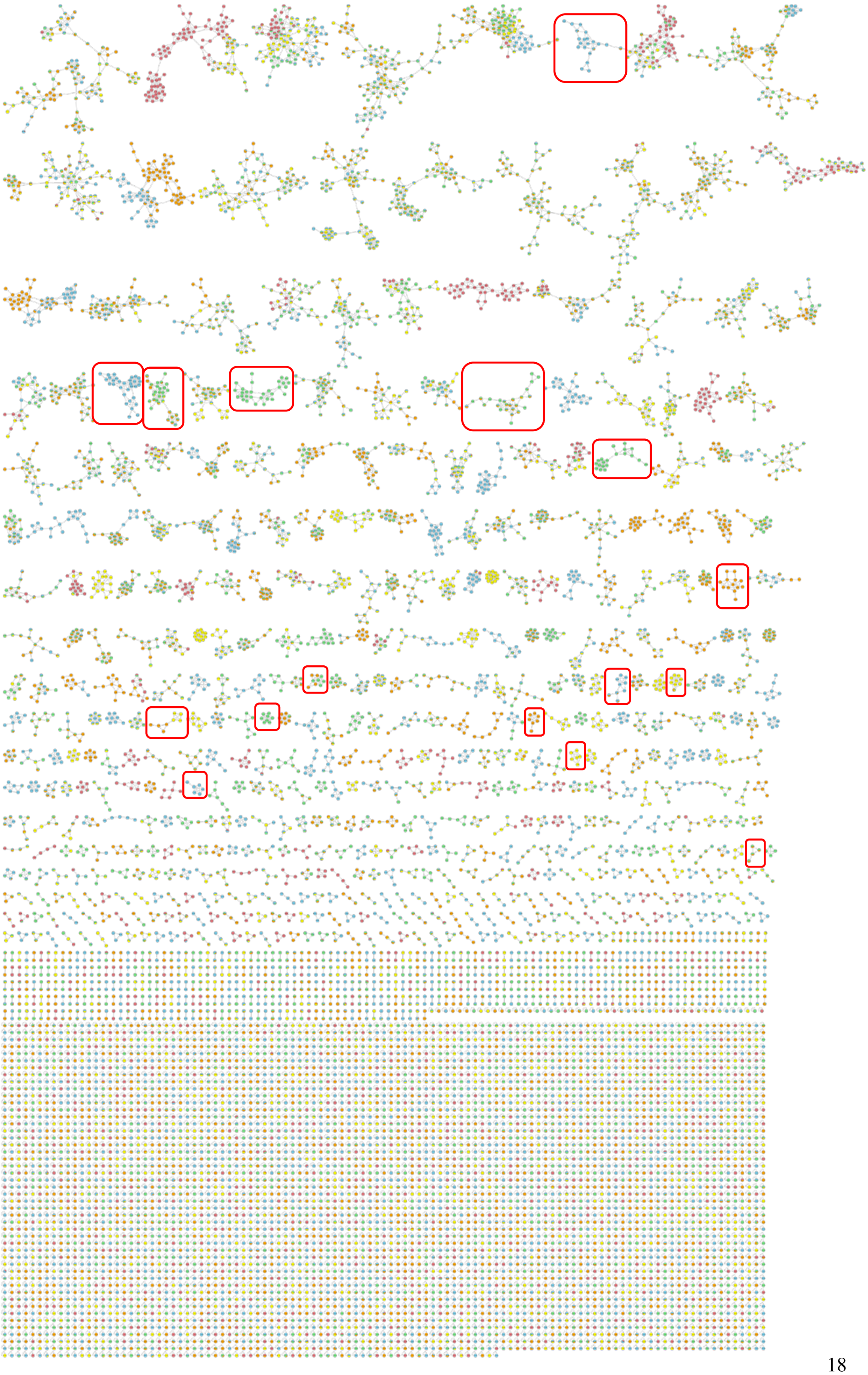

Figure S8. A) GNPS cluster of features from the cyanobacterium *Calothrix* sp. CCIBt3582 highlighting the compounds of interest indicated by NP Analyst as associated with the bioactivity. B) Total ion chromatogram (UHPLC-MS). C) MS1 spectra of the compounds of interest for community 1 indicated by NP analyst.

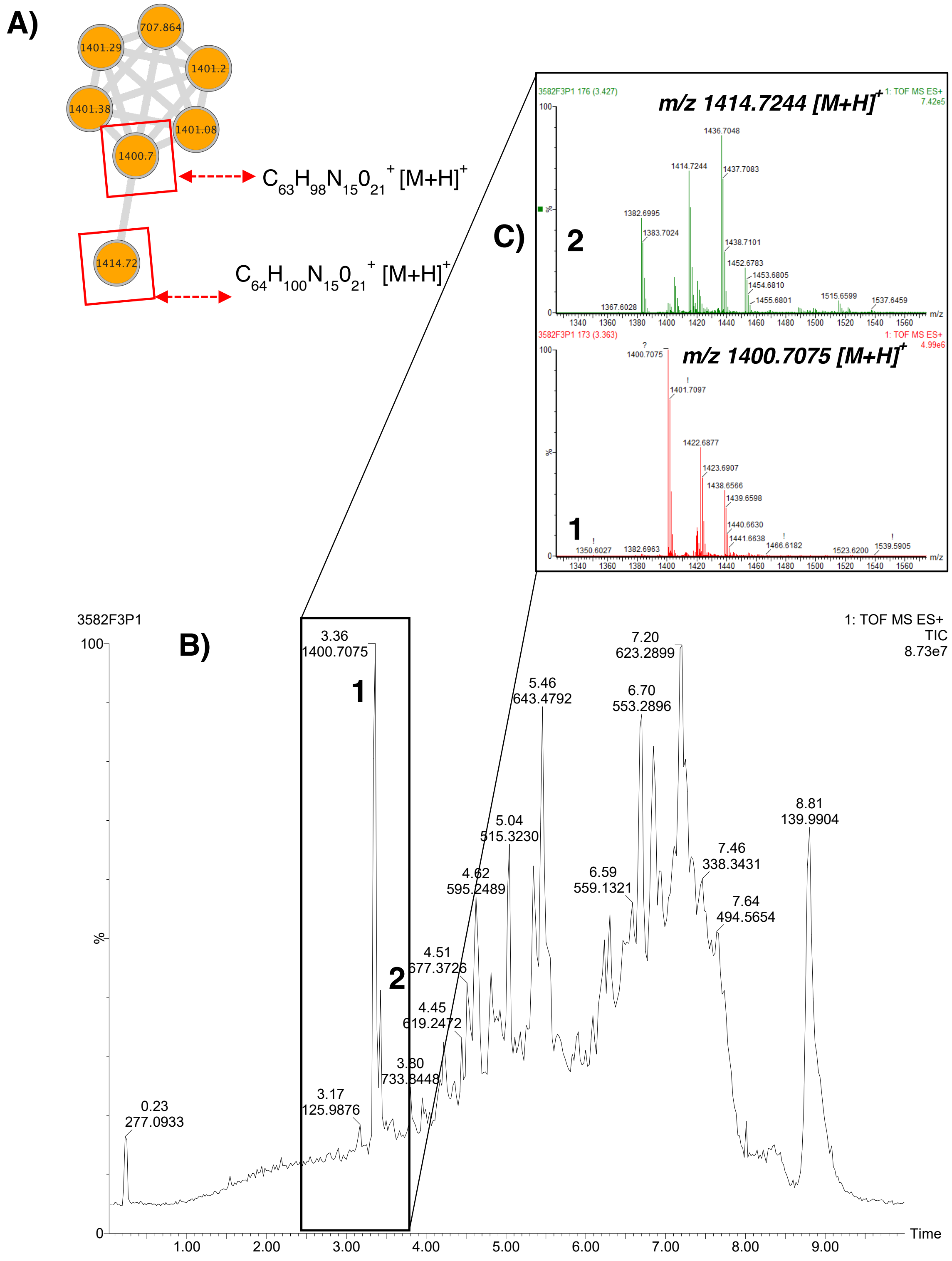

Figure S9. A) GNPS cluster of features from the cyanobacterium *Phormidium* sp. CCIBt3280 highlighting the compounds of interest indicated by NP Analyst as associated with the bioactivity. B) Total ion chromatogram (UHPLC-MS). C) MS1 spectra of the compounds of interest for community 2 indicated by NP Analyst.

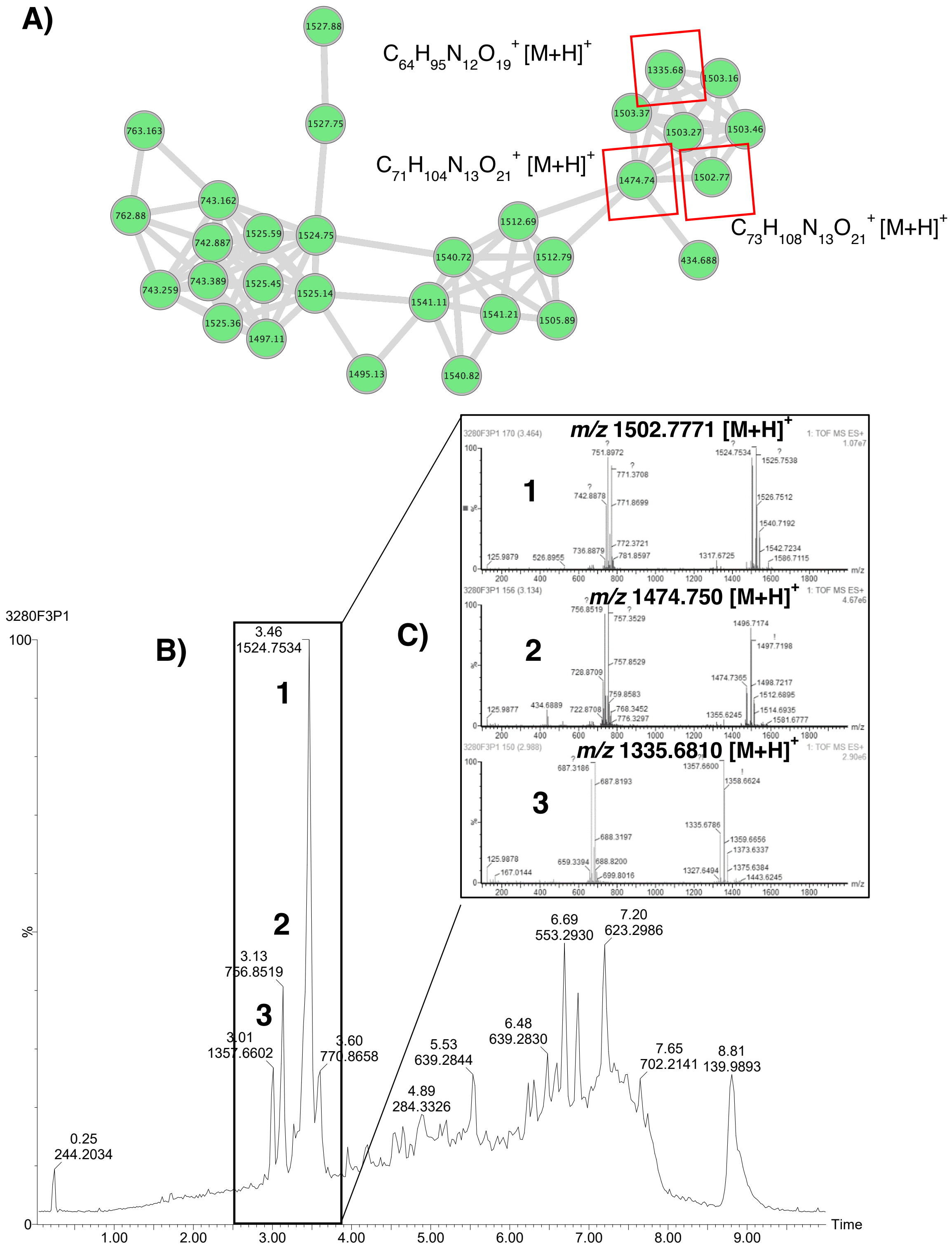

Figure S10. A) GNPS cluster of the cyanobacterium *Calothrix* sp. CCIBt3585 highlighting the compounds of interest indicated by NP Analyst as associated with the bioactivity. B) Total ion chromatogram (UHPLC-MS). C) MS1 spectra of the compounds of interest for community 3 indicated by NP Analyst.

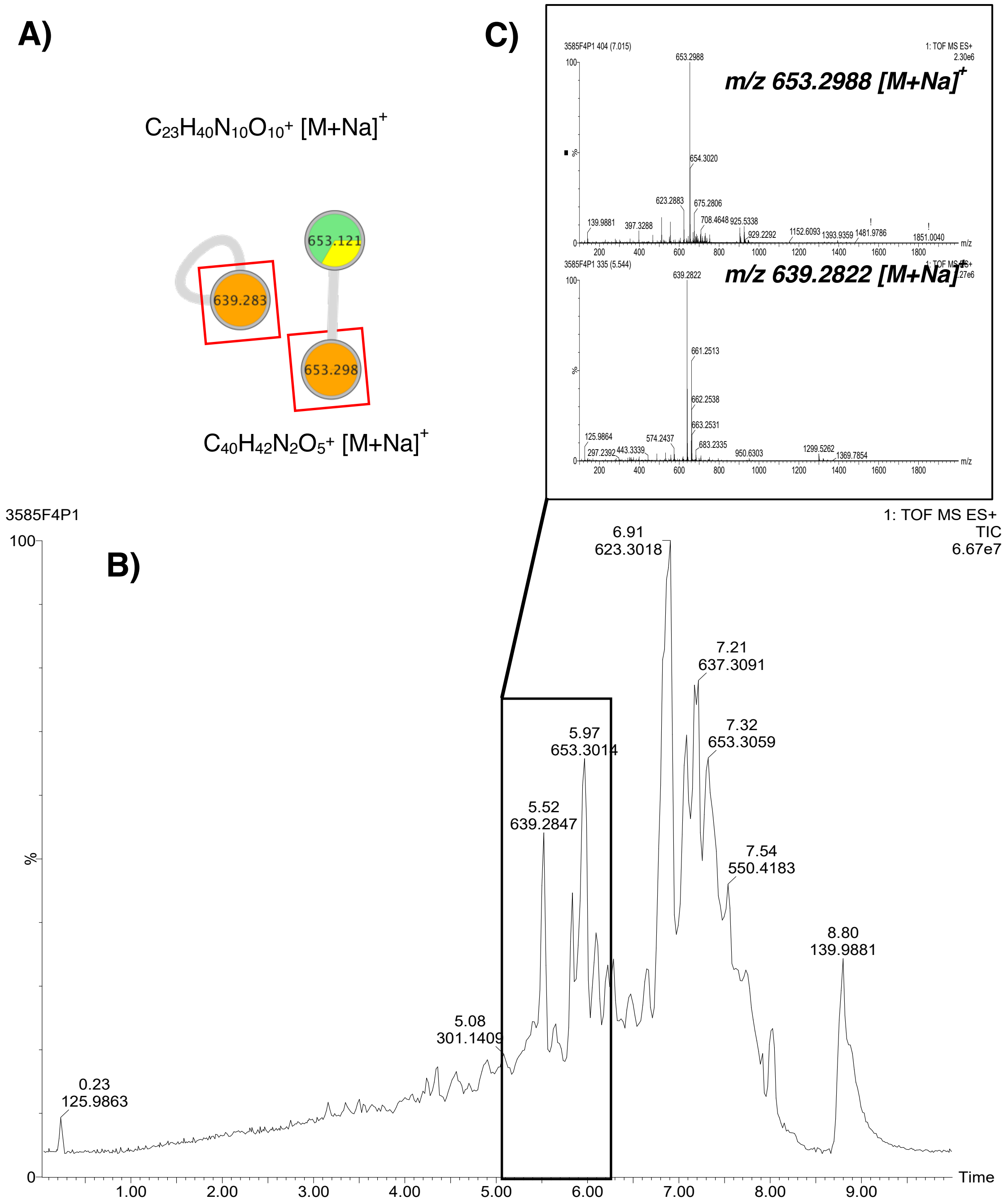

Figure S11. A) GNPS cluster of features from the cyanobacterium *Nostoc* sp. CCIBt3291 highlighting the compounds of interest indicated by NP Analyst as associated with the bioactivity. B) Total ion chromatogram (UHPLC-MS). C) MS1 spectra of one of the compounds of interest from community 4, identified by NP Analyst.

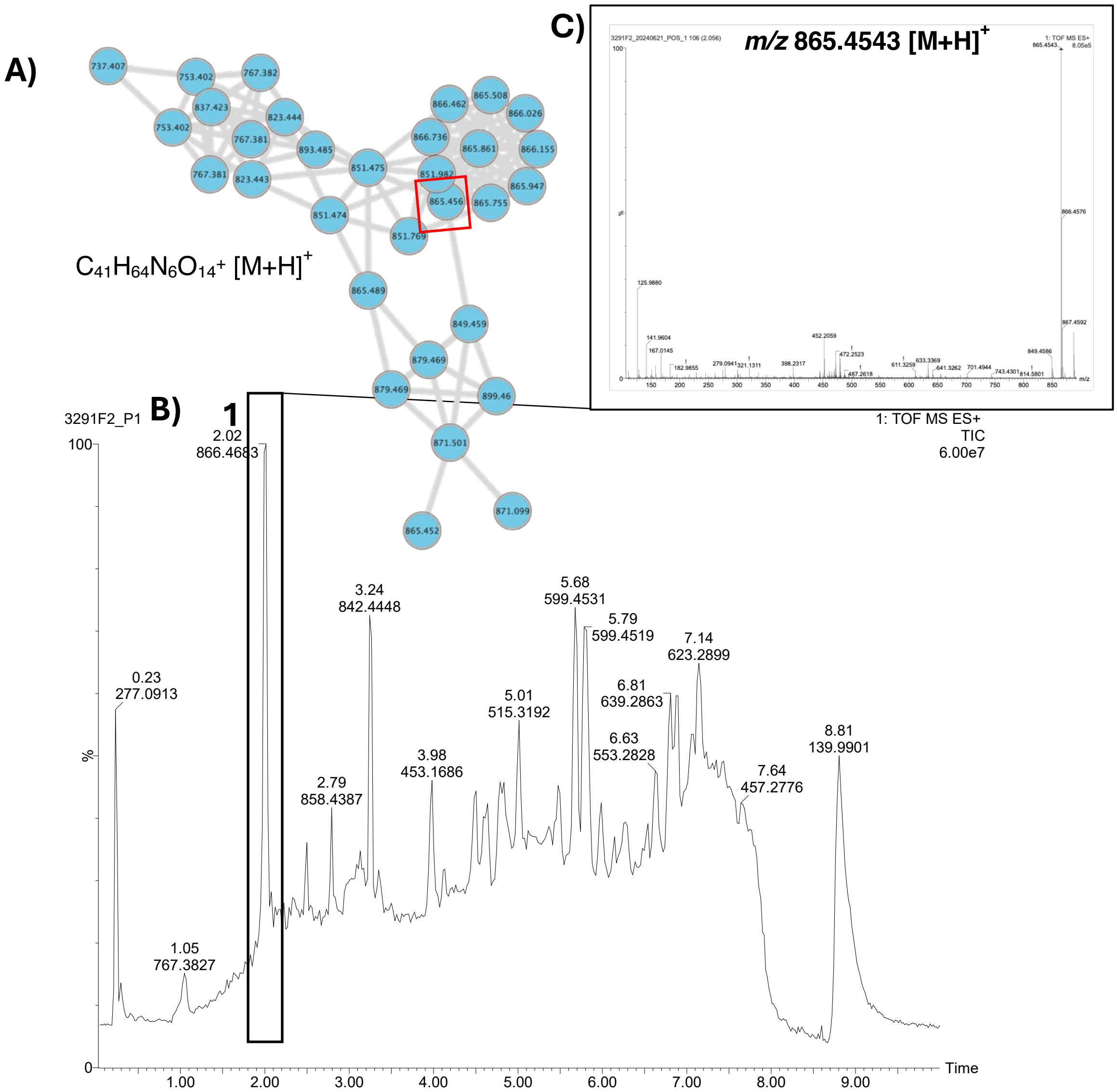

Table S5. Annotation and dereplication of the detected aeruginosins

Compound	Molecular formula	Theorical mass [M+H]	Observed mass [M+H]	RT min	PPM error	SIRIUS Annotation	Activity score	Cluster score
Aeruginosin 736	$C_{35}H_{56}N_6O_{11}$	737.4079	737.4069	1.21	-1.35	$C_{35}H_{56}N_6O_{11}^+$	-	-
Aeruginosin 752	$C_{35}H_{56}N_6O_{12}$	753.4028	753.4033	0.96	+0.66	$C_{35}H_{56}N_6O_{12}^+$	-	-
Aeruginosin 766	$C_{35}H_{54}N_6O_{13}$	767.3821	767.3822	1.06	+0.13	$C_{35}H_{54}N_6O_{13}^+$	-	-
Aeruginosin 822	$C_{39}H_{62}N_6O_{13}$	823.4447	823.4445	1.59	-0.24	$C_{39}H_{62}N_6O_{13}^+$	2.08	0.74
Aeruginosin 836	$C_{39}H_{60}N_6O_{14}$	837.4240	837.4241	1.61	+0.11	$C_{39}H_{60}N_6O_{14}^+$	-	-
Aeruginosin 848	$C_{41}H_{64}N_6O_{13}$	849.4604	849.4600	2.28	-0.47	$C_{41}H_{64}N_6O_{13}^+$	-	-
Aeruginosin 850	$C_{41}H_{66}N_6O_{13}$	851.4760	851.4747	1.97	-1.52	$C_{41}H_{66}N_6O_{13}^+$	2.06	0.76
Aeruginosin 865	$C_{41}H_{64}N_6O_{14}$	865.4553	865.4543	2.05	-1.15	$C_{41}H_{64}N_6O_{14}^+$	2.19	0.87
Aeruginosin 878A	$C_{42}H_{66}N_6O_{14}$	879.4709	879.4697	2.13	-1.36	$C_{42}H_{66}N_6O_{14}^+$	2.08	0.74
Aeruginosin 892	$C_{43}H_{68}N_6O_{14}$	893.4866	893.4852	2.45	-1.56	$C_{43}H_{68}N_6O_{14}^+$	2.19	0.8

Figure S12. A) GNPS cluster of features from the cyanobacterium *Phormidium* sp. CCIBt3095 highlighting the compounds of interest indicated by NP Analyst as associated with the bioactivity. B) Total ion chromatogram (UHPLC-MS). C) MS1 spectra of the compounds of interest from community 5, identified by NP Analyst.

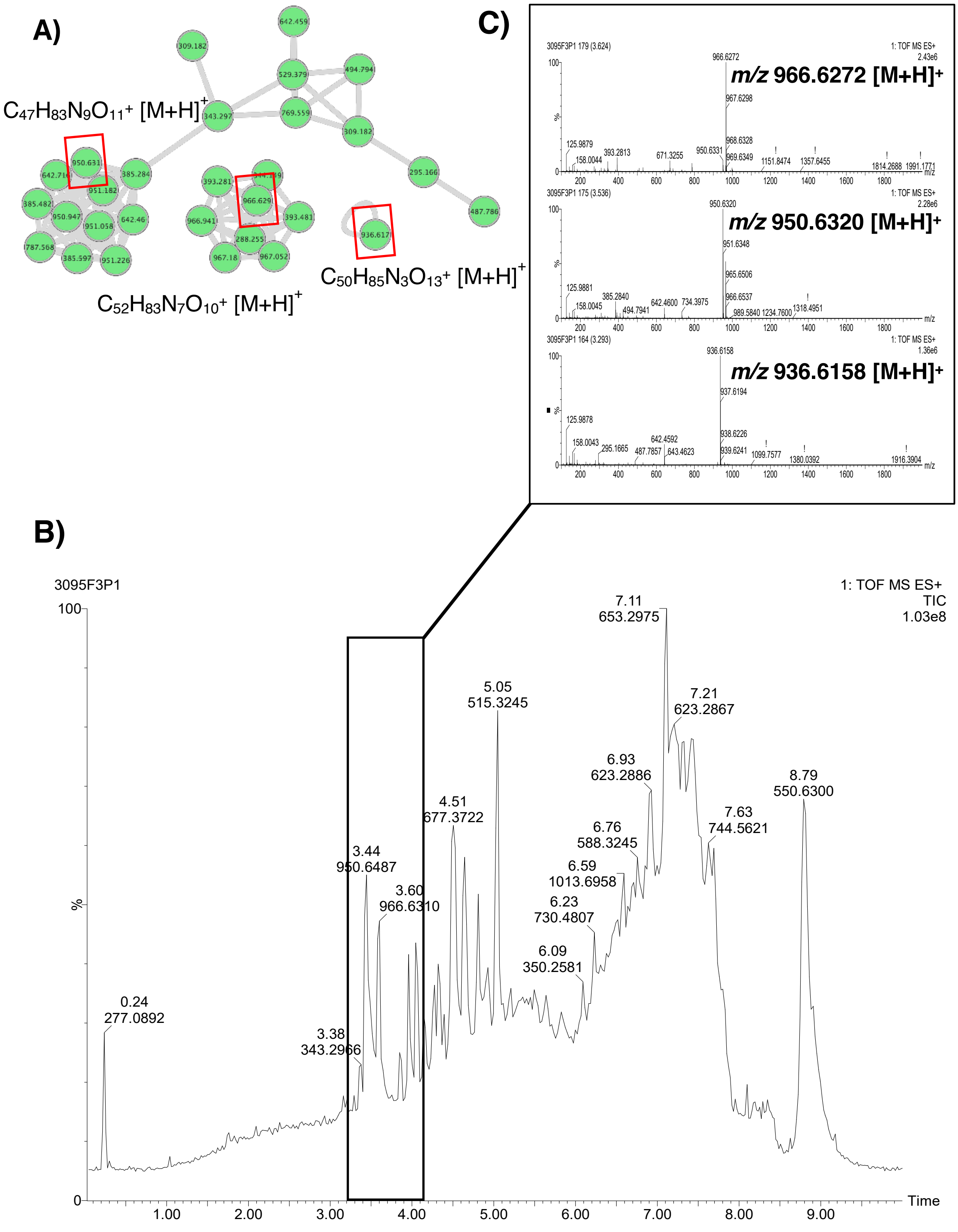

Figure S13. A) GNPS cluster of features from the cyanobacterium *Leptolyngbya* sp. CCIBt3324 highlighting the compounds of interest indicated by NP Analyst as as associated with the bioactivity. B) Total ion chromatogram (UHPLC-MS). C) MS1 spectra of the compounds of interest from community 6, identified by NP Analyst.

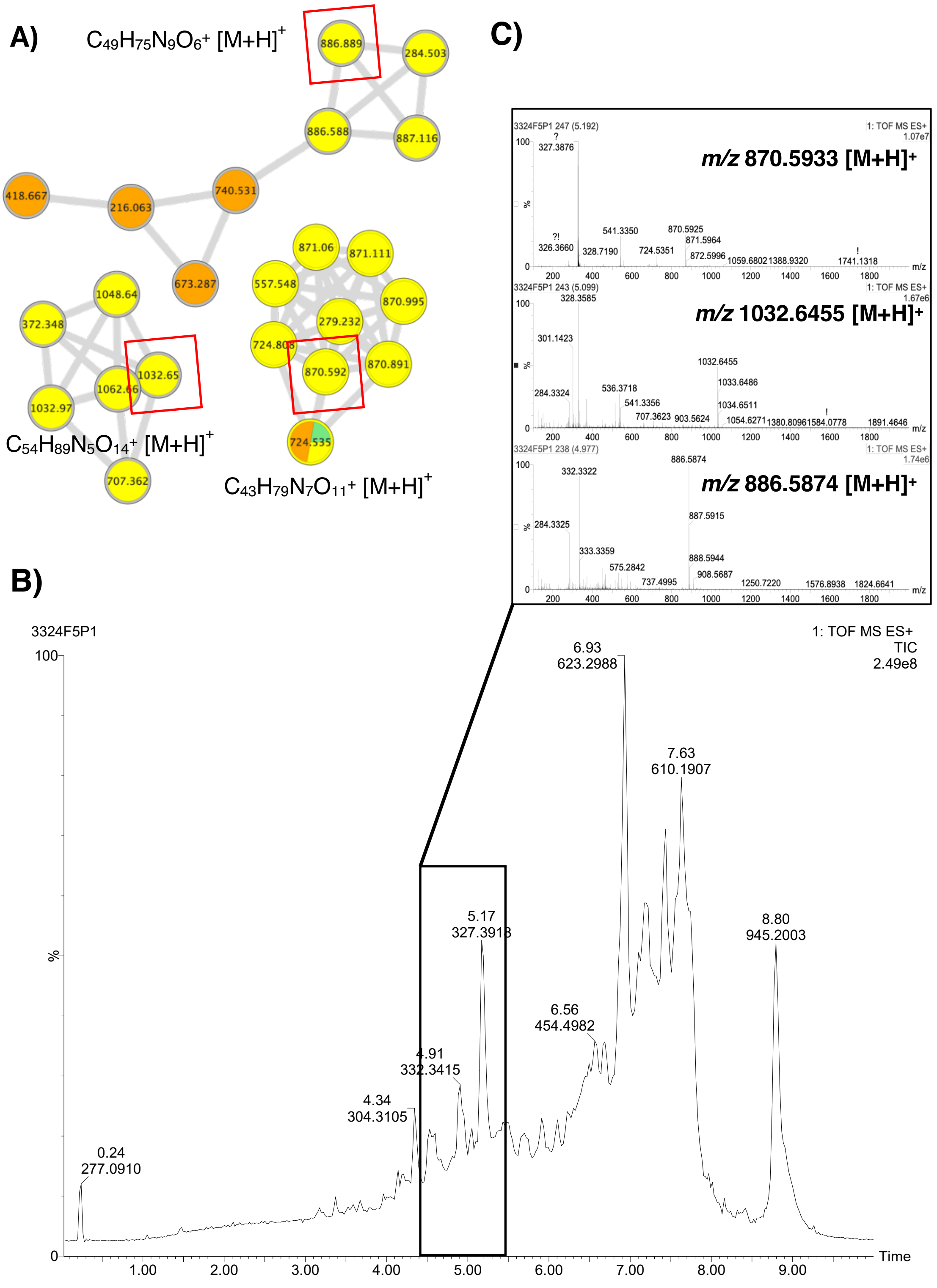

Figure S14. A) GNPS cluster of features from the cyanobacterium *Phormidium* sp. CCIBt3278 highlighting the compounds of interest indicated by NP Analyst associated with the bioactivity. B) Total ion chromatogram (UHPLC-MS). C) MS1 spectra of the compounds of interest from community 7, identified by NP Analyst.

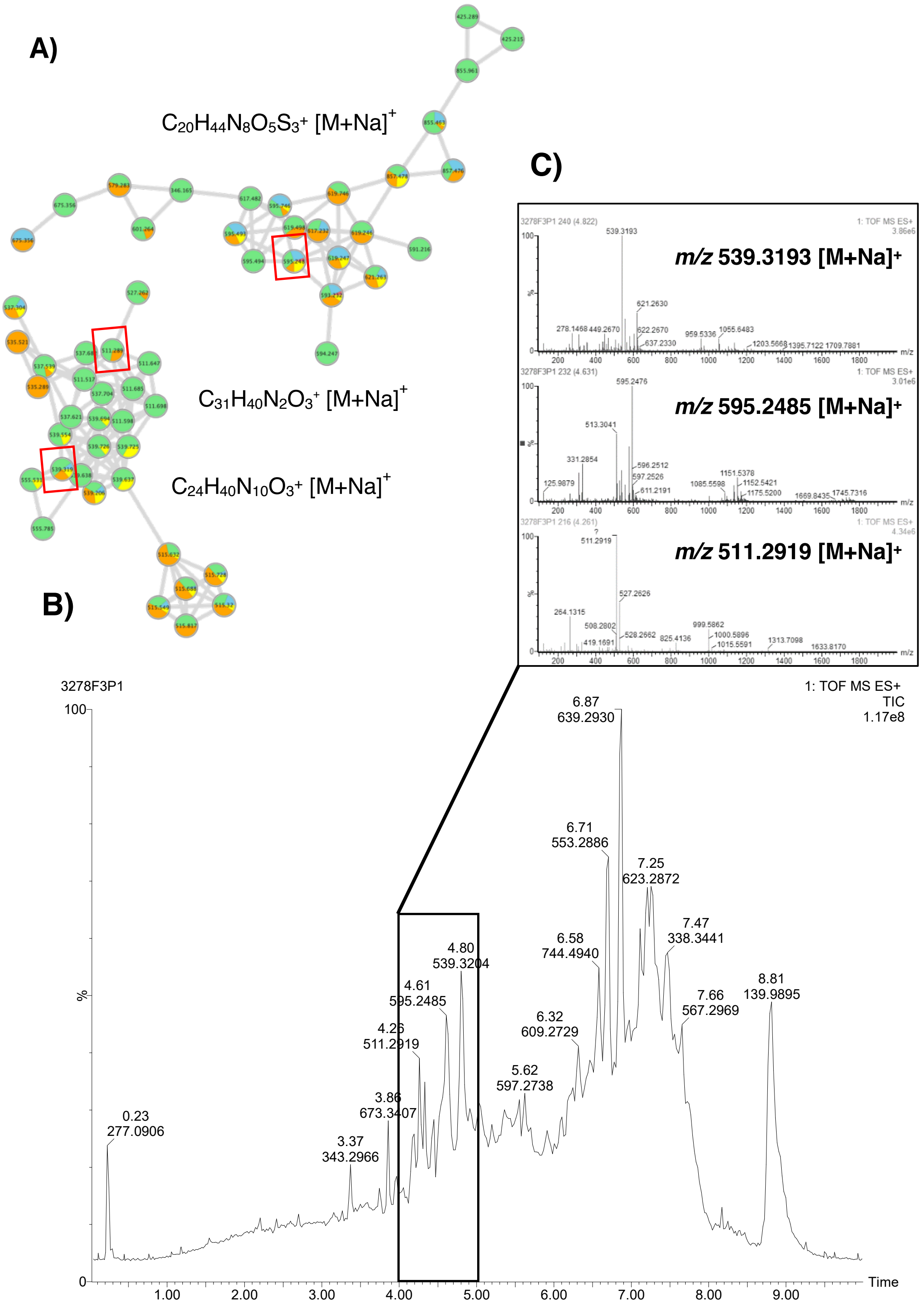

Figure S15. A) GNPS cluster of features from the cyanobacterium *Nostoc* sp. CCIBt3248 highlighting the compounds of interest indicated by NP Analyst as as associated with the bioactivity. B) Total ion chromatogram (UHPLC-MS). C) MS1 spectra of the compounds of interest from community 8, identified by NP Analyst.

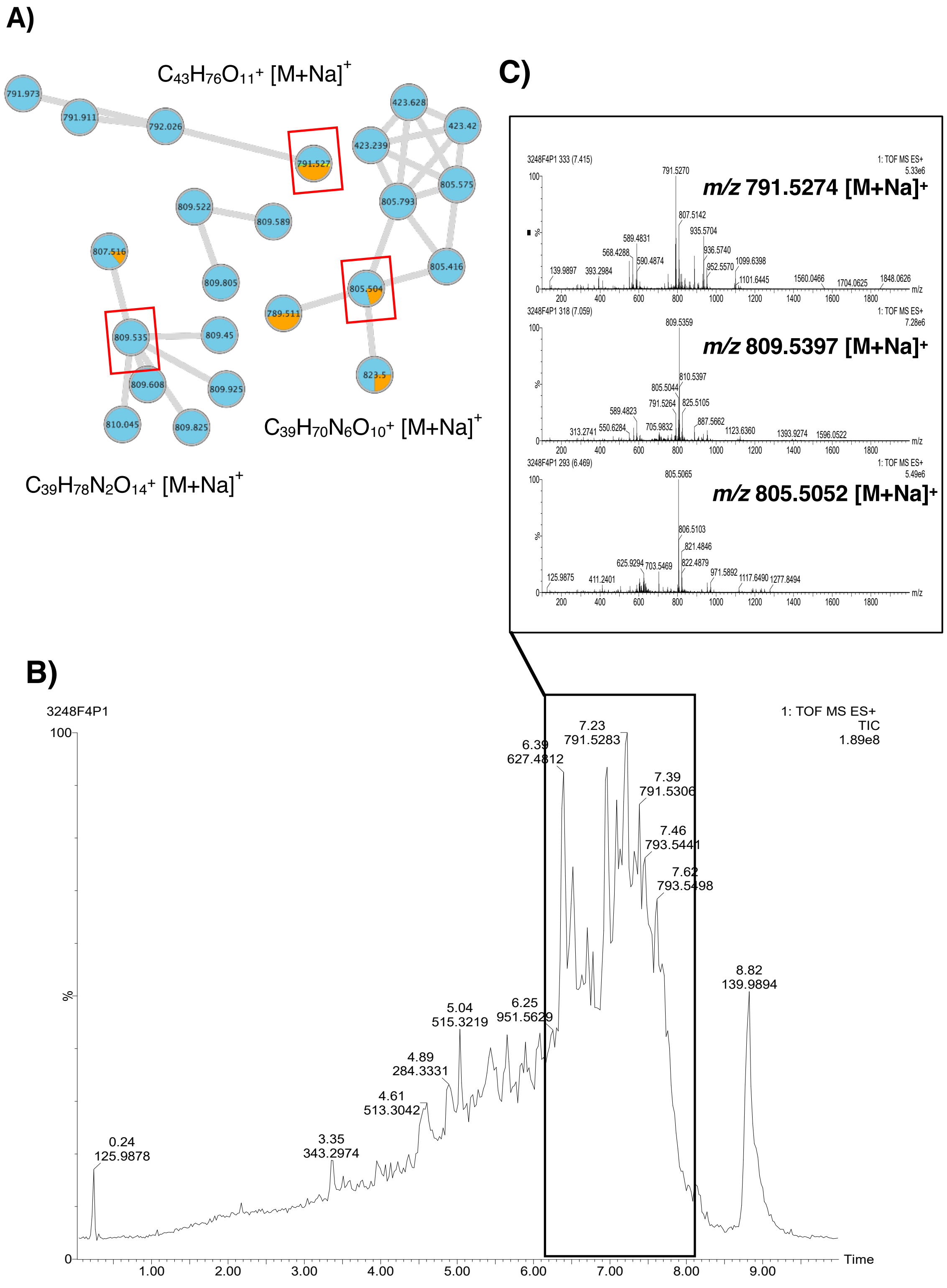

Table S6. Annotation of the features highlighted by NP Analyst and dereplicated by SIRIUS.

NP Analyst	Observed mass	Íon	RT (min)	SIRIUS annotation	Exact mass	PPM error	Activity score	Cluster score
**Community 1**								
Compound 1	1400.7075	$[M+H]^+$	3.31	$C_{63}H_{98}N_{15}O_{21}^+$	1400.7056	+1.35	2.26	0.53
Compound 2	1414.7234	$[M+H]^+$	3.43	$C_{64}H_{100}N_{15}O_{21}^+$	1414.7212	+1.55	3.31	0.54
**Community 2**								
Compound 3	1335.6810	$[M+H]^+$	3.01	$C_{64}H_{95}N_{12}O_{19}^+$	1335.6831	-1.57	4.22	0.83
Compound 4	1474.7450	$[M+H]^+$	3.13	$C_{71}H_{104}N_{13}O_{21}^+$	1474.7464	-0.94	4.22	0.83
Compound 5	1502.7771	$[M+H]^+$	3.43	$C_{73}H_{108}N_{13}O_{21}^+$	1502.7777	-0.39	2.53	0.65
**Community 3**								
Compound 6	639.2822	$[M+Na]^+$	5.52	$C_{23}H_{40}N_{10}O_{10}^+$	639.2821	+0.15	2.11	0.53
Compound 7	653.2988	$[M+Na]^+$	5.83	$C_{40}H_{42}N_2O_5^+$	653.2985	+0.45	2.36	0.66
**Community 4**								
Compound 8	871.5018	$[M+H]^+$	1.96	$C_{41}H_{70}N_6O_{14}^+$	871.5022	-0.45	-	-
Compound 9	899.4602	$[M+H]^+$	1.88	$C_{41}H_{66}N_6O_{16}^+$	899.4608	-0.66	2.19	0.87
**Community 5**								
Compound 10	936.6158	$[M+H]^+$	3.29	$C_{50}H_{85}N_3O_{13}^+$	936.6155	+0.32	-	-
Compound 11	966.6272	$[M+H]^+$	3.62	$C_{52}H_{83}N_7O_{10}^+$	966.6274	-0.20	-	-
Compound 12	950.6320	$[M+H]^+$	3.53	$C_{47}H_{83}N_9O_{11}^+$	950.6284	+3.78	2.47	0.7
**Community 6**								
Compound 13	886.5874	$[M+H]^+$	4.97	$C_{49}H_{75}N_9O_6^+$	886.5913	-4.39	4.44	0.81
Compound 14	1032.6455	$[M+H]^+$	5.09	$C_{54}H_{89}N_5O_{14}^+$	1032.6478	-2.22	-	-
Compound15	870.5933	$[M+H]^+$	5.23	$C_{43}H_{79}N_7O_{11}^+$	870.5910	+2.64	4.44	0.81
**Community 7**								
Compound 16	511.2919	$[M+Na]^+$	4.26	$C_{31}H_{40}N_2O_3^+$	511.2931	-2.34	3.14	0.81
Compound 17	539.3193	$[M+Na]^+$	4.82	$C_{24}H_{40}N_{10}O_3^+$	539.3177	+2.96	2.31	0.6
Compound 18	595.2485	$[M+Na]^+$	4.63	$C_{20}H_{44}N_8O_5S_3$	595.2489	-0.67	-	-
**Community 8**								
Compound 19	791.5274	$[M+Na]^+$	7.41	$C_{43}H_{76}O_{11}^+$	791.5279	-0.63	2.06	0.93
Compound 20	805.5052	$[M+Na]^+$	4.46	$C_{39}H_{70}N_6O_{10}^+$	805.5045	+0.86	2.06	0.93
Compound 21	809.5359	$[M+Na]^+$	7.05	$C_{38}H_{78}N_2O_{14}^+$	809.5345	+1.72	-	-

Figure S16. Comparison between anabaenopeptin B and F standards and fraction F2 from *Calothrix* sp. CCIBt3581. a = Analysis of anabaenopeptin standards by HPLC-UV-MS. a.1 = anabaenopeptin B standard. a.2 = anabaenopeptin F standard. b = Spiking of anabaenopeptin standards into fraction 2. b.1 = Anabaenopeptin B + standard. b.2 = anabaenopeptin F + standard. c = Fraction 2 detecting anabaenopeptins B and F. c.1 = anabaenopeptin B. c.2 = Anabaenopeptin F. d = Total ion chromatogram of fraction 2 analyzed by UHPLC-MS/MS. d.1 = Peak corresponding to anabaenopeptin B. d.2 = Peak corresponding to anabaenopeptin F.

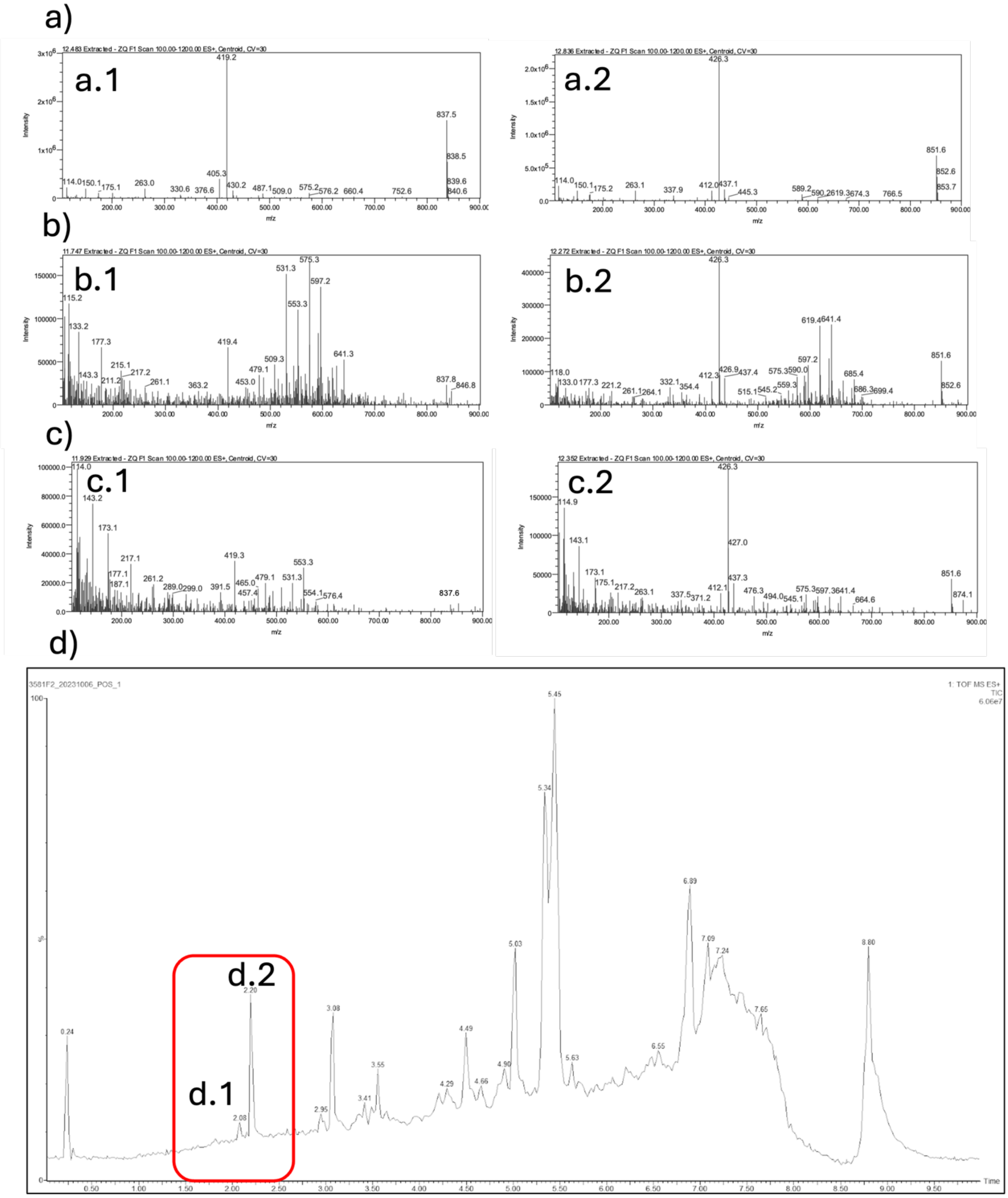

Figure S17. MS data of anabaenopeptin B. (A) MS1 spectrum. (B) MS/MS spectrum with annotated fragment ions

A)

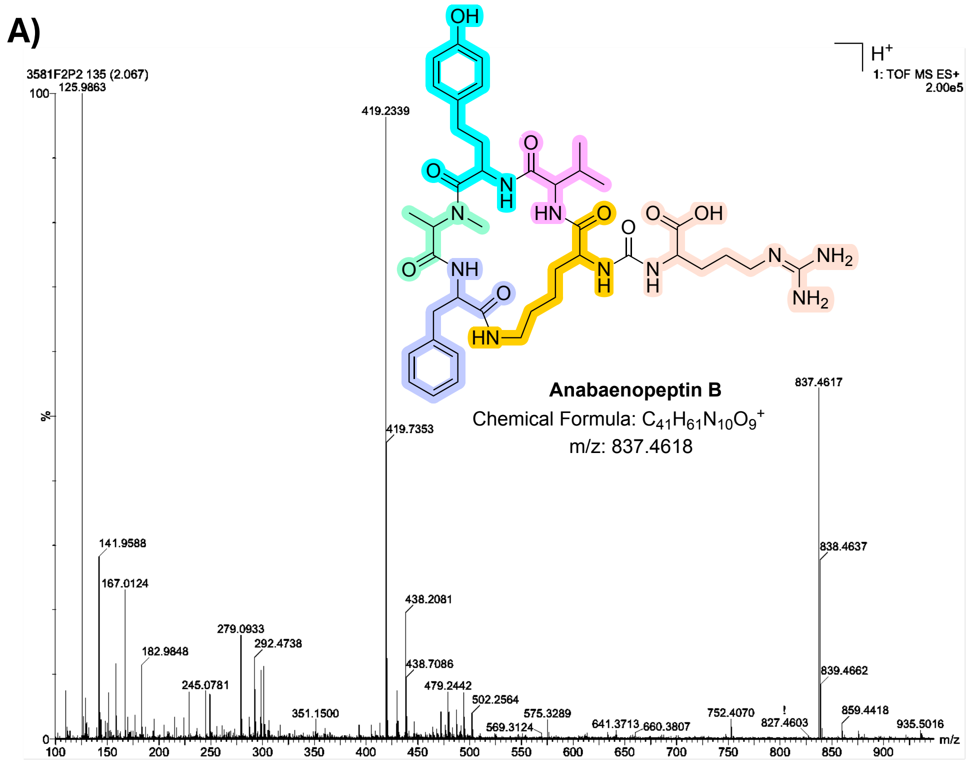

B)

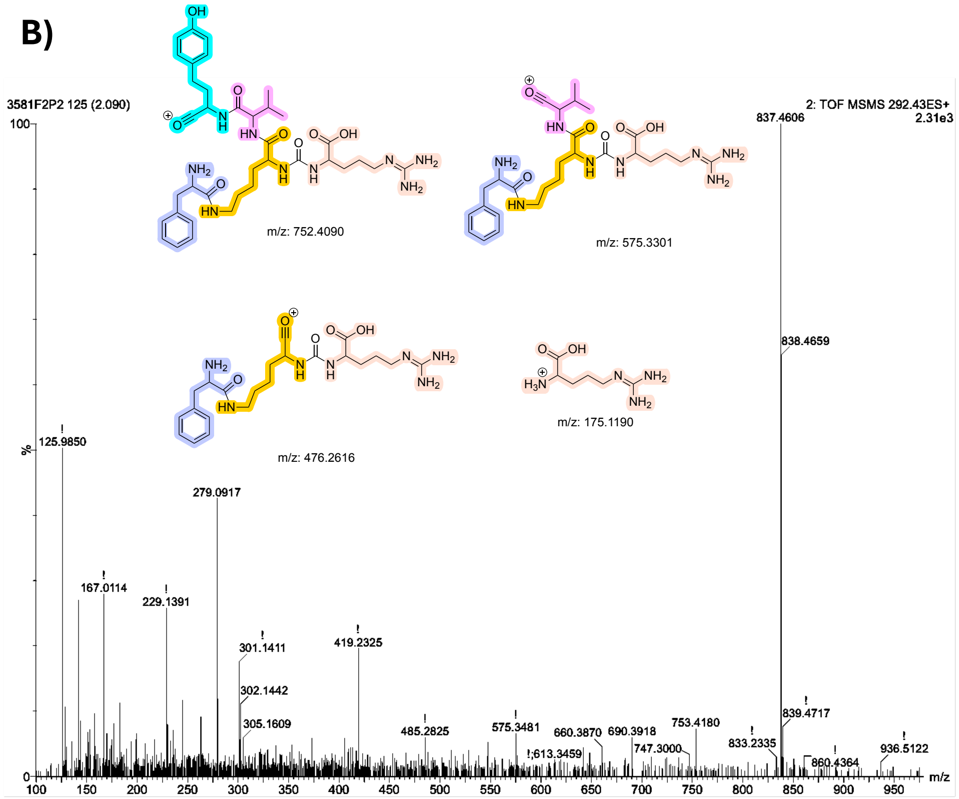

Figure S18. MS data of anabaenopeptin F. A) MS1 spectrum. B) MS/MS spectrum with annotated fragment ions

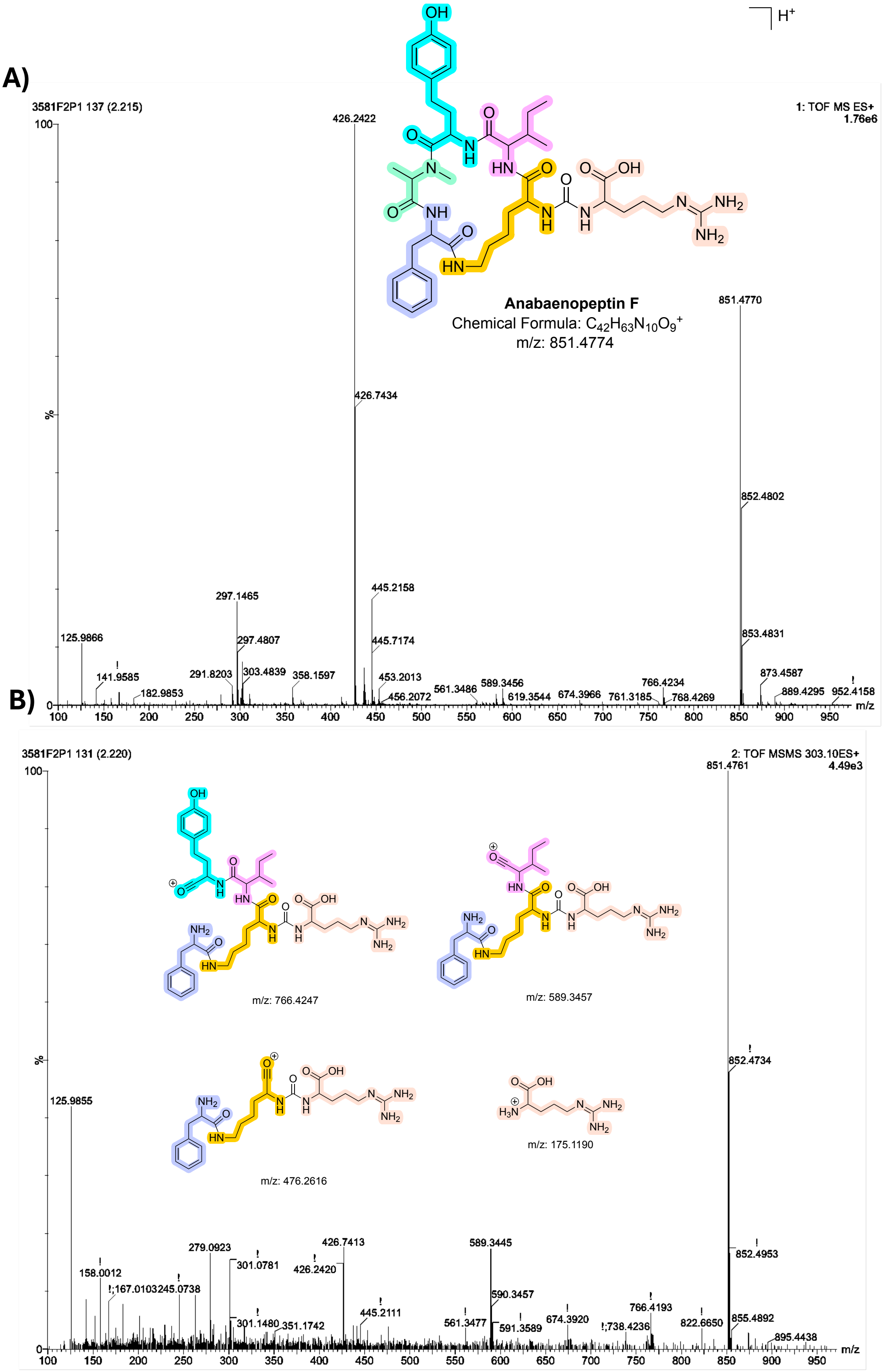

Figure S19. MS data of anabaenopeptin J and SIRIUS annotation. A) MS1 spectrum. B) MS/MS spectrum. C) SIRIUS annotation

A)

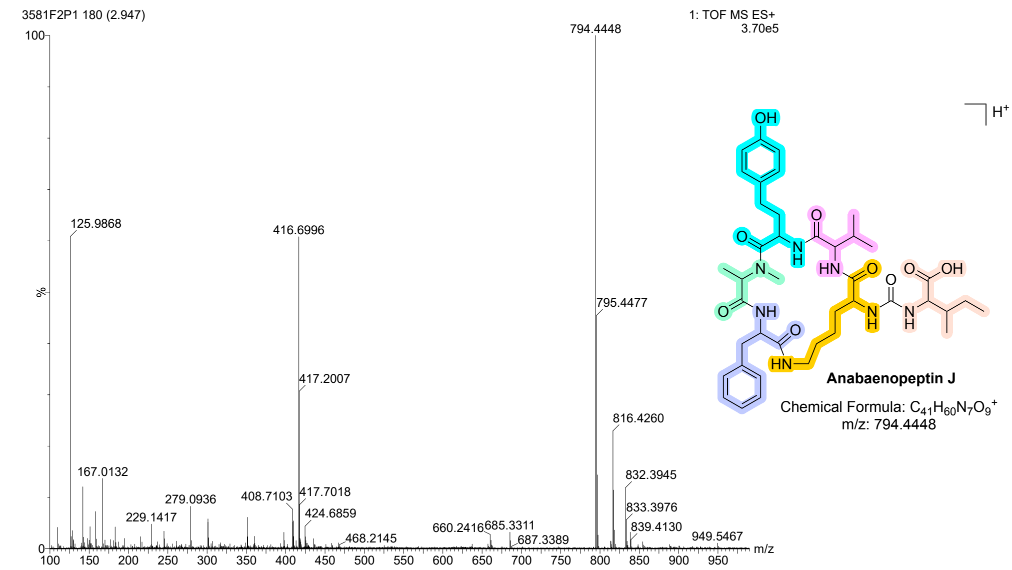

B)

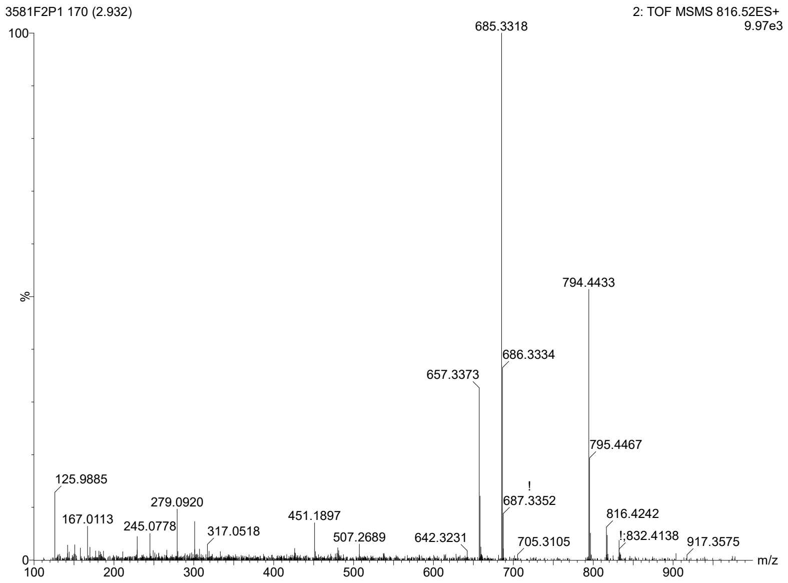

C)

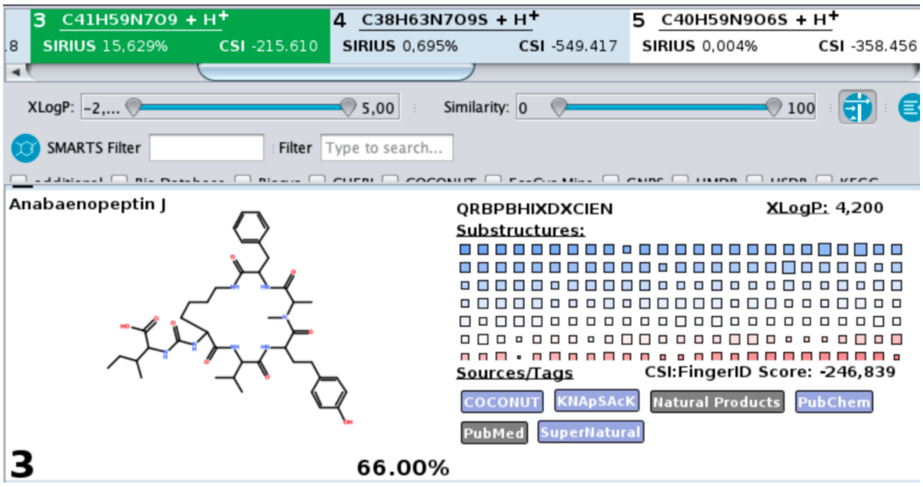

Figure S20. MS data of the anabaenopeptin 807 and SIRIUS annotation. A) MS1 spectrum. B) MS/MS spectrum.

A)

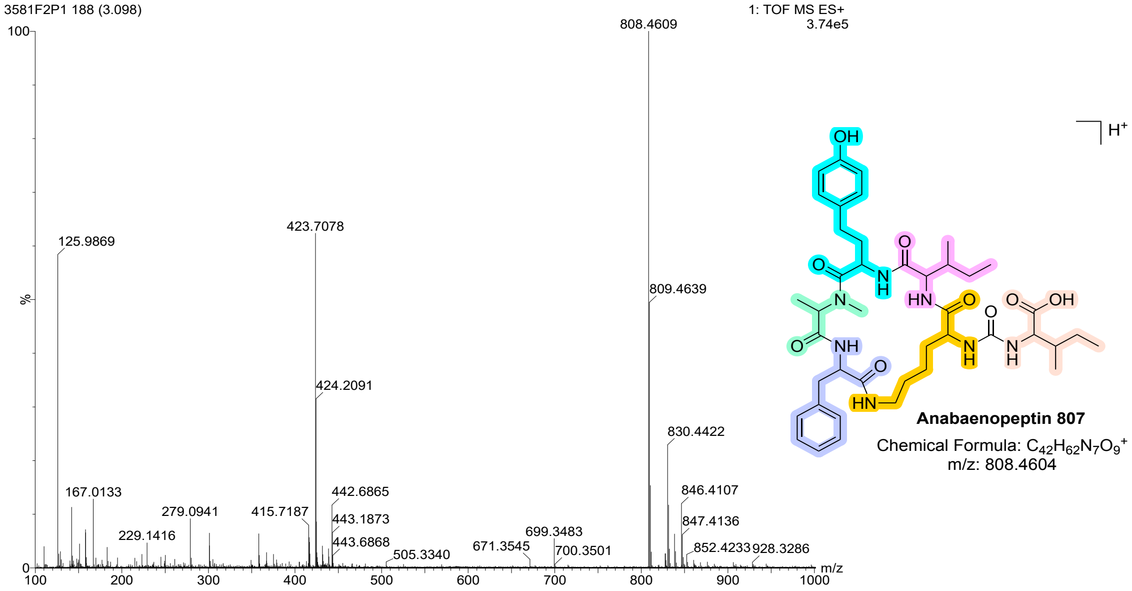

B)

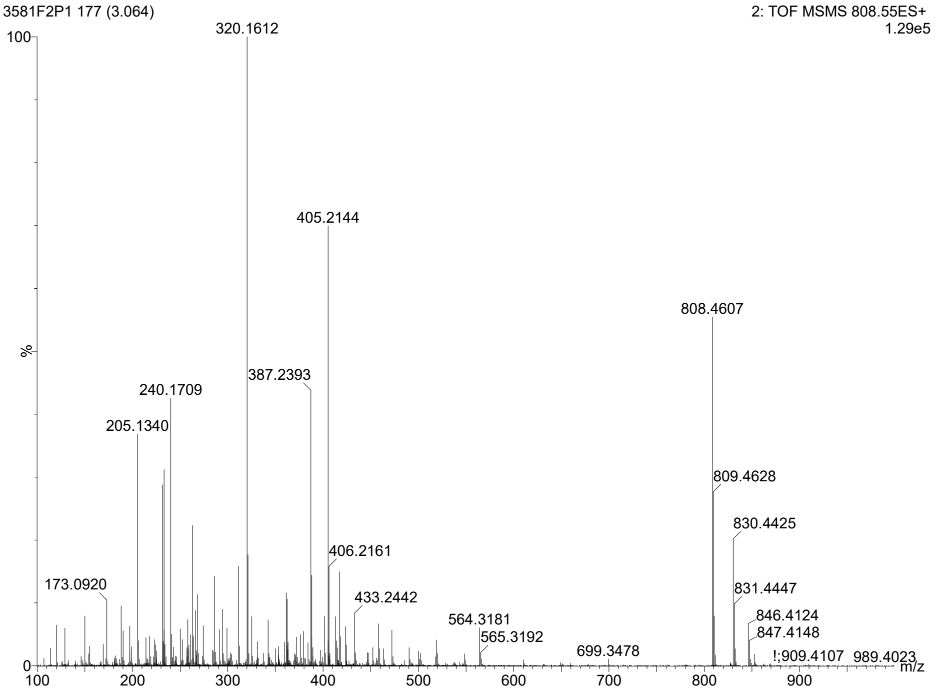

Figure S21. MS data of lyngbyaureidamide B and SIRIUS annotation. A) MS1 spectrum. B) MS/MS spectrum. C) SIRIUS annotation.

A)

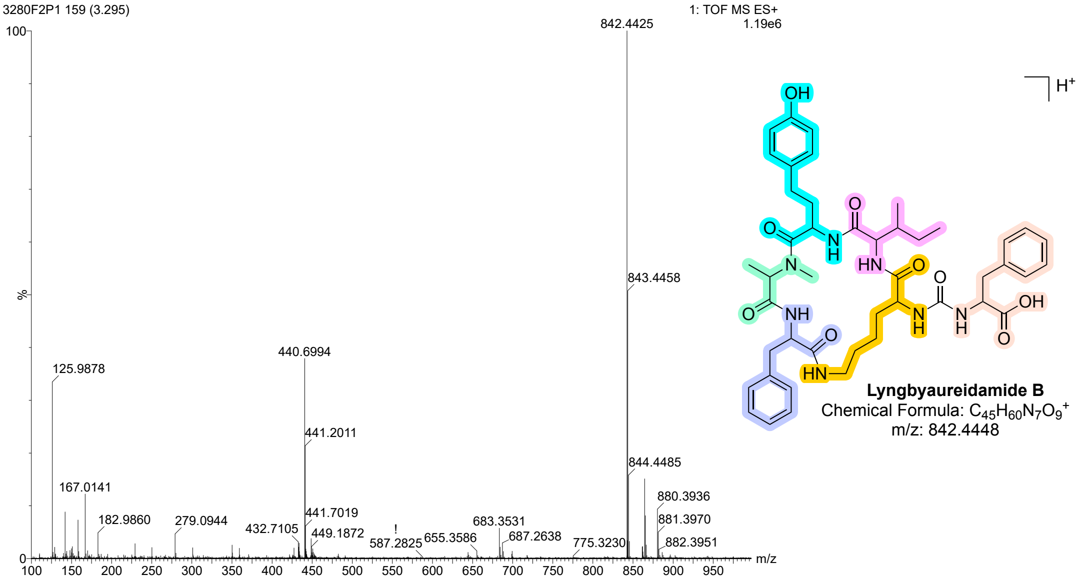

B)

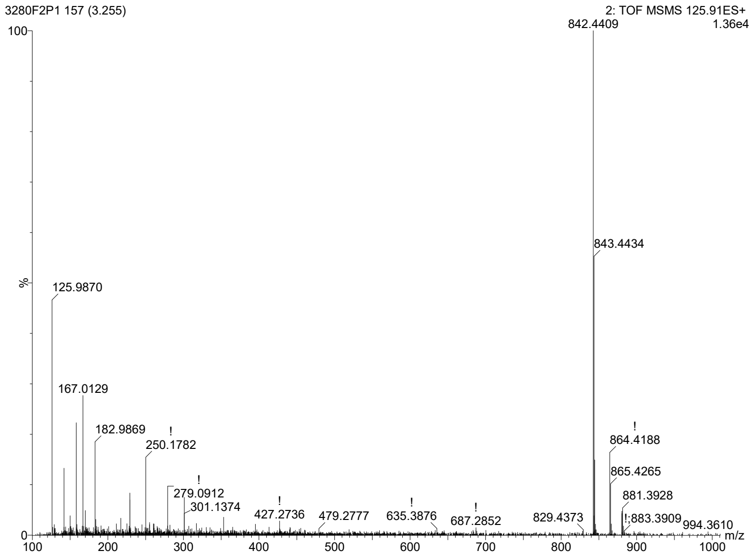

C)

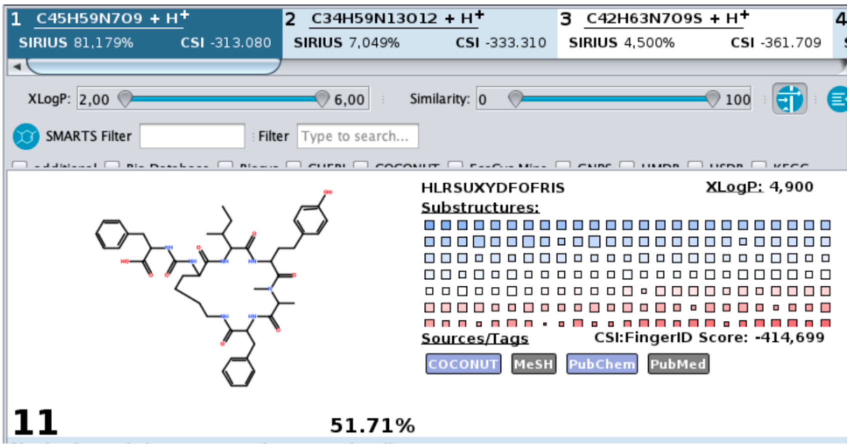

Figure S22. MS data of oscillamide Y and SIRIUS annotation. A) MS1 spectrum. B) MS/MS spectrum. C) SIRIUS annotation.

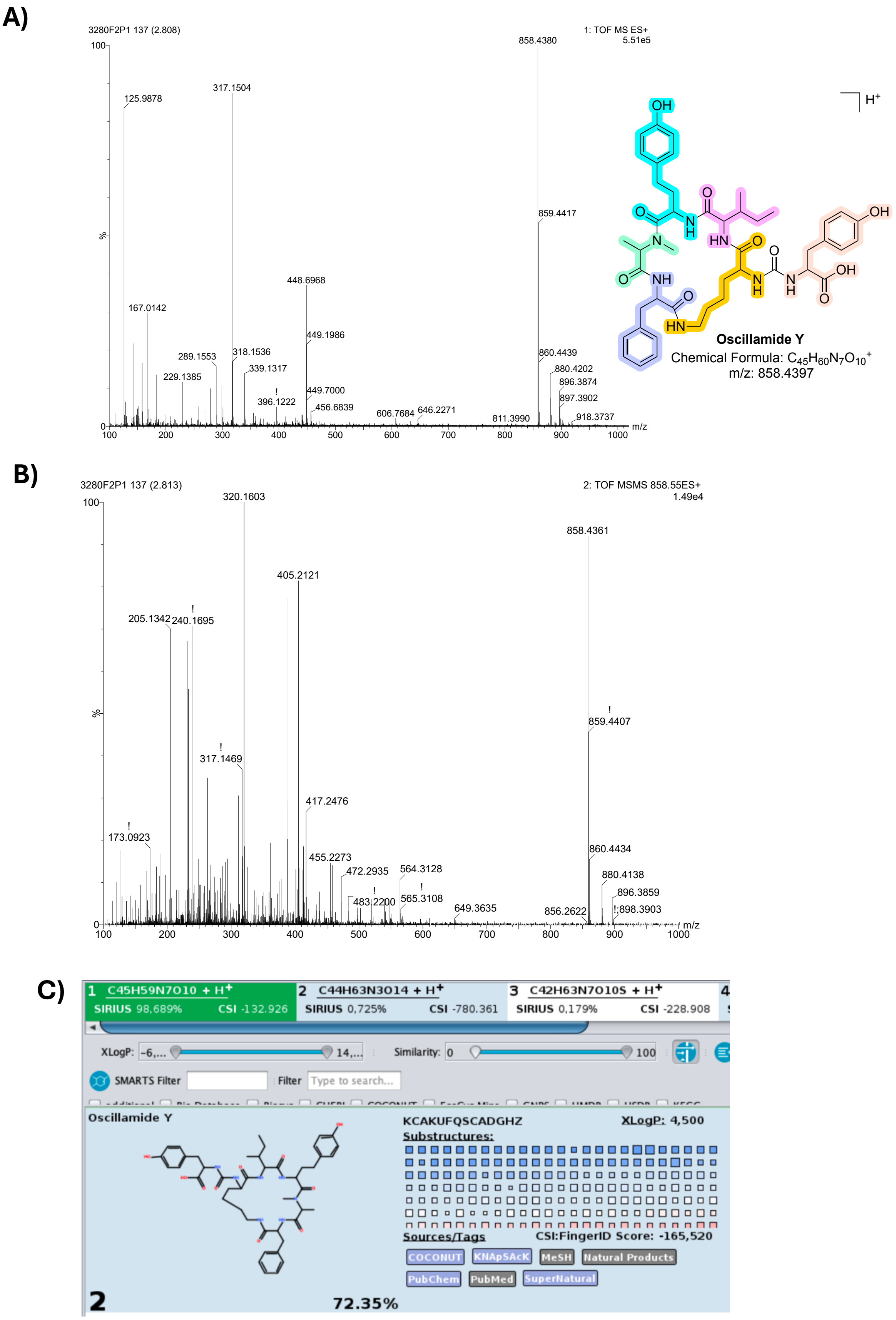

Figure S23. MS data of anabaenopeptin NZ841 and SIRIUS annotation. A) MS1 spectrum. B) MS/MS spectrum. C) SIRIUS annotation.

A)

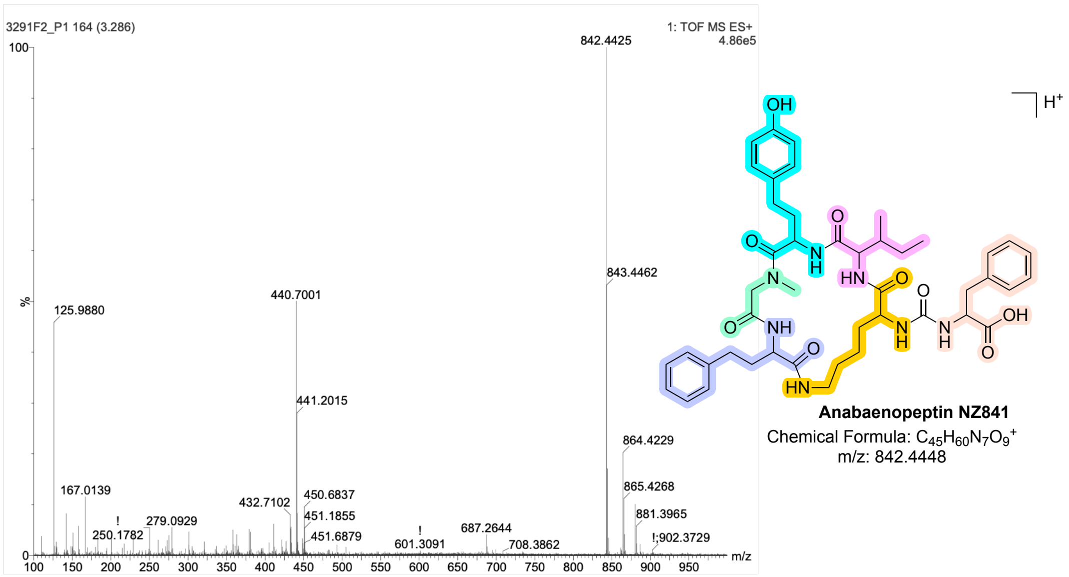

B)

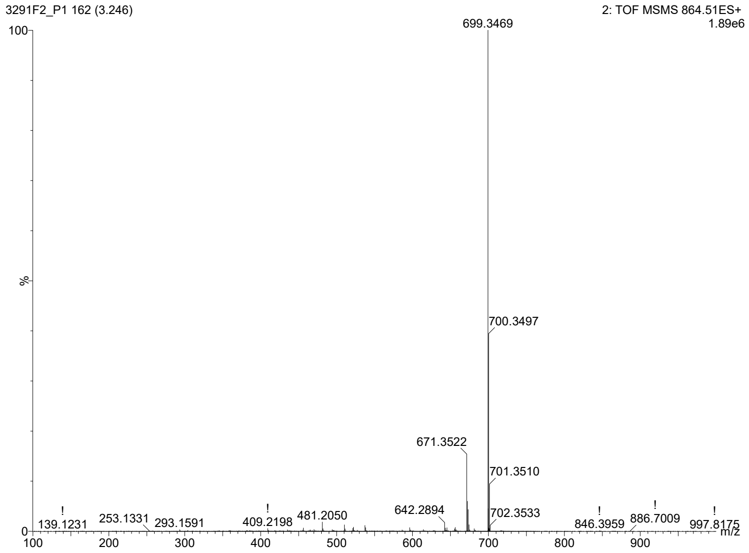

C)

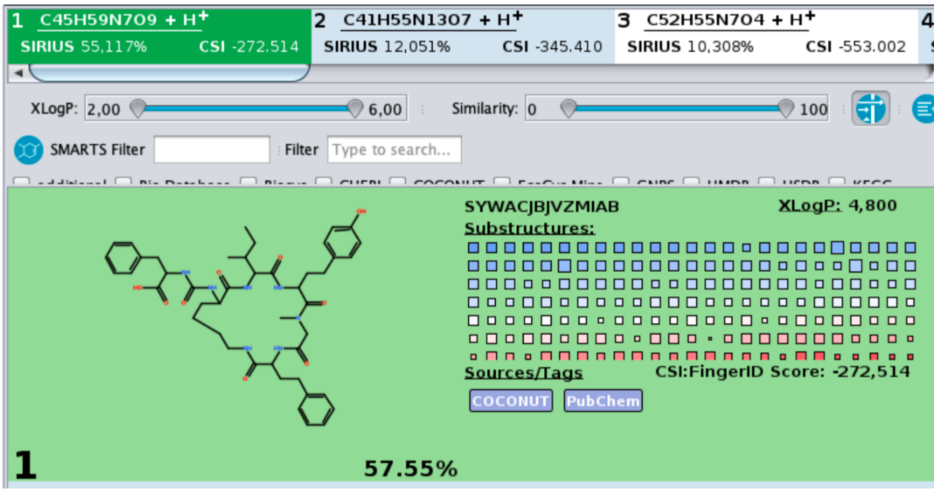

Figure S24. MS data of oscillamide Y and SIRIUS annotation. A) MS1 spectrum. B) MS/MS spectrum. C) SIRIUS annotation.

A)

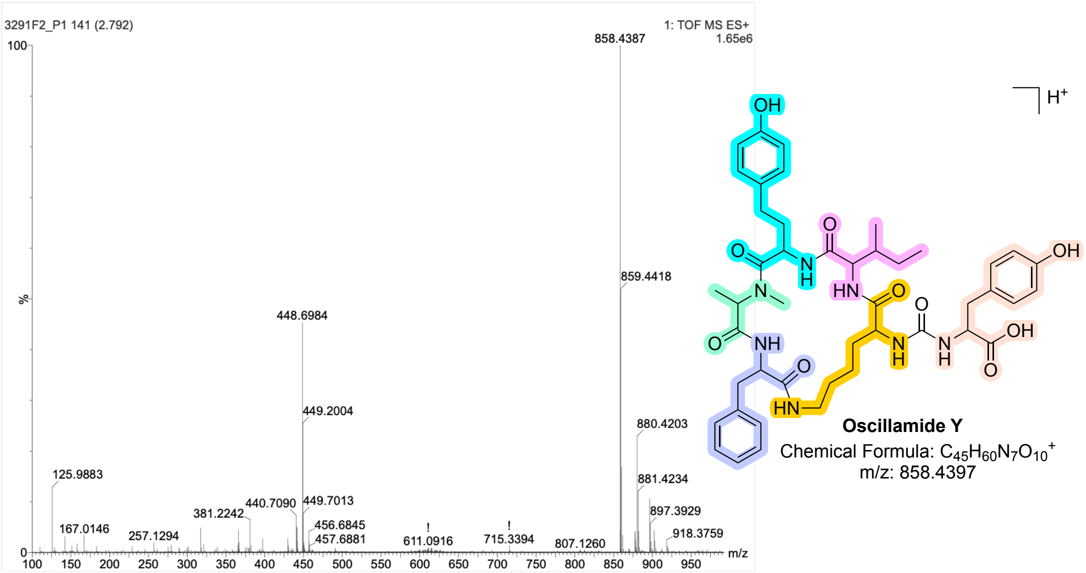

B)

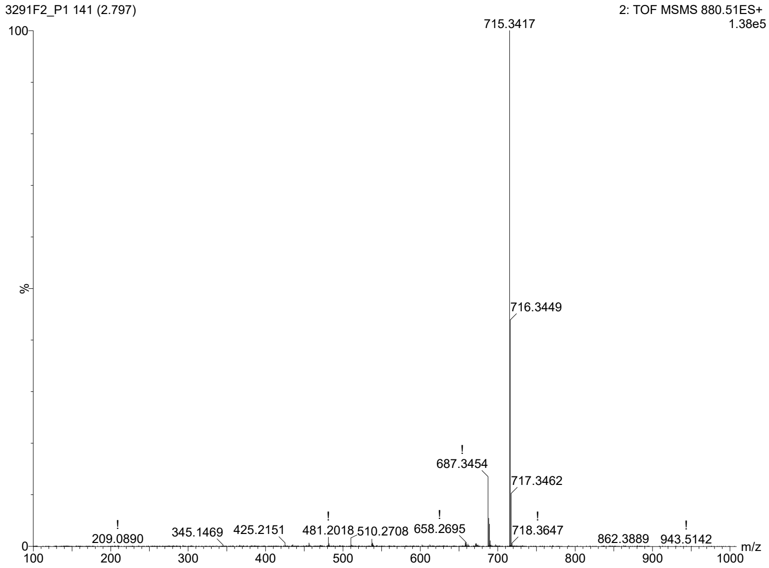

C)

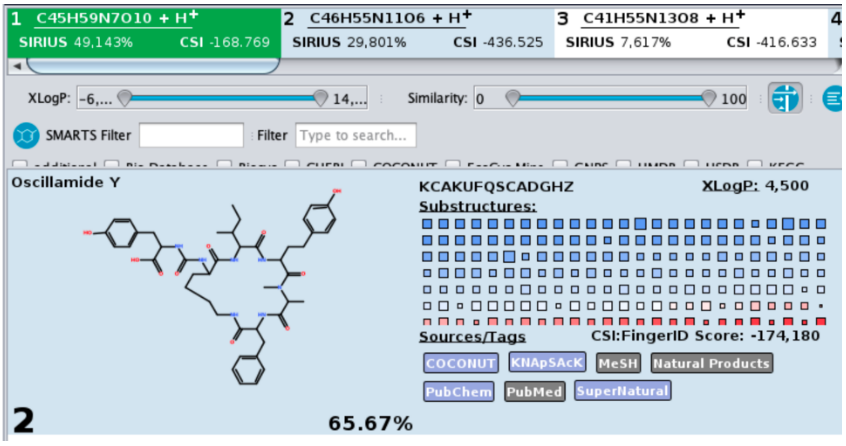

Figure S25. MS data of lyngbyaureidamide A and SIRIUS annotation. A) MS1 spectrum. B) MS/MS spectrum. C) SIRIUS annotation.

A)

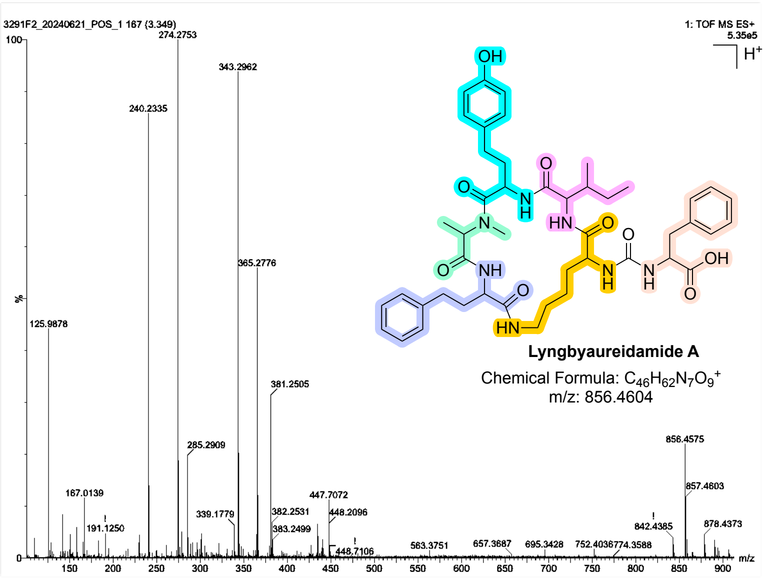

B)

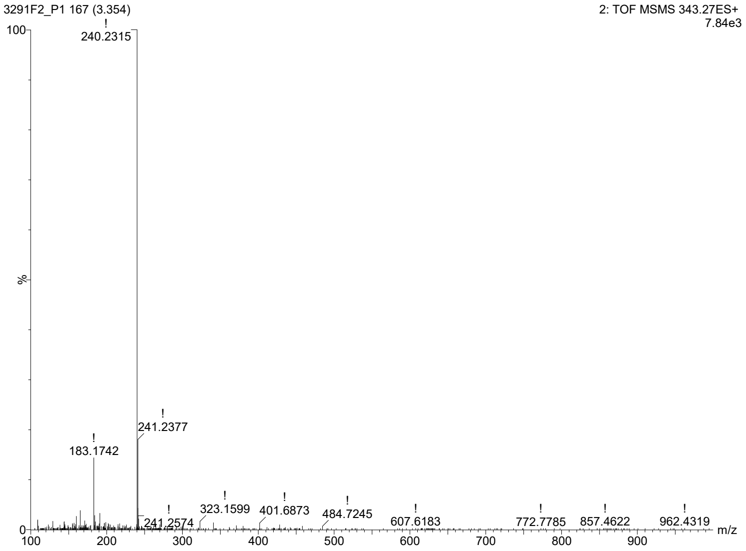

C)

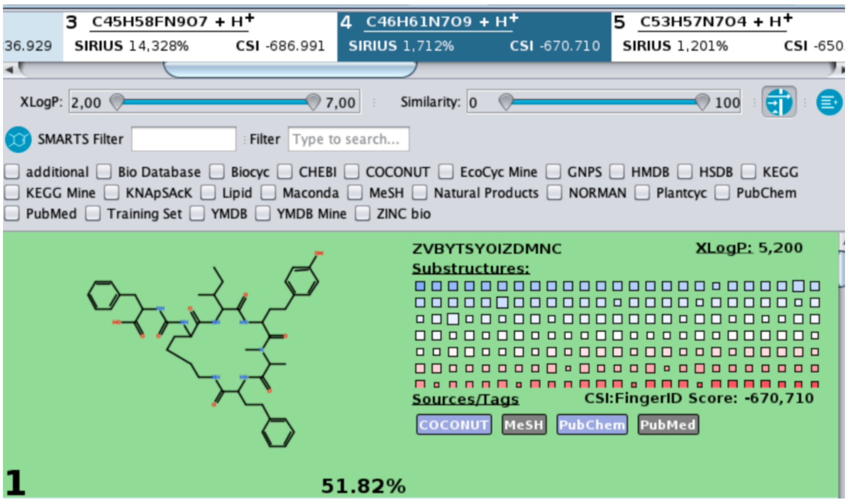

Figure S26. MS/MS spectrum and fragment ion annotation of aeruginosin 736

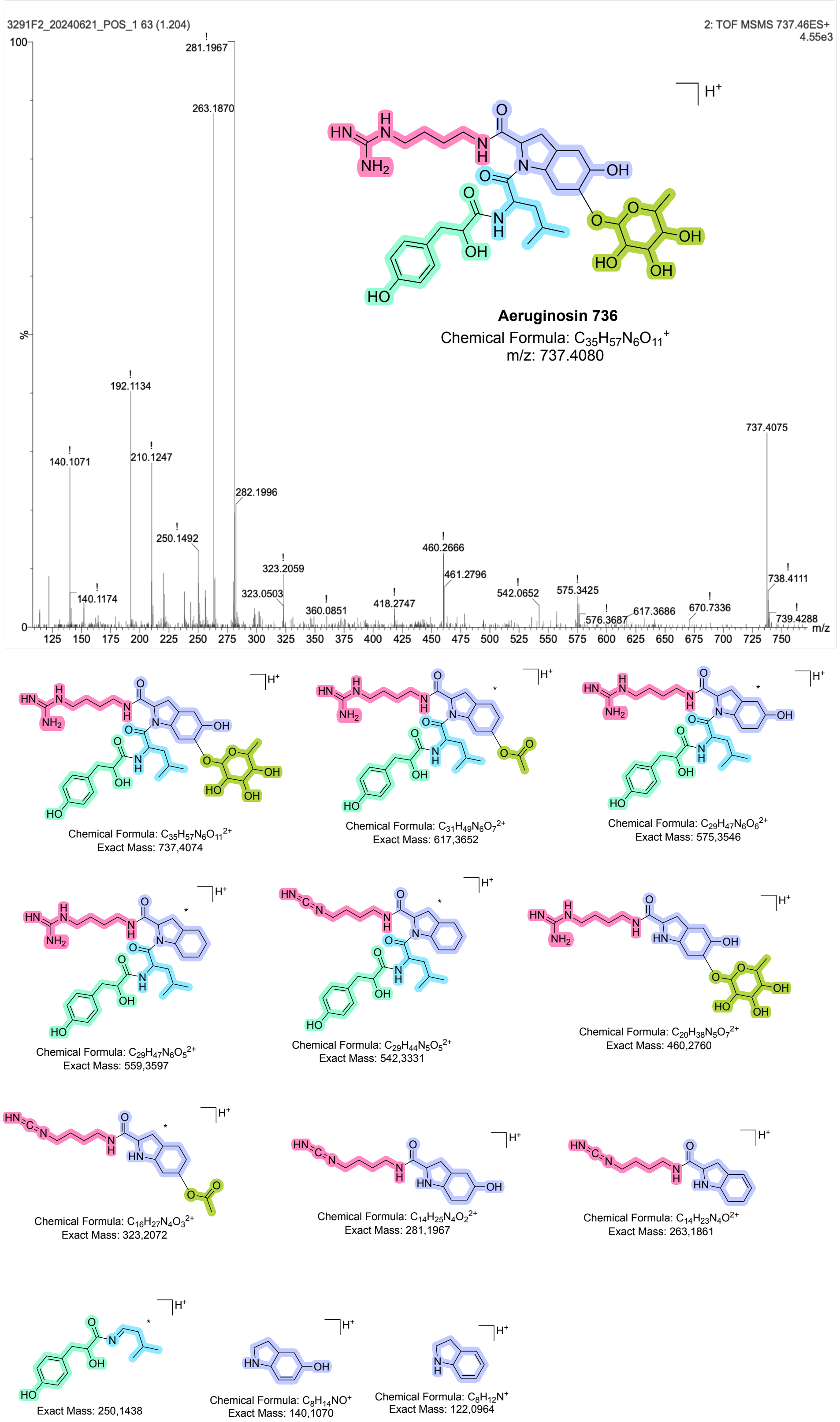

Figure S27. MS/MS spectrum and fragment ion annotation of aeruginosin 752

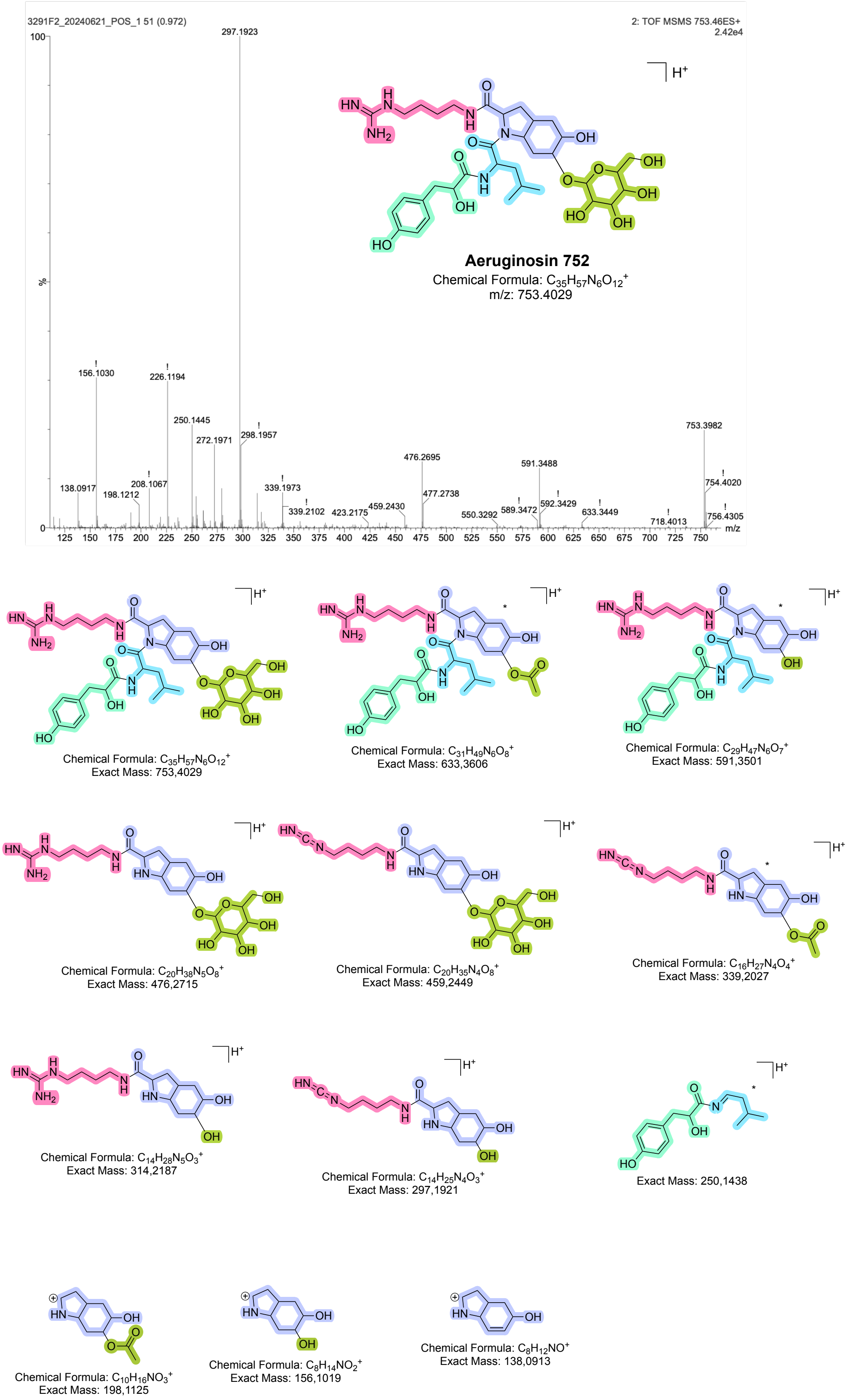

Figure S28. MS/MS spectrum and fragment ion annotation of aeruginosin 766

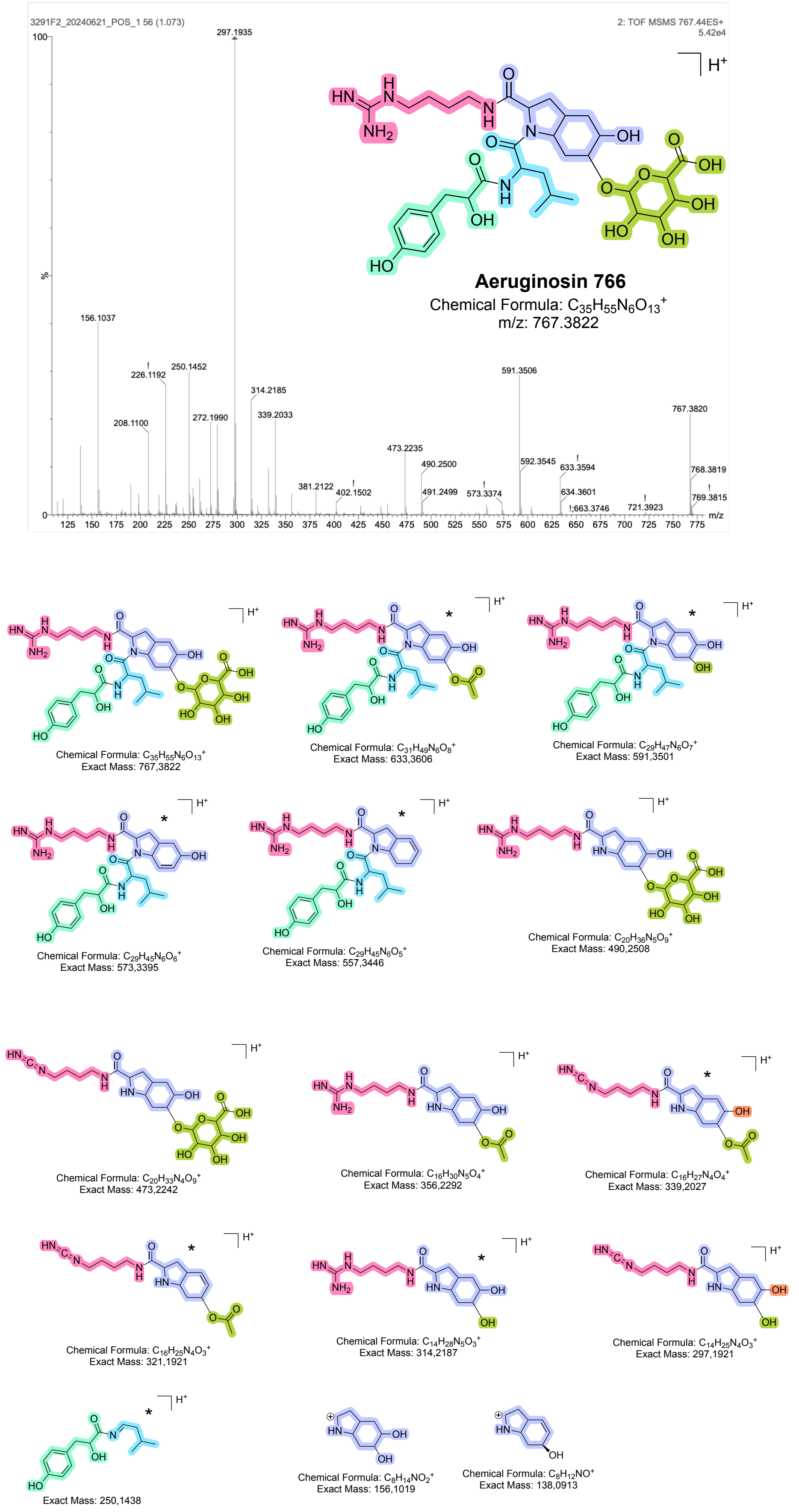

Figure S29. MS/MS spectrum and fragment ion annotation of aeruginosin 822

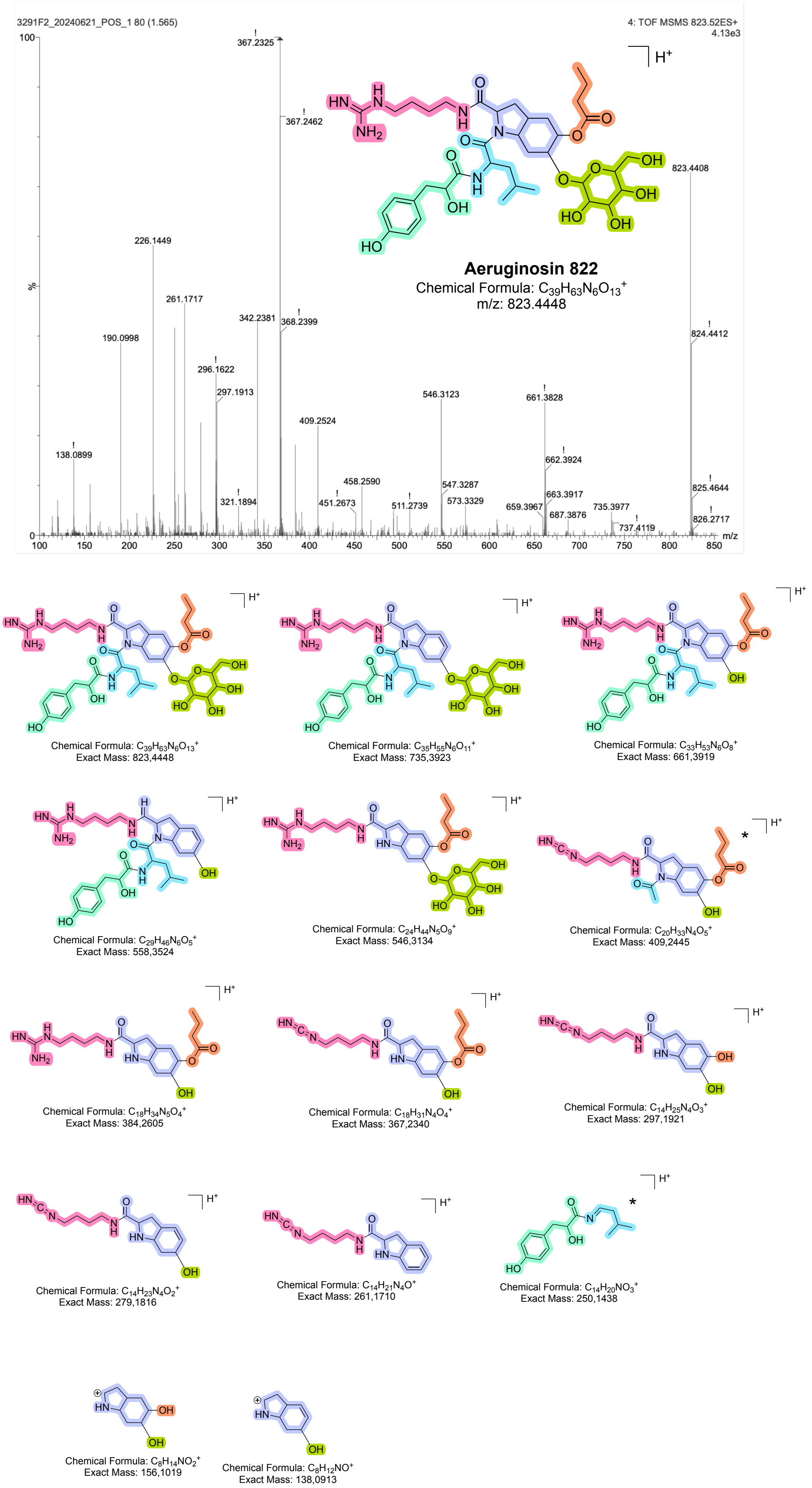

Figure S30. MS/MS spectrum and fragment ion annotation of aeruginosin 836

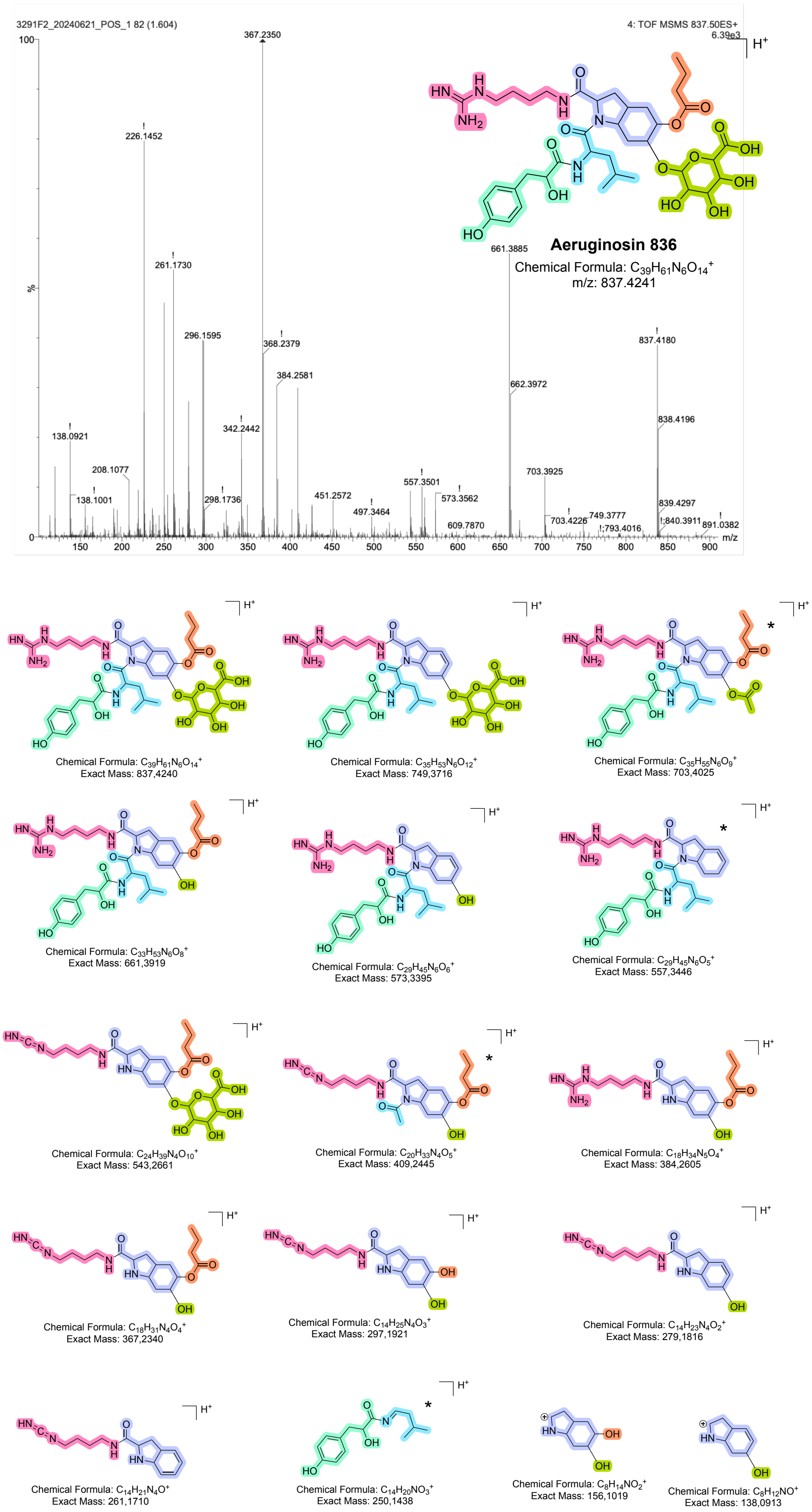

Figure S31. MS/MS spectrum and fragment ion annotation of aeruginosin 848

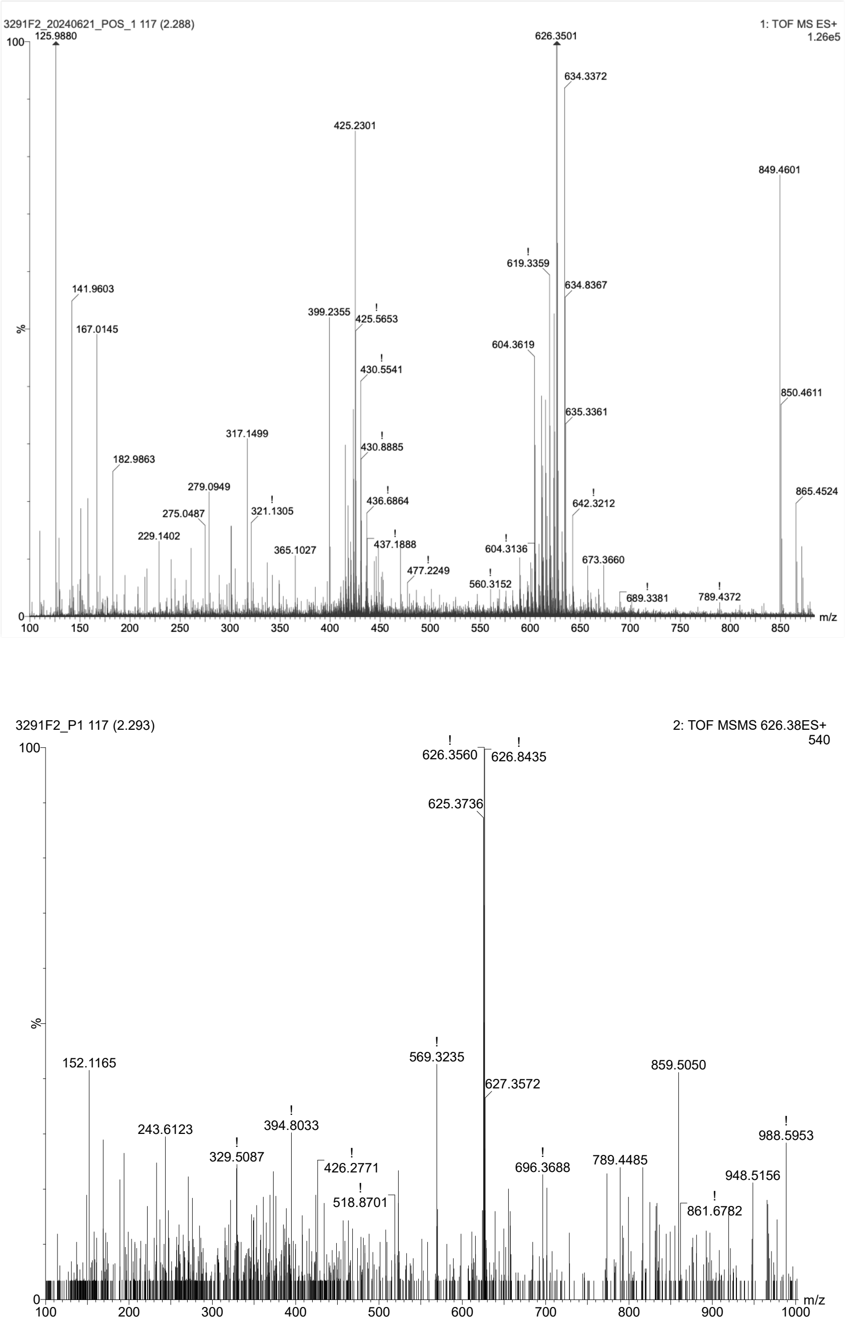

Figure S32. MS/MS spectrum and fragment ion annotation of aeruginosin 850

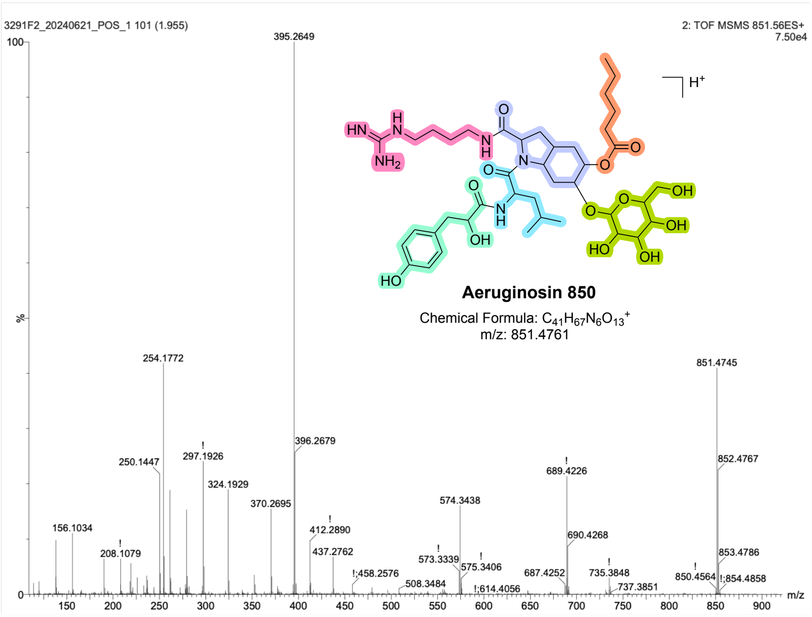

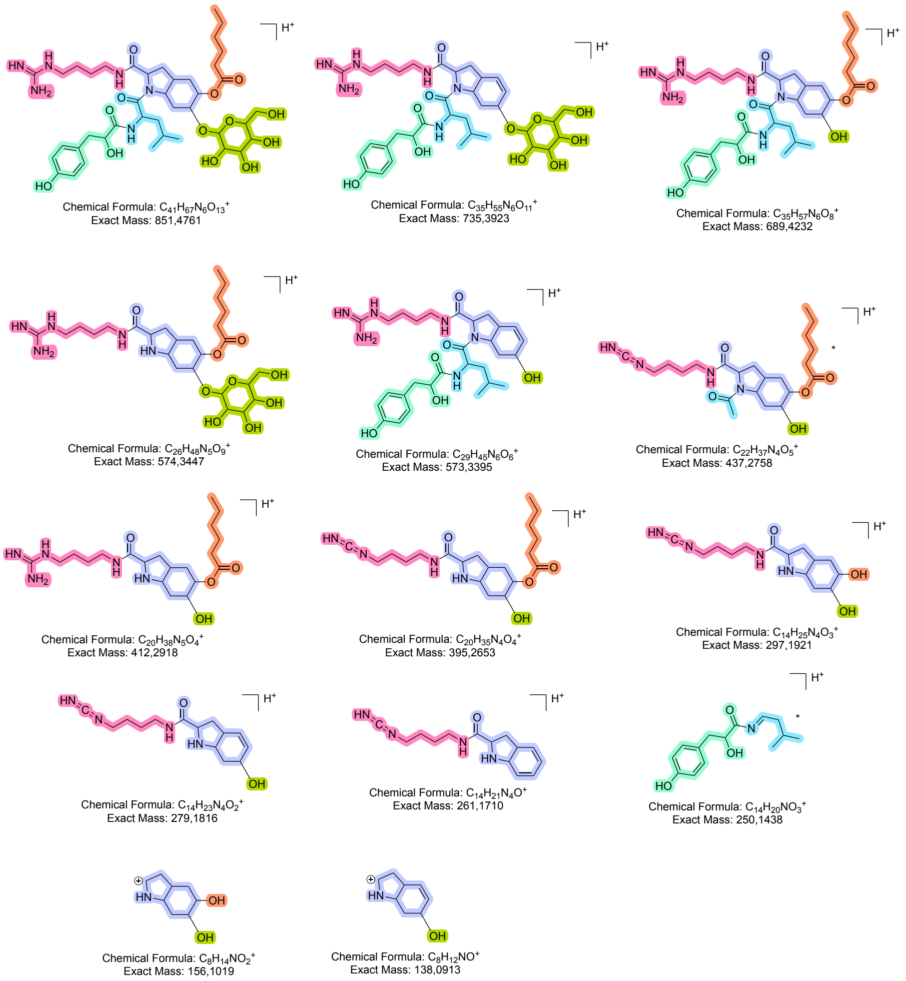

Figure S33. MS/MS spectrum and fragment ion annotation of aeruginosin 865

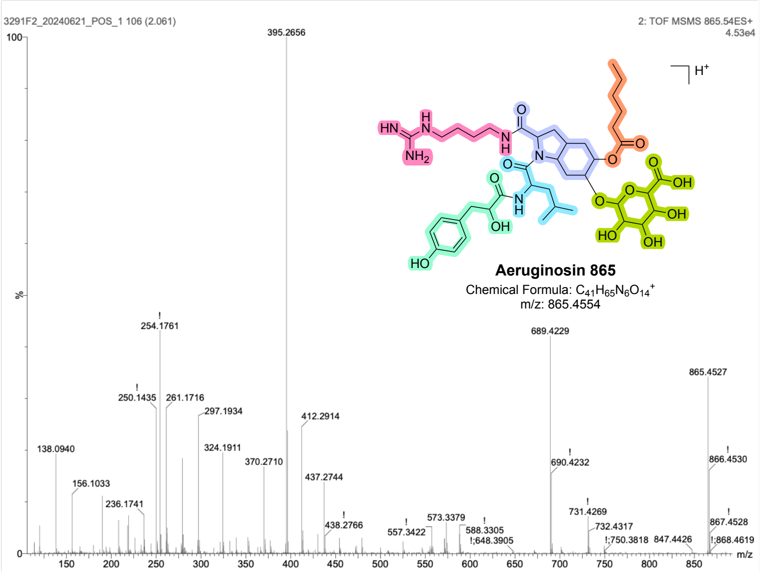

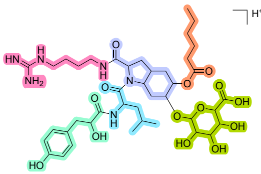

Chemical Formula: $C_{41}H_{65}N_6O_{14}^+$
Exact Mass: 865,4553

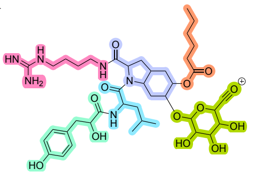

Chemical Formula: $C_{41}H_{63}N_6O_{13}^+$
Exact Mass: 847,4448

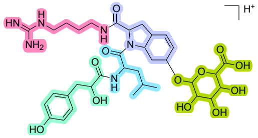

Chemical Formula: $C_{35}H_{53}N_6O_{12}^+$
Exact Mass: 749,3716

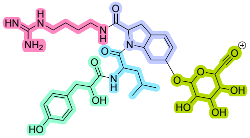

Chemical Formula: $C_{35}H_{51}N_6O_{11}^+$
Exact Mass: 731,3610

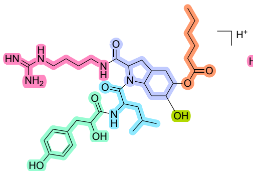

Chemical Formula: $C_{35}H_{57}N_6O_8^+$
Exact Mass: 689,4232

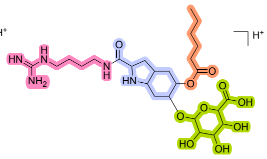

Chemical Formula: $C_{26}H_{46}N_5O_{10}^+$
Exact Mass: 588,3239

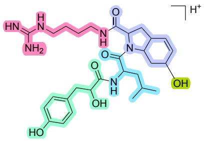

Chemical Formula: $C_{29}H_{45}N_6O_6^+$
Exact Mass: 573,3395

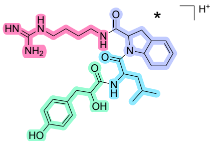

Chemical Formula: $C_{29}H_{45}N_6O_5^+$
Exact Mass: 557,3446

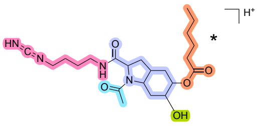

Chemical Formula: $C_{22}H_{37}N_4O_5^+$
Exact Mass: 437,2758

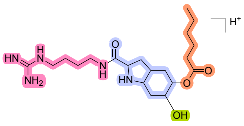

Chemical Formula: $C_{20}H_{38}N_5O_4^+$
Exact Mass: 412,2918

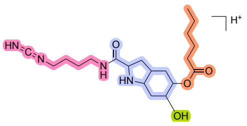

Chemical Formula: $C_{20}H_{35}N_4O_4^+$
Exact Mass: 395,2653

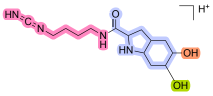

Chemical Formula: $C_{14}H_{25}N_4O_3^+$
Exact Mass: 297,1921

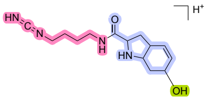

Chemical Formula: $C_{14}H_{23}N_4O_2^+$
Exact Mass: 279,1816

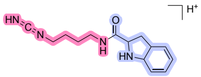

Chemical Formula: $C_{14}H_{21}N_4O^+$
Exact Mass: 261,1710

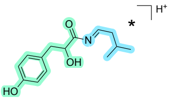

Chemical Formula: $C_{14}H_{20}NO_3^+$
Exact Mass: 250,1438

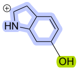

Chemical Formula: $C_8H_{12}NO^+$
Exact Mass: 138,0913

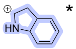

Chemical Formula: $C_8H_{10}N^+$
Exact Mass: 120,0808

Figure S34. MS/MS spectrum and fragment ion annotation of aeruginosin 878A

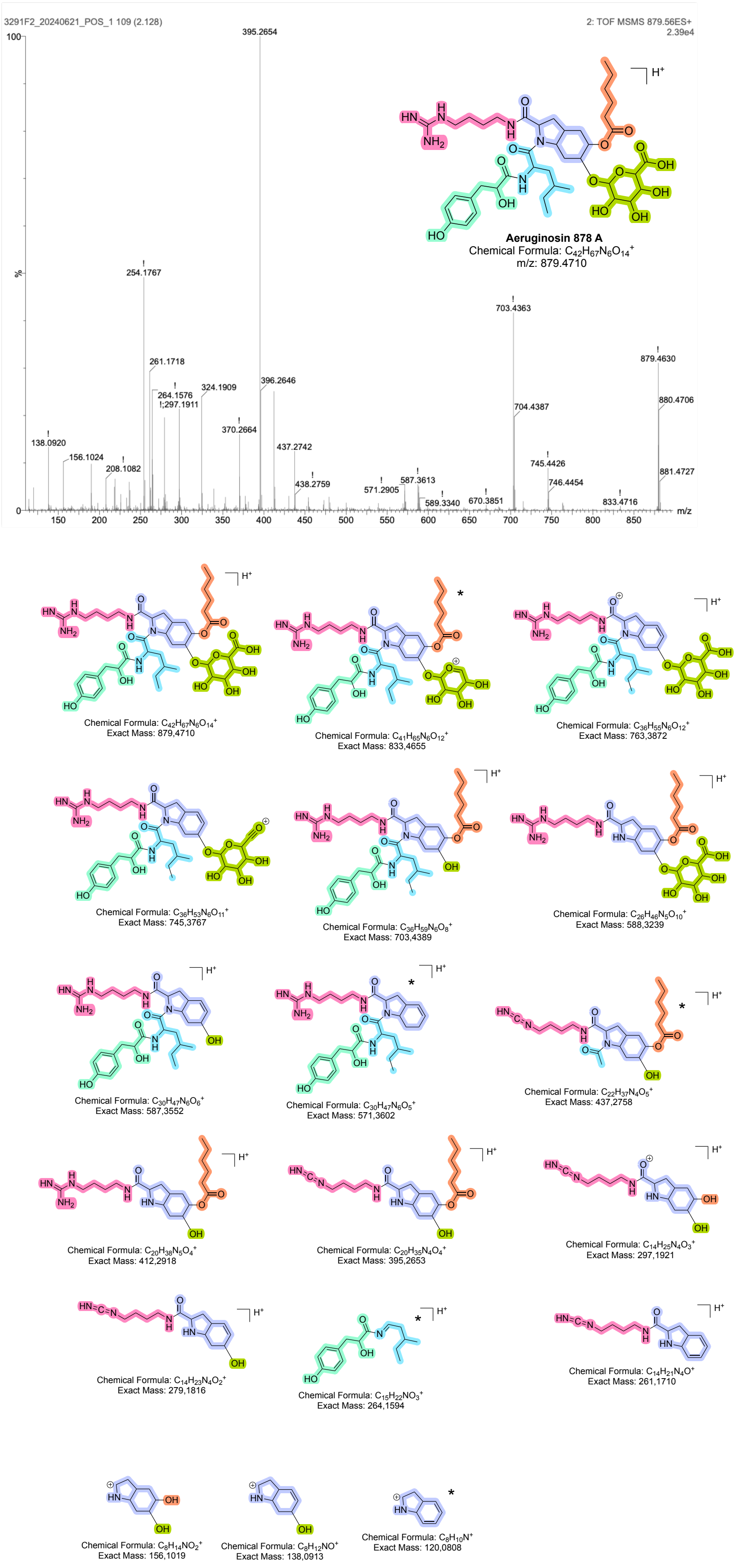

Figure S35. MS/MS spectrum and fragment ion annotation of aeruginosin 892

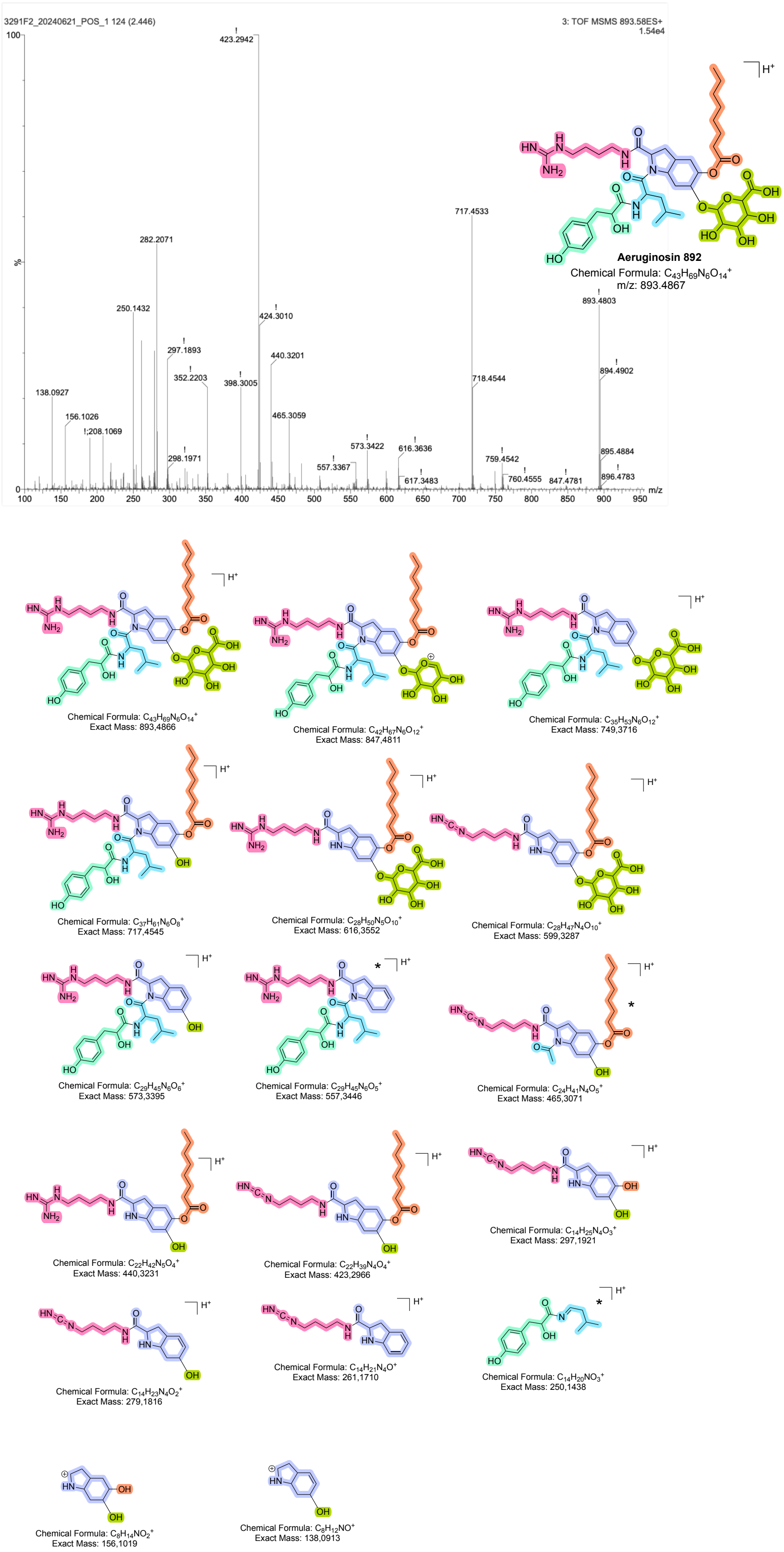

Figure S36. MS and MS/MS data of the potential novel aeruginosin with *m/z* 871.5018 $[M+H]^+$

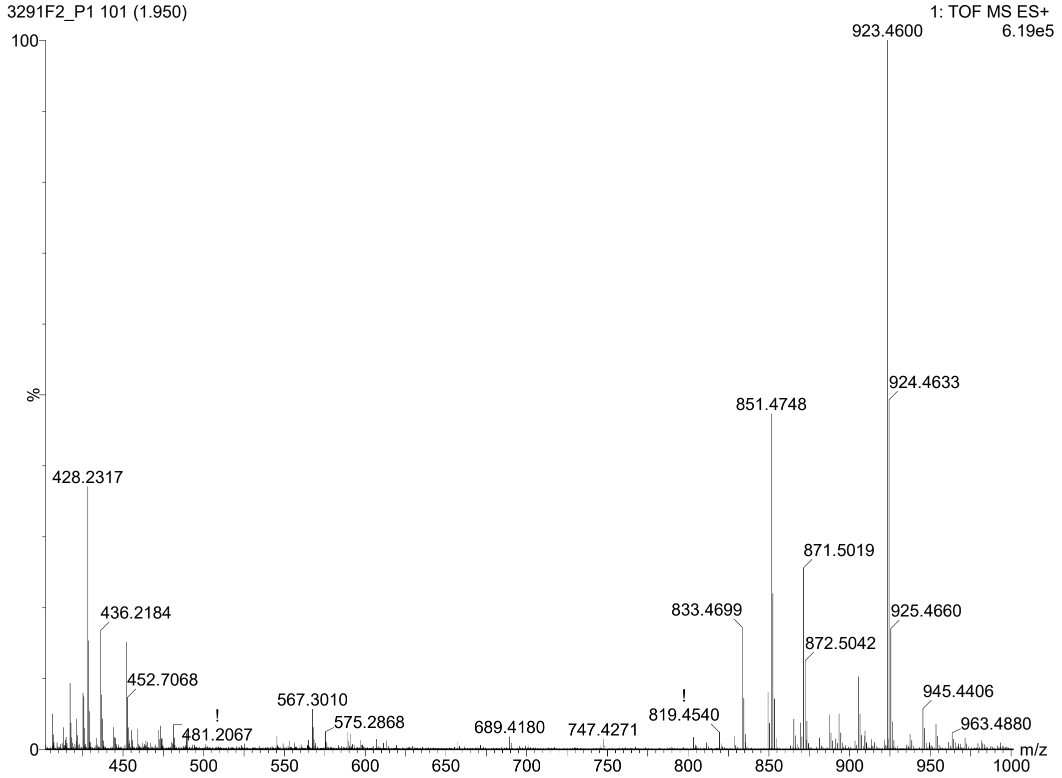

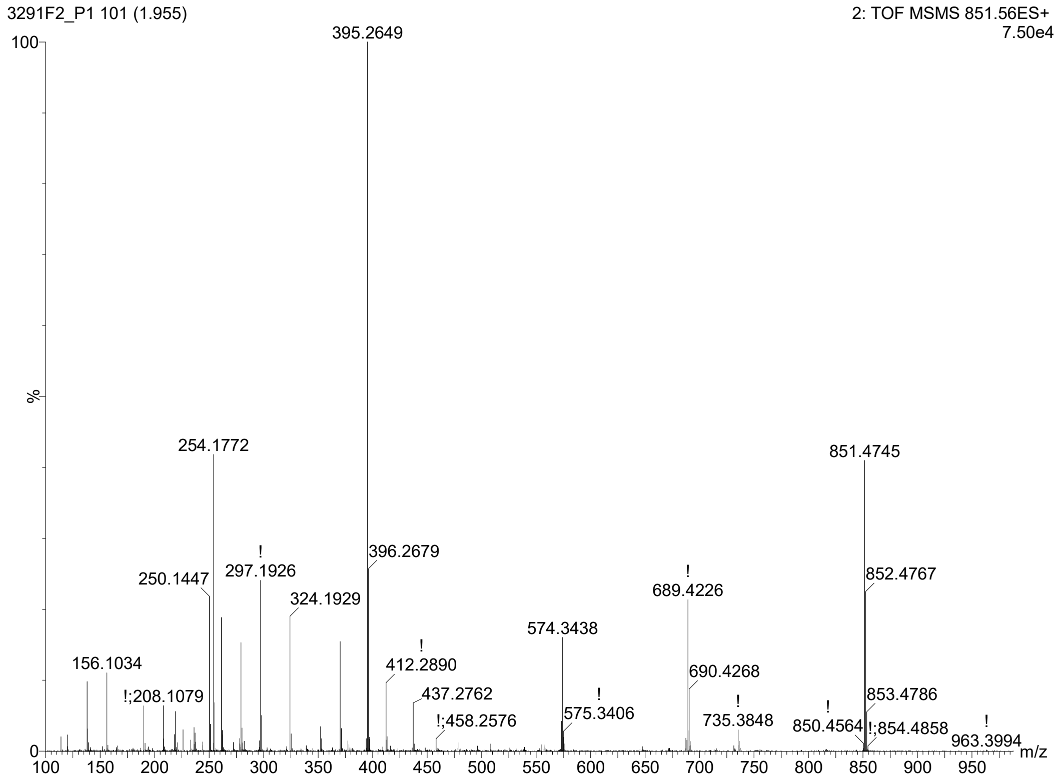

Figure S37. MS and MS/MS data of the potential novel aeruginosin with *m/z* 899.4602 $[M+H]^+$

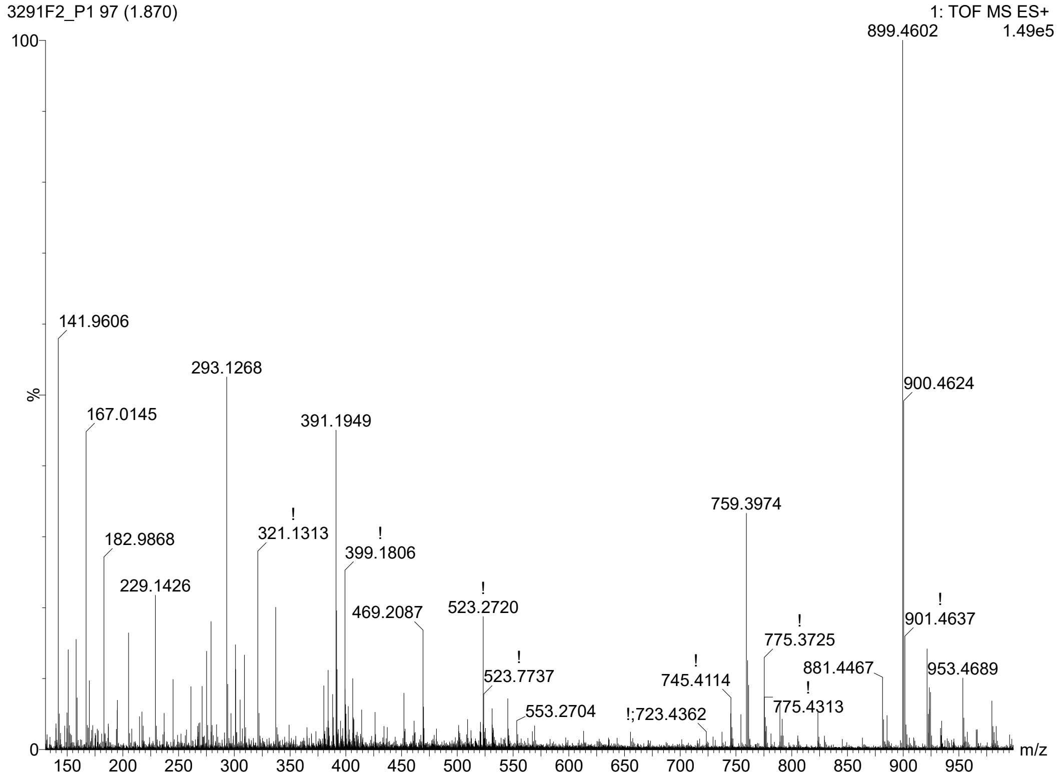

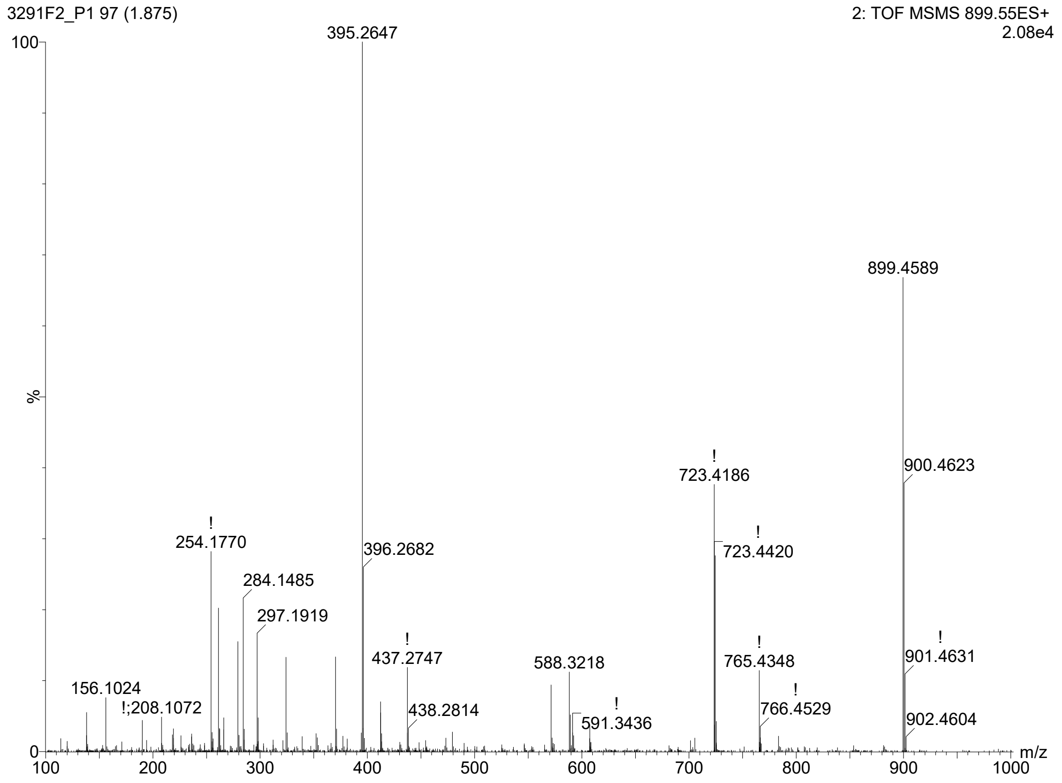

Supplement: Supplementary file 1 [file ao5c07322_si_001.pdf]
